# Optimization of Covalent MKK7 Inhibitors *via* Crude Nanomole-Scale Libraries

**DOI:** 10.1021/acs.jmedchem.1c02206

**Published:** 2022-07-30

**Authors:** Paul Gehrtz, Shir Marom, Mike Bührmann, Julia Hardick, Silke Kleinbölting, Amit Shraga, Christian Dubiella, Ronen Gabizon, Jan N. Wiese, Matthias P. Müller, Galit Cohen, Ilana Babaev, Khriesto Shurrush, Liat Avram, Efrat Resnick, Haim Barr, Daniel Rauh, Nir London

**Affiliations:** † Department of Chemical and Structural Biology, Weizmann Institute of Science, 7610001 Rehovot, Israel; ‡ Department of Chemistry and Chemical Biology, TU Dortmund University, Otto-Hahn-Strasse 4a, 44227 Dortmund, Germany; § Drug Discovery Hub Dortmund (DDHD) am Zentrum für integrierte Wirkstoffforschung (ZIW), 44227 Dortmund, Germany; ∥ The Nancy and Stephen Grand Israel National Center for Personalized Medicine, Weizmann Institute of Science, 7610001 Rehovot, Israel; ⊥ Department of Chemical Research Support, Weizmann Institute of Science, 7610001 Rehovot, Israel

## Abstract

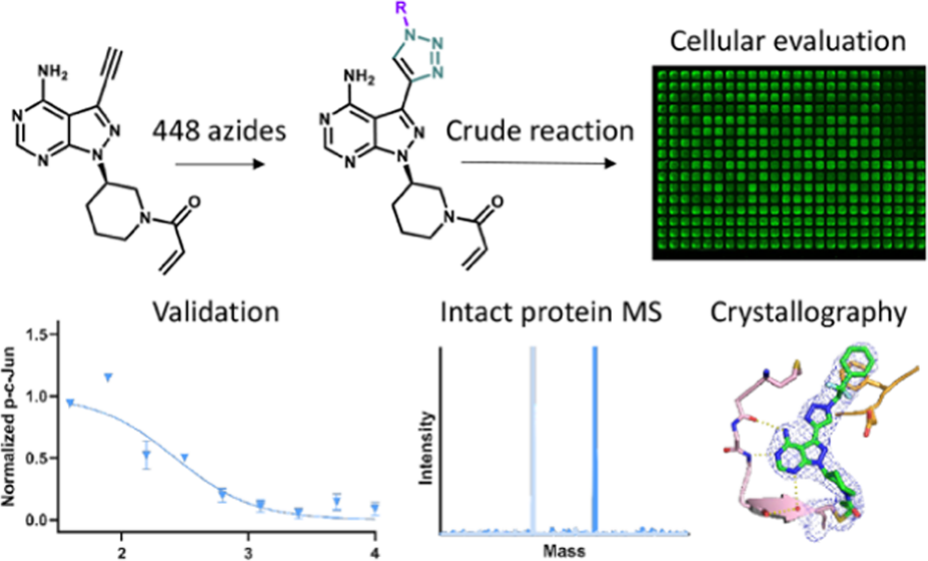

High-throughput nanomole-scale synthesis allows for late-stage functionalization (LSF) of compounds in an efficient and economical manner. Here, we demonstrated that copper-catalyzed azide–alkyne cycloaddition could be used for the LSF of covalent kinase inhibitors at the nanoscale, enabling the synthesis of hundreds of compounds that did not require purification for biological assay screening, thus reducing experimental time drastically. We generated crude libraries of inhibitors for the kinase MKK7, derived from two different parental precursors, and analyzed them *via* the high-throughput In-Cell Western assay. Select inhibitors were resynthesized, validated *via* conventional biological and biochemical methods such as western blots and liquid chromatography–mass spectrometry (LC-MS) labeling, and successfully co-crystallized. Two of these compounds showed over 20-fold increased inhibitory activity compared to the parental compound. This study demonstrates that high-throughput LSF of covalent inhibitors at the nanomole-scale level can be an auspicious approach in improving the properties of lead chemical matter.

## Introduction

A common challenge in medicinal chemistry is the exploration of a large and diverse set of analogues of an intermediate hit or lead compound. Access to such a set could guide the optimization of the series by efficiently deriving a structure–activity relationship (SAR) while improving the overall potency and physicochemical properties. Such derivatization is preferably achieved toward the end of the synthesis route, to maximize the utility of a single intermediate. This approach faces some challenges in the optimization of covalent inhibitors, which typically contain electrophilic warheads that are introduced by the acylation of a free amine.^1−6^ As most of these compounds display increased reactivity due to the electrophilic functionality, it is difficult to realize further transformations once the electrophilic moiety is introduced. In this work, we describe a workflow that allows late-stage functionalization (LSF) of acrylamide-based kinase inhibitors that is compatible with efficient large-scale cellular and biochemical assays for high-throughput testing of these compounds.

Reactions employing less than 300 nmol of starting material have been coined nanomole scale in the literature and offer potential benefits such as conservation of advanced intermediates and a reduction of solvent usage. A landmark achievement in this area has been reported by Merck Sharp and Dohme researchers who employed a variety of commonplace medicinal chemistry reactions (*e.g*., amide couplings and Suzuki reactions) in a high-throughput manner.^7,8^ Moreover, these crude reaction mixtures were directly fed into an affinity-selection mass spectrometry assay used to generate SAR information (termed NanoSAR^9^) that were followed up by validation experiments. The plate-based format allows closer integration of chemical and biological experiments but also comes with the limitation that reactions conducted at the nanomole scale are rarely appropriate for purification. For example, Gao et al.^10^ used crude reaction plates generated by the Groebke–Blackburn–Bienaymé reaction, with no purification, integrated with a biochemical assay. Sutanto et al.^11^ managed to use a dispensing system for high-throughput synthesis in multiple component reactions such as Ugi and Passerini reactions with a simple purification method. A related approach for synthesizing large and diverse combinatorial libraries that does not require purification of every compound is DNA-encoded library (DEL).^12−14^ However, the deconvolution of DEL screening is significantly more complicated on the one hand, and very few DELs have been applied for the discovery of covalent inhibitors on the other.^15,16^ Another limitation of DELs is that, in general, they cannot be screened in cells but only biochemically.

The acrylamide functionality of small-molecule covalent inhibitors interferes with many traditional (Michael addition) and modern transition-metal-catalyzed reactions^17,18^ including pericyclic reactions.^19^ Since the copper(I)-catalyzed alkyne–azide cycloaddition (CuAAC)^20^ facilitates a general electrophile-tolerant transformation of terminal alkynes toward 1,4-substituted 1,2,3-triazoles, we hypothesized that this robust, air- and water-tolerant synthetic methodology might be suitable for LSF at the nanomole scale^7^ in a plate-based format. Although the strategy of screening crude preparations and validating by resynthesis has been employed both on biophysical and biochemical levels with success in various reports,^10,21^ crude libraries evolved from CuAAC chemistry have not been directly evaluated in the cellular context yet. In this work, we applied this emerging technology toward the optimization of covalent kinase inhibitors targeting the JNK pathway by high-throughput nanomole-scale synthesis coupled with in-depth validation of the crude mixtures by a variety of biological experiments using acoustic droplet ejection.^10,22^

The JNK pathway is activated during cell stress and is implicated in various pathologies,^23^ such as Parkinson’s disease,^24^ Alzheimer’s disease,^25^ cancer,^26^ and inflammatory diseases.^27,28^ Similar to other mitogen-activated protein kinases (MAPKs), activation of the JNK pathway includes a signaling cascade that involves MAP2K and MAP3K. The direct activators of JNKs are the MAP2Ks MKK7 and MKK4.^29^ Targeting upstream kinases of MKK4/7 has been met with limited success due to possible activator redundancy, whereas targeting JNK itself has not shown clinical success yet, perhaps due to on-target toxicity. Thus, we considered the protein kinase MKK7, which exclusively phosphorylates JNK (whereas MKK4 also cross-talks with the p38 pathway^30^) would be a prime target for selective JNK pathway inhibition. Recent results showcase the possibility of developing allosteric ligands of MKK7 as well as type II-covalent inhibitors (expanding toward the so-called back pocket of the kinase)^31^ as well as dual covalent MKK4/7 inhibitors.^32^ We have previously developed selective acrylamide inhibitors for MKK7^33,34^ and set out to use them as starting points for optimization.

## Results and Discussion

**Figure 1:**
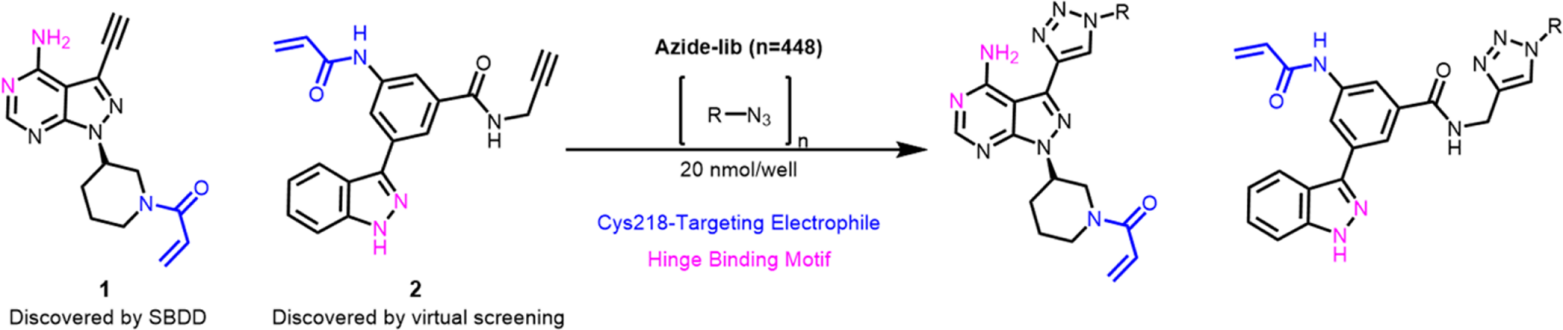
Synthesis of libraries of derivatives of **1** and **2** by CuAAC between the starting alkynes and a 448-member azide library (**Azide-lib**; Dataset S1). Reaction conditions: **1** or **2** (20 nmol, 8 mM), **Azide-lib** (20 nmol, 8 mM in dimethyl sulfoxide (DMSO)), 1:1 CuSO_4_·5H_2_O/TBTA (25 mol %, 2 mM), ascorbic acid (NaAsc; 40 nmol, 16 mM in H_2_O), H_2_O/DMSO (1:1 v/v), 25 °C, 24 h, in 384-well plates assembled from freshly made (<1 h) stock solutions by an Echo 555 acoustic dispensing system under air.

To investigate the high-throughput LSF of covalent inhibitors, we selected two distinct covalently acting chemotypes (**1**, **2**; Figure 1) targeting MKK7 with appreciable biochemical potency^33,34^ as starting points for our nanoscale synthesis workflow. Pyrazolopyrimidine **1**,^34^ closely related to ibrutinib,^35^ has been rationally designed by structural considerations from EGFR-targeting molecules. Compound **1** covalently binds Cys218, while its terminal alkyne protrudes into the so-called “back pocket” of MKK7 (PDB: 6IB0; Figure S1A). In a previous report, the Rauh group demonstrated LSF of **1** by means of CuAAC in the presence of the acrylamide functionality and isolated a small number of compounds in preparative scale before assaying, resulting in potent triazole-linked MKK7 inhibitors with high selectivity over EGFR^WT^.^34^ Therefore, we found **1** to be an ideal starting point to create a large triazole library in the crude state. The core molecular features of indazole **2**(33) have been discovered by the London lab *via* a virtual covalent screening campaign.^33^ Compound **2** also binds Cys218 covalently; however, in this case, the alkyne was initially designed as a handle for chemical biology experiments since it pointed outward from the ATP-binding pocket and toward the solvent (PDB: 7CBX;^36^Figure S1B). We envisioned that by generating triazole diversification in this area, we could either find interactions with surface-exposed residues or install a convenient handle for solubility or tag it with an exit vector. Such tags could be used to develop a bifunctional molecule such as a PROTAC.^37,38^ The two MKK7-targeting scaffolds were reacted *via* CuAAC with a library of 448 commercially available azides (**Azide-lib**; Dataset S1, Figure 1).

To enable the application of the crude reaction mixture in subsequent biological experiments, we used DMSO as the reaction solvent, and a catalytic system of CuSO_4_ and the ligand tris((1-benzyl-4-triazolyl)methyl)amine (TBTA).^39^ Such Cu/ligand combinations are known to stabilize the catalytically active Cu(I) state and are able to combat highly coordinating environments.^40^ Water (50% v/v) was required as a co-solvent to solubilize the sodium ascorbate (NaAsc) necessary for the stabilization of the Cu(I) state and allowed us to pursue a cellular assay. Using an acoustic droplet ejection robotic system (Echo 555), we set up these reactions in standard 384-well plates compatible with further acoustic dispenser handling. After dilution of **1** and **2**, we set aside a fraction of each synthesized compound from both libraries for analysis by an In-Cell Western assay (ICW).^33,41^ ICW is a high-throughput cellular assay in which cells can be immuno-stained against a query target (in this case phospho-JNK), and in parallel, the number of cells is quantified for normalization. This allows a quantitative readout for numerous cellular experiments at the same time.

**Figure 2:**
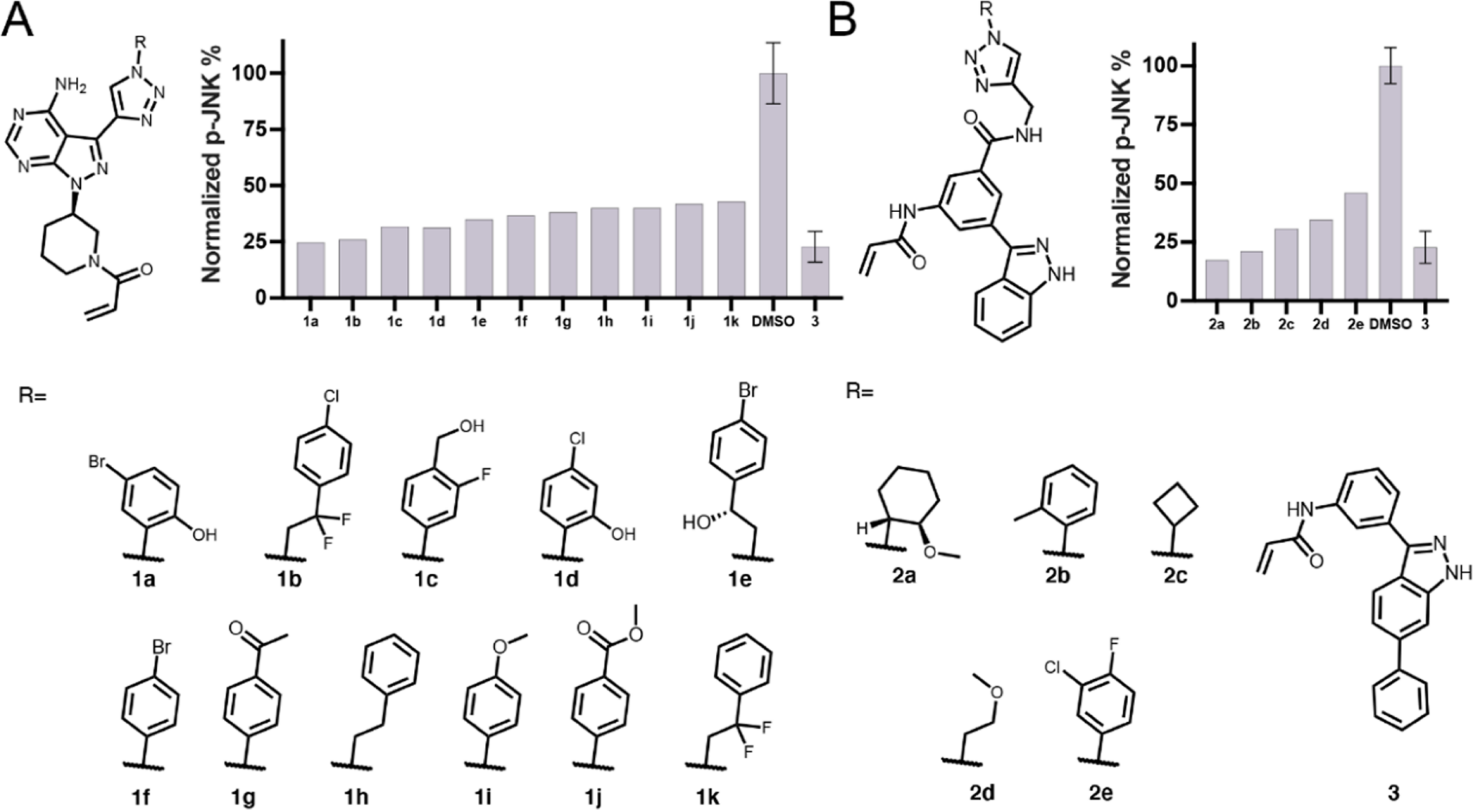
Primary screening of crude triazole derivatives by ICW. The assay was performed with U2OS cells, 2 h after treatment. The ICW was performed at 13.8 μM for series **1** and at 10 μM for series **2**. (A) The most active compounds of the **1** series in the primary ICW, and their normalized levels of p-JNK in cells. (B) Selected compounds from the **2** series evaluated by ICW, and their normalized levels of p-JNK in cells. For series **2**, the selected compounds are not necessarily the most active ones.

The ICW comprised incubation of U2OS cells with the two crude compound libraries followed by treatment with sorbitol to induce osmotic stress and activate the pathway,^42^ which culminates in the phosphorylation of JNK by MKK7. After incubation with the individual test compounds, the normalized amount of phosphorylated JNK (p-JNK) was quantified. The eleven most potent compounds from the primary screen of triazolyl derivatives of **1** caused decreased p-JNK levels by 65–80% compared to untreated controls (Figure 2A). Satisfyingly, pure **1g** was previously reported to show high potency in a cellular assay system, effectively reducing p-JNK levels detected by western blot analysis,^34^ thereby independently confirming this screening result.

Two common structural features were enriched in the top hits originating from derivatization of **1**: halogenated hydroxyphenyls and phenethyl derivatives (Figure 2A). Most of the phenyl rings were substituted with H-bond acceptor qualities (**1f**, **1g**, **1i**, **1j**) in *para*-position and/or a hydroxyl group in the *ortho*-position (**1a**, **1d**). The positioning of these phenolic groups appears to overlap in space with the alcohol of **1e**, hinting at a possible H-bonding opportunity present in both discussed motifs. Collectively, these compounds fall within a relatively narrow range of beneficial calculated physicochemical properties (clog *S* −5 to −3, clog *P* 0–3, and tPSA < 150 Å^2^, Figures S2–S4) which may contribute to their improved performance in the ICW screening.

Five members of series **2** were selected from the top inhibitors according to the ICW (Figure 2B), taking into account, in addition to inhibition, commercial availability, calculated physicochemical properties (Figures S5–S7), with the aim to examine possible linker architectures and exit vectors for the development of bifunctional ligands from the identified MKK7 binders. It is worth noting that ether (**2a, 2d**) is a typical structural element in linkers in functionalized molecules, and if it is well tolerated, it may offer a route to construct corresponding bifunctional analogues. In general, *N*-phenyltriazoles (**2b**, **2e**) also appear to be tolerated.

**Figure 3:**
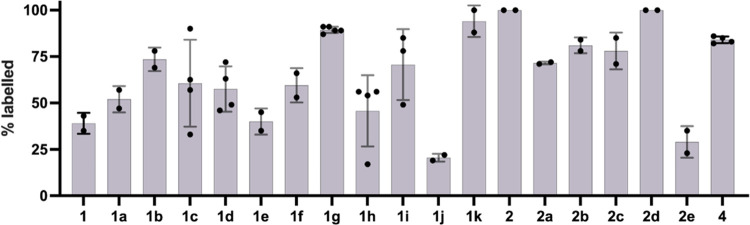
% MKK7 labeling by series **1** and **2**. Intact protein liquid chromatography/mass spectrometry (LC/MS) labeling experiment of resynthesized compounds derived from top crude screening hits. Reaction conditions: 2 μM compound, 2 μM protein, 5 μM ATP, 5 mM MgCl_2_, 10 min, 4 °C, quenched with formic acid to a final concentration of 0.4% (v/v).

Compounds **1a–k** (excluding **1g** which was previously reported^34^) and **2a–e** were subsequently resynthesized, purified, and evaluated in depth. While we did not get an opportunity to compare the activities of crude and pure compounds in the ICW assay, we hypothesized the pure compounds should be more potent. We first evaluated the irreversible labeling of recombinant MKK7 with the selected compounds. With incubation times of 16, 4, and down to 1 h almost all of the selected compounds labeled the protein quantitatively. Even with reaction times as short as 10 min, >70% labeling was observed for all compounds (Figure S8). Finally, using more stringent reaction conditions (2 μM compound, 2 μM protein, 5 μM of ATP, and 5 mM MgCl_2_ for competition, 10 min, 4 °C), we were able to detect differential labeling that highlighted how the different azides affected binding (Figure 3). In the **1** series, **1k** is the only compound that showed improved binding in comparison to reference compound **1g** (94 *vs* 89% labeling; Figure 3), despite showing the least inhibition in the primary assay. In addition to **1k**, **1b**, a close analogue to **1k**, had on average the second highest labeling in the series. Most compounds retained over 50% labeling under these very stringent conditions. In the **2** series, both **2d** and the precursor **2** show 100% labeling under all conditions we investigated and were better than the reported covalent binder **4**(33) (Figure S15) that was previously used as a comparison to compound **2**. The other compounds showed lower labeling, perhaps implying that the introduced triazolyl moiety might hinder the binding compared to the alkyne. Still, except for **2e**, all of the compounds retained over 70% labeling. In general, both series **1** and **2** showed significant inhibition in the primary assay and very potent irreversible binding to the recombinant protein. It may be that binding differences under such stringent conditions do not translate to differences in a cellular assay as other factors that control cellular efficacy, such as, *e.g.*, permeability, stability, and reactivity, are more prominent. Still, the one compound with a markedly lower potency in the ICW, **2e**, also showed the lowest binding ability out of series **2**.

We should note that while direct reduced analogues of the reported compounds were not synthesized and tested here as negative control compounds, very close noncovalent analogues were already synthesized and tested by us (for series **1**: compound **4b**;^34^ for series **2**: compound **MKK7-NEG-1**(33)) and were shown to be inactive as MKK7 inhibitors.

**Figure 4:**
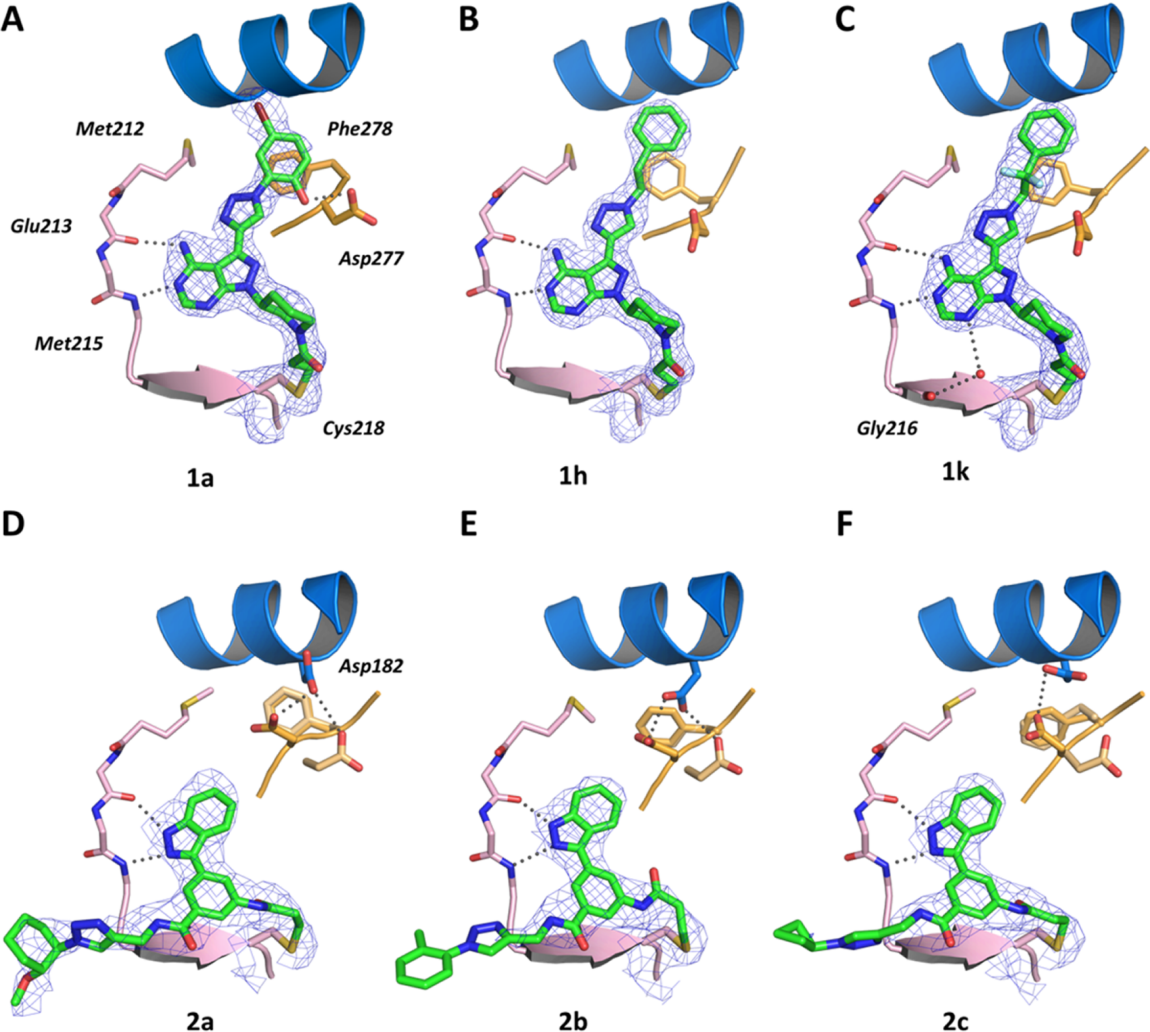
Crystal structures of triazolyl-decorated members of series **1** and **2** in complex with MKK7. Diagrams of the experimental electron densities and modeled complex structures of (A) **1a** (7OVK) (B) **1h** (7OVI), (C) **1k** (7OVJ), (D) **2a** (7OVL), (E) **2b** (7OVN), and (F) **2c** (7OVM) at resolutions ranging from 1.95 to 2.9 Å with the 2mFo-DFc maps (blue) contoured at 1.0σ. Hydrogen-bond interactions of the ligands with the protein are illustrated by gray dotted lines.

To explain the SAR and validate our design approach, we subjected the best performing compounds to protein crystallization studies following both direct co-crystallization and soaking protocols. We successfully determined six novel MKK7-ligand complexes with three members of each series: **1a**, **1h**, **1k** and **2a**, **2b**, **2c** (Figure 4; Figure S9; Table S1). Partial electron density corresponding to three more ligands of the **1** series (**1b**, **1d**, **1e**) was identified in corresponding complexes. In these cases, the inhibitors were situated within the binding site but appeared to miss the introduced triazolyl moiety (data not shown). The systematic absence at this position in all three cases likely indicates synchrotron irradiation damage during the diffraction experiment.

Throughout series **1**, the 4-amino-pyrazolopyrimidine core forms the expected hinge contacts to Glu213 and Met215 (Figure 4A–C), as well as a covalent bond between the piperidine-linked acrylamide and the thiol side chain of Cys218. The moieties introduced in the 3-position of the scaffold by CuAAC point toward the α-helix C and occupy the back pocket of the active binding site. The *ortho*-phenolic function of **1a** forms an additional hydrogen bond with Asp277 of the DFG motif. Compared to the structure of apo-MKK7 (PDB: 6QFL),^43^ Asp182 is pushed away as a result of giving room for the extended compounds and in some cases (as for **1k**) the exact location of which could not be determined by means of the difference in electron density. In all three complexes, the enzyme is present in the active DFG-in conformation stabilized by the bound ligand, consistent with typical characteristics of type-I kinase inhibitors. Taken together, these observations agree with previous reports on **1g**.^34^

As for series **2**, the indazole scaffold interacts with the hinge backbone-NH of Met215 and carbonyl of Glu213 (Figure 4D–F). Furthermore, the NH of the triazolyl-linking amide forms a hydrogen bond with Met215-carbonyl, which has been previously observed for alkyne **2**(36) as well. While all three structures also show the covalent modification of Cys218 by the inhibitor’s acrylamide moiety, the triazole-linked moieties point toward the solvent and are not clearly defined. Comparably high B factors indicate conformational freedom of the corresponding atoms. Accordingly, the kinase is present in the DFG-in conformation as Phe278 is pointing toward α-helix C, and based on the difference in electron density, Asp277 is unconventionally present in two distinct conformations.

With respect to the initial design, the triazole moiety does not appear to form additional constructive interactions either inside the back pocket (series **1**) or in the solvent-exposed periphery of the binding site (series **2**). However, the various substitutions of series **1** were able to make new productive interactions in the back pocket. We successfully validated active site binding and covalent bond formation between the screening hits and Cys218, substantiating a rational LSF approach of covalent inhibitors through CuAAC chemistry.

**Figure 5:**
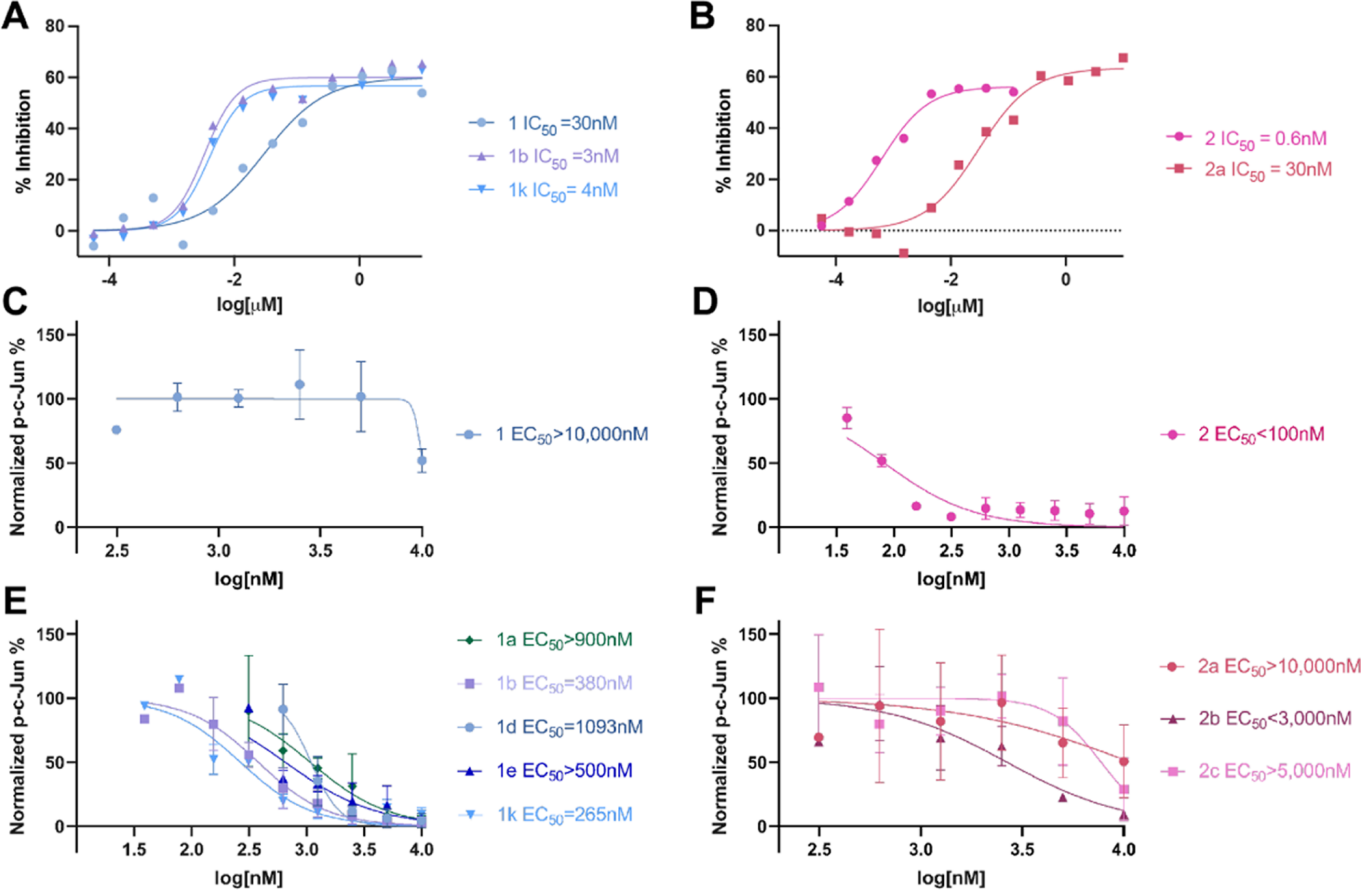
Biochemical and cellular activity of MKK7 inhibitors. (A, B) *in vitro* kinase activity assays for compounds (A) **1**, **1b**, and **1k** and (B) **2** and **2a** (see Figure S12 for full inhibition curve of **2**). (C–F) Quantification of dose–response c-Jun phosphorylation inhibition by a western blot assay: (C) **1**, (D) **2**, (E) analogues **1a**, **1b**, **1d**, **1e**, and **1k**, and (F) analogues **2a**, **2b**, and **2c**. See Figure S10 for the raw WB analysis.

To further assess the cellular potency of the top compounds that underwent successful co-crystallization, we performed detailed *in vitro* kinase activity assays and dose–response western blot analysis in U2OS cells, using downstream phosphorylated c-Jun (p-c-Jun) as a readout (Figures 5 and S10). All selected compounds derived from **1** showed a significant improvement in their inhibitory ability compared to the parent scaffold. This is in direct contrast to series **2** where the opposite behavior was observed.

In an *in vitro* kinase activity assay alkyne **1** was shown to be 10-fold less potent than the two best inhibitors among the series **1** compounds with IC_50_ = 30 nM for **1***vs***1b** IC_50_ = 3 nM and **1k** IC_50_ = 4 nM. This was also reflected in a cellular assay to an even larger extent. While the alkyne precursor **1** showed an EC_50_ of greater than 10 μM, both **1b** and **1k** showed sub-μM inhibition (EC_50_ = 380 nM and 265 nM, respectively), but also showed increased lipophilicity (Δclog *P* = 2.77 and 2.06; Table 1). Interestingly, these two inhibitors are structurally similar, only showing differences in the presence of *para*-Cl in **1b**. We should note that these derivatives are slightly more potent in comparison to previously reported **1g** both *in vitro* (IC_50_ = 10 nM for **1g**) and in cells (EC_50_ ∼400 nM for **1g**).^34^

**Table 1:** Potency and Lipophilic Efficiency of Selected Compounds Relative to Their Respective Alkyne^a^^,^^b^

compound	EC^50^ [nM]	Δclog *P*	ΔLipE
**1**	>10,000	0	0
**1a**	>900	+ 2.36	–1.39
**1b**	380	+2.77	–1.25
**1d**	1093	+2.22	–1.20
**1e**	>500	+1.22	–0.05
**1k**	265	+2.06	–0.29
**2**	<100	0	0
**2a**	>10,000	+0.44	–2.72
**2b**	<3000	+1.78	–3.48
**2c**	>5000	+0.21	–2.37

a clog *P* was calculated using DataWarrior.

b LipE was calculated as pIC_50_ – clog *P*.

Considering this result along with the structural evidence obtained for derivatives of **1**, the increase in cellular potency is most likely driven by additional lipophilic interactions in the MKK7 back pocket. Most of the potent compounds showed a reduction in lipophilic efficiency compared to the parent alkyne. Moderately potent compound **1e** was an exception, where a change in lipophilic efficiency compared to the parent alkyne remained close to zero. It is reasonable to speculate that the hydroxyl function of **1e** forges an additional hydrogen bond to Asp277 as does the phenol function in **1a** or to Lys155 (Figure S11).

On the other hand, series **2** compounds showed significantly lower activities compared to the highly potent alkyne **2**. In the *in vitro* kinase activity assay alkyne **1** showed very potent inhibition (IC_50_ = 0.6 nM; Figures 5B and S13), whereas its derivative **2a** decreased in potency (IC_50_ = 30 nM; Figure 5B). A similar trend was seen in cells where **2** was highly potent (EC_50_ < 100 nM; Figure 5D) with its derivatives losing potency and ranging in EC_50_ from around 3 μM (**2b**) to over 10 μM (**2a**). While the co-crystal structures suggested that the triazolyl substitution does not form new interactions with the protein, they do not explain such a decrease in potency, nor do the recombinant protein labeling results suggest significantly lower binding ability (Figure 3). A possible explanation is that the introduction of the triazolyl moiety at this position may compromise other aspects of cellular activity. One such factor may be sensitivity to deactivation by cellular glutathione (GSH).

We speculated that the triazole formation in both series will most likely not affect the reactivity of the intrinsic warhead since these adducts are distal to the electrophile and are not conjugated to it. To assess this possibility, we performed GSH consumption assays on compounds **1**, **1b**, **1k**, **2**, and **2b** (Figure S12). The thiol reactivity of **1b** and **1k** as approximated by GSH half-life *t*_1/2_ was decreased (*t*_1/2_ = 9.5 and 10.6 h, respectively) with respect to the original alkyne scaffold **1** (*t*_1/2_ = 7.7 h). **2b** however showed only slightly reduced reactivity compared to **2** (68 min compared to 57 min), whereas both were clearly more reactive than the series **1** compounds tested (Figure S12). A likely explanation for the increased reactivity of series **2** is the intrinsic reactivity of the aniline-based acrylamide compared to the piperidine-based acrylamide in series **1**.^44^

To assess whether these reactivity trends translated to proteomic selectivity we used alkynes **1** and **2** in pull-down proteomics experiments to identify potential off-targets. U2OS cells were incubated with DMSO or 5 μM of either alkyne for 2 h. Following lysis, the samples were “clicked” with azide-biotin, enriched on streptavidin beads, and proteins were quantified by LC/MS/MS (Dataset S2a). In accordance with our GSH reactivity assays, alkyne **2** was markedly more reactive and pulled down over 700 different proteins (Dataset S2c; Figure S14), whereas alkyne **1** was only able to significantly pull down two off-targets (Dataset S2b; Figure S14). We note that for neither alkyne MKK7 was detected, presumably resulting from its low expression levels.

From the cellular results, it appears that the solvent-exposed moieties caused a significant penalty in cellular potency across series **2**. We had hoped that some library members would form interactions with some of the solvent-exposed amino acids, and would allow us to identify possible exit vectors to use as a blueprint for PROTAC or other bivalent compound designs. Regaining some affinity from kinase back-pocket filling in future bivalent designs may compensate for potency losses by exiting the pocket *via* triazole chemistry. On the other hand, the fact that the GSH half-life improved shows that certain properties can still be tailored by our approach.

The top two inhibitors **1b** and **1k** were next selected for absorption, distribution, metabolism, and excretion (ADME) profiling (Table 2). The compounds showed comparable values for permeability across an artificial membrane, and intrinsic clearance as well as plasma stability in both mouse and human microsomes and plasma after 60 min exposure for all. Candidate **1k** showed slightly better kinetic solubility over **1b** and a lower export from CaCo-2 cells as indicated by an efflux ratio greater than 2. In direct comparison with previous data collected for **1g** and the structurally similar drug ibrutinib, **1b** and **1k** showed improved kinetic solubility (97.7 and 174.1 μM *vs* 8 and 36 μM) alongside similar PAMPA and plasma stability values. The determined parameters for intestinal permeability slightly decreased but remained in desirable ranges (efflux ratio < 1 and *P*_app_ A → B > 10 cm/s), while the metabolic stability measured in microsomes displayed overall comparably poor CL_int_ values (>100 μL/min/mg for both HLM and MLM). Hence, future chemical efforts with respect to said properties might focus on improving the observed limited microsomal stability. However, both of the investigated MKK7 inhibitors displayed a promising *in vitro* pharmacokinetic (*in vitro*-PK) profile (Table 2), considering the experimental state of the compounds that have not yet undergone any optimization, highlighting the suitability of the selected compound scaffolds as well as CuAAC as a synthetic approach for high-throughput LSF.

**Table 2:** Determined *In Vitro*-PK Parameters of **1b** and **1k** in Comparison with **1g** and Ibrutinib^a^

		**1b**	**1k**	**1g**^b^	ibrutinib^b^
kinetic solubility (μM)		97.7	174.1	8	36
permeability [%flux]		36.9	34.3	n.d.	38
CL-(nL/min/mg)	MLM	n.d.	n.d.	124	n.d.
	HLM	256.7	308.1	154	234
plasma stability (%remain)	mouse	118	116	n.d.	98
	human	92	94	70	80
CaCo-2	efflux ratio	0.66	1.03	0.6	0.02
	P- A → B (cm/s)	3.68	11.81	20	40

a MLM: mouse liver microsome; HLM: human liver microsome; *P*_app_: apparent permeability coefficient; n.d.: not detectable.

b Data taken from Wolle et al.^34^

## Conclusions

We demonstrated nanoscale high-throughput late-stage optimization of covalent inhibitors using CuAAC. This approach is amenable to easy miniaturization in plate format and does not require additional purification. The procedure was also carried out in a solvent suitable for downstream biological evaluation, thereby significantly shortening the design–make–test–analyze cycle. Direct biological evaluation of the crude reaction mixtures allowed for the identification of potent compounds in a short time frame and enabled us to focus on a short list of compounds for in-depth profiling.

With regard to the series **1** compounds, the results from ICW highlighted, among others, compounds **1b** and **1k**, which proved to be the two most active inhibitors generated in terms of cellular potency. Co-crystal structures with **1k** and related compounds were able to explain this potency, and while there is still room for improvement in PK, these compounds have attractive features as MKK7 inhibitors and can be further optimized. This pipeline also allowed us to quickly recognize that compounds from series **2** were inferior in terms of potency. Despite our original hypothesis that derivatization from this vector might yield additional favorable interactions, none of these derivatives proved potent, and subsequent co-crystallization studies revealed that indeed the introduced triazolyl moieties resided in solvent-exposed regions in the periphery of the binding pocket.

In conclusion, high-throughput nanomole-scale click reactions are an efficient option to explore the potency of irreversible alkyne-based compounds, derive SAR information, and also improve physicochemical properties. These conditions allow the use of crude reaction mixtures and thus expedite an important stage in the medicinal chemistry process.

## Experimental Section

### Chemistry

#### General Information

Unless otherwise noted, reactions were carried out in air using AR-grade solvents used as received. Chemical building blocks were purchased from Enamine and MolPort chemical suppliers and used as received. Single organic azides were obtained from Enamine, dissolved in DMSO, and stored at −20 °C. Flash chromatography was performed using Merck Silica gel 60 (0.04–0.06 mm) or by CombiFlash Systems (Teledyne Isco) with RediSep Rf Normal-phase Flash Columns. Purification of the final compounds was performed using preparative HPLC; Waters Prep 2545 Preparative Chromatography System, with UV/Vis detector 2489, using XBridge Prep C18 10 μm 10 mm × 250 mm Column (PN: 186003891, SN:161I3608512502). Reaction progress and compounds’ purity were monitored by Waters UPLC-MS system: Acquity UPLC H class with PDA detector, and using Acquity UPLC BEH C18 1.7 μm 2.1 mm × 50 mm Column (PN:186002350, SN 02703533825836). MS system: Waters, SQ detector 2. UPLC Method: 5 min gradient 95:5 Water: Acetonitrile 0.05% formic acid to Acetonitrile 0.05% formic acid, flow rate 0.5 mL/min, column temp 40 °C.

For compounds of series **1**, ^1^H and ^13^C spectra were recorded on a Bruker Avance DRX 400, 500 or 600 spectrometer at 400, 500 or 600 MHz and 101, 125 or 151 MHz, respectively. Chemical shifts are reported in δ (ppm) as s (singlet), d (doublet), dd (doublet of doublet), t (triplet), and m (multiplet) and are referenced to the residual solvent signal: DMSO-*d*_6_ (2.50) for ^1^H and DMSO-*d*_6_ (39.52) for ^13^C. Compound identity was further confirmed by LC-MS analysis on an LCQ Advantage MAX (1200 series, Agilent) with Eclipse XDB-C18-column (5 μM, 150 mm × 1.6 mm, Phenomenex); method: 11 min gradient 5–100% MeCN with 0.05% formic acid at 0.5 mL/min.

For ^1^H and ^13^C NMR spectra of compounds **IB-1-x** and **2a-e**: These were recorded on a Bruker Avance III-300, −400, and −500 MHz spectrometers. Chemical shifts are reported in ppm on the δ scale downfield from TMS and are calibrated according to the deuterated solvents. All J values are given in hertz. For these compounds, high-resolution electron-spray mass spectrometry (HR-MS) was performed on a Xevo G2-XS QTOF Mass Spectrometer (Waters Corporation).

The purity of all final reported compounds was >95%, as determined by HPLC, unless otherwise noted (traces are available in the Supporting Information).

#### General Procedure for Nanoscale CuAAC Using ADE

These reactions were set up using a LabCyte Echo 555 system. Source plate 1 (SP1) contained the azide library (obtained from Enamine, 448 members, 28.57 mM in DMSO) on two 384-well Echo-compatible plates (SP1_1 and SP1_2—SMILES/Enamine codes of the library members are attached as Excel files). Source plate 2 contained alkyne (100 mM in DMSO), sodium ascorbate (freshly prepared, 44 mM in DI water), and CuSO_4_·5 H_2_O/TBTA (1:1 molar ratio, 7.15 mM in 1:1 v/v DMSO/DI water) in separate wells of a 384-well Echo-compatible plate (SP2).

To two 384-well, low-dead-volume Echo-compatible reaction plates (RP_1, RP_2) were first added the azide components from SP1_1 and SP1_2 so that each well contains a single azide (20 nmol, 700 nL, 1.0 equiv). Next, the alkyne (20 nmol, 200 nL, 1.0 equiv), followed by the catalyst mixture (5.0 nmol, 750 nL, 25 mol %), and finally sodium ascorbate (40 nmol, 900 nL, 2.0 equiv) from SP2 were transferred to each well in RP_1 and RP_2 containing an azide, giving a final volume of 2500 nL in each well. The plate was sealed with an aluminum cover, briefly centrifuged, and left to stand for 24 h at room temperature. The expected (theoretical) concentration of the product in each well is 7.97 mM. After 24 h, the reactions were diluted with DMSO to an expected (theoretical) concentration of 3.33 mM.

#### General Procedure **A** for Resynthesis of Triazoles **1a** to **1k**

To a solution of alkyne building block **1** (15 mg, 0.05 mmol, 1 equiv) in H_2_O/*t*-BuOH (1:1), catalytic amounts of Cu(II)SO_4_·5H_2_O (0.007 equiv), sodium ascorbate (0.07 equiv), and corresponding azide (1.1 equiv) were added and stirred for 3 h at room temperature. The reaction mixture was separated between DCM and saturated NaHCO_3_. The combined aqueous layers were additionally extracted with 10% MeOH/DCM, and finally, the combined organic layers were concentrated in vacuo. The described final compounds were purified by silica column chromatography (0–10% MeOH/DCM, yields = 11–42%).

#### General Procedure **B** for the Resynthesis of Triazoles **2a** to **2e**

The alkyne (10–20 μmol, 100 mM in DMSO, stored at −20 °C), the azide (1 equiv, variable concentration in DMSO, stored at −20 °C), and CuSO_4_·5H_2_O/TBTA (12.5 mol %, 1:1 molar ratio of components, 7.15 mM in 1:1 v/v DMSO/H_2_O, stored at −20 °C) were combined in a 1.5 mL Eppendorf vial with stir flea, followed by the addition of sodium ascorbate (2 equiv, neat) under rapid stirring at room temperature. The reaction was tracked by LC-MS, and the reaction mixture was diluted with 50% aq. MeCN (2 mL) upon completion (typically within 1 h), passed through a 0.2 μm syringe tip filter and purified by preparative RP-HPLC.

##### (*R*)-1-(3-(4-Amino-3-(1-(4-bromo-2-hydroxyphenyl)-1*H*-1,2,3-triazol-4-yl)-1*H*-pyrazolo[3,4-*d*]pyrimidin-1-yl)piperidin-1-yl)prop-2-en-1-one (**1a**)

According to the general procedure A, 18 mg (0.06 mmol, 1 equiv) of alkyne building block **1** was reacted. **1a** was obtained after purification by column chromatography in a yield of 5 mg (0.009 mmol, 16%). ^1^H NMR (600 MHz, DMSO-*d*_6_) δ 11.12 (d, *J* = 19.3 Hz, 1H), 9.01 (s, 1H), 8.94 (d, *J* = 10.6 Hz, 1H), 8.23 (d, *J* = 79.9 Hz, 2H), 7.92 (d, *J* = 2.4 Hz, 1H), 7.59 (dd, *J* = 8.8, 2.5 Hz, 1H), 7.14 (d, *J* = 8.8 Hz, 1H), 6.93–6.69 (m, 1H), 6.12 (t, *J* = 17.5 Hz, 1H), 5.66 (dd, *J* = 57.7, 10.4 Hz, 1H), 4.81–4.62 (m, 1H), 4.58 (d, *J* = 11.3 Hz, 0.5H_b_), 4.27–4.11 (m, 1H), 4.09 (d, *J* = 13.2 Hz, 0.5H_b_), 3.81–3.72 (m, 0.5H_a_), 3.21 (dd, *J* = 24.1, 12.2 Hz, 1H), 3.07 (t, *J* = 11.2 Hz, 0.5H_a_), 2.34–2.23 (m, 1H), 2.14 (dd, *J* = 8.9, 3.8 Hz, 1H), 1.99–1.84 (m, 1H), 1.67–1.55 (m, 1H). ^13^C NMR (151 MHz, DMSO-*d*_6_) δ 164.6, 158.3, 156.3, 153.9, 153.8, 149.6, 141.1, 133.4, 128.3, 127.7, 127.4, 125.1, 123.9, 119.1, 109.9, 97.9, 54.9, 53.6 (C_a_), 52.9 (C_b_), 52.4 (C_b_), 49.1 (C_b_), 45.7 (C_a_), 45.2 (C_a_), 41.6 (C_b_), 31.3 (C_b_), 29.5 (C_c_), 29.3 (C_c_), 29.0 (C_c_), 28.7 (C_c_), 28.3 (C_c_), 24.9 (C_d_), 23.1 (C_d_), 22.1 (C_d_).

##### m

LC-MS (ESI-MS): (/*z*) calculated for C_21_H_20_BrN_9_O_2_ ([M + H]^+^): 511.35; found: 510.12. 70% purity estimate based on area under the curve of the chromatogram measured at 280 nm.

##### m

HRMS (ESI-MS): (/*z*) calculated for C_21_H_20_BrN_9_O_2_ ([M + H]^+^): 510.0923; found: 510.0991.

##### (*R*)-1-(3-(4-Amino-3-(1-(2-(4-chlorophenyl)-2,2-difluoroethyl)-1*H*-1,2,3-triazol-4-yl)-1*H*-pyrazolo[3,4-*d*]pyrimidin-1-yl)piperidin-1-yl)prop-2-en-1-one (**1b**)

According to the general procedure A, 15 mg (0.05 mmol, 1 equiv) of alkyne building block **1** was reacted. **1b** was obtained after purification by column chromatography in a yield of 4 mg (0.008 mmol, 16%). ^1^H NMR (600 MHz, DMSO-*d*_6_) δ 8.91 (s, 1H), 8.68 (d, *J* = 13.1, 9.3 Hz, 1H), 8.33–8.19 (m, 1H), 8.11–8.03 (m, 1H), 7.65 (s, 4H), 6.79 (ddd, 1H), 6.09 (dd, *J* = 30.3, 15.3 Hz, 1H), 5.65 (dd, *J* = 69.9, 10.4 Hz, 1H), 5.48 (t, ^3^*J*(H,F) = 14.3 Hz, 2H), 4.79–4.63 (m, 1H), 4.55 (d, *J* = 11.2 Hz, 0.5H_a_), 4.23–4.14 (m, 1H), 4.09 (d, 0.5H_a_), 3.77–3.68 (m, 0.5H_b_), 3.24–3.15 (m, 1H), 3.07 (t, *J* = 11.2 Hz, 0.5H_b_), 2.31–2.20 (m, *J* = 6.7 Hz, 1H), 2.19–2.09 (m, *J* = 24.0, 8.0 Hz, 1H), 2.00–1.88 (m, *J* = 28.4 Hz, 1H), 1.65–1.54 (m, *J* = 29.4, 19.8 Hz, 1H). ^13^C NMR (151 MHz, DMSO-*d*_6_) δ 164.6, 158.3, 156.8, 156.4, 153.9, 141.3, 136.0, 134.8, 132.0, 129.1 (2C), 128.3, 127.4, 124.3, 119.7 (d, ^1^*J*(C,F) = 245.8 Hz), 117.3, 97.6, 69.8, 54.2, 52.8 (C_a_), 52.3 (C_a_), 49.1 (C_b_), 45.7 (C_a_), 45.2 (C_a_), 41.6 (C_b_), 31.3 (C_b_), 29.5 (C_c_), 29.0 (C_c_), 28.9 (C_c_), 28.7 (C_c_), 25.9 (C_d_), 24.9 (C_d_), 23.1 (C_d_), 22.1 (C_d_).

##### m

LC-MS (ESI-MS): (/*z*) calculated for C_23_H_22_ClF_2_N_9_O ([M + H]^+^): 514.39; found: 514.21; > 95% purity was estimated based on area under the curve of the chromatogram measured at 280 nm.

##### m

HRMS (ESI-MS): (/*z*) calculated for C_23_H_22_ClF_2_N_9_O ([M + H]^+^): 514.1604; found: 514.1671.

##### (*R*)-1-(3-(4-Amino-3-(1-(3-fluoro-4-(hydroxymethyl)phenyl)-1*H*-1,2,3-triazol-4-yl)-1*H*-pyrazolo[3,4-*d*]pyrimidin-1-yl)piperidin-1-yl)prop-2-en-1-one (**1c**)

According to the general procedure A, 18 mg (0.06 mmol, 1 equiv) of alkyne building block **1** was reacted. **1c** was obtained after purification by column chromatography in a yield of 5 mg (0.01 mmol, 22%). ^1^H NMR (600 MHz, DMSO-*d*_6_) δ 9.39 (s, 1H), 8.96 (s, 1H), 8.23 (d, *J* = 83.2 Hz, 2H), 8.00–7.87 (m, 2H), 7.70 (t, *J* = 8.1 Hz, 1H), 6.82 (ddd, *J* = 65.6, 16.2, 10.7 Hz, 1H), 6.17–6.06 (m, 1H), 5.67 (dd, *J* = 46.8, 10.1 Hz, 1H), 5.45 (t, *J* = 5.6 Hz, 1H), 4.77–4.66 (m, 1H), 4.65–4.58 (m, 2H+0.5H_a_), 4.31 (dd, *J* = 34.9, 11.9 Hz, 1H), 4.12 (d, *J* = 12.7 Hz, 0.5H_a_), 3.69–3.59 (m, 0.5H_b_), 3.16 (dd, *J* = 23.7, 11.9 Hz, 1H), 2.96–2.83 (m, 0.5H_b_), 2.30 (ddd, *J* = 15.9, 12.5, 4.1 Hz, 1H), 2.20–2.11 (m, 1H), 1.99–1.91 (m, 1H), 1.67–1.55 (m, 1H). ^13^C NMR (151 MHz, DMSO-*d*_6_) δ 164.6, 159.5 (d, ^1^*J*(C,F) = 245.8 Hz), 158.3, 156.3, 153.9, 142.1, 135.9, 134.8, 130.2, 130.1, 128.2, 127.5, 120.7, 116.0, 107.7, 98.0, 56.4, 56.4, 53.2 (C_a_), 52.5 (C_a_), 49.2 (C_b_), 45.7 (C_a_), 45.3 (C_a_), 41.7 (C_b_), 31.3 (C_b_), 29.5 (C_c_), 29.4 (C_c_), 29.0 (C_c_), 28.7 (C_c_), 28.2 (C_c_), 24.9 (C_d_), 23.4 (C_d_), 22.1 (C_d_), 13.9 (C_d_).

##### m

LC-MS (ESI-MS): (/*z*) calculated for C_22_H_22_FN_9_O_2_ ([M + H]^+^): 464.47; found: 464.21. 90% purity estimate based on area under the curve of the chromatogram measured at 280 nm.

##### m

HRMS (ESI-MS): (/*z*) calculated for C_22_H_22_FN_9_O_2_ ([M + H]^+^): 464.1880; found: 464.1948.

##### (*R*)-1-(3-(4-Amino-3-(1-(4-chloro-2-hydroxyphenyl)-1*H*-1,2,3-triazol-4-yl)-1*H*-pyrazolo[3,4-*d*]pyrimidin-1-yl)piperidin-1-yl)prop-2-en-1-one (**1d**)

According to the general procedure A, 15 mg (0.05 mmol, 1 equiv) of alkyne building block **1** was reacted. **1d** was obtained after purification by column chromatography in a yield of 4 mg (0.009 mmol, 17%). ^1^H NMR (600 MHz, DMSO-*d*_6_) δ 11.33 (s, 1H), 8.97 (d, *J* = 54.1 Hz, 2H), 8.21 (d, *J* = 80.3 Hz, 2H), 7.75 (s, 1H), 7.18 (t, *J* = 39.9 Hz, 2H), 6.96–6.68 (m, 1H), 6.11 (t, *J* = 18.2 Hz, 1H), 5.66 (dd, *J* = 59.8, 10.2 Hz, 1H), 4.71 (d, *J* = 31.4 Hz, 1H), 4.57 (d, *J* = 10.9 Hz, 0.5H_a_), 4.19 (t, *J* = 14.9 Hz, 1H), 4.09 (d, *J* = 13.5 Hz, 0.5H_a_), 3.80–3.69 (m, 0.5H_b_), 3.20 (dd, *J* = 23.1, 11.6 Hz, 1H), 3.13–3.02 (m, 0.5H_b_), 2.33–2.22 (m, 1H), 2.18–2.09 (m, 1H), 1.98–1.87 (m, 1H), 1.66–1.52 (m, 1H). ^13^C NMR (151 MHz, DMSO-*d*_6_) δ 164.6, 158.3, 156.9, 156.4, 153.8, 151.1, 141.1, 134.6, 128.3, 127.5, 127.0, 124.0, 123.3, 119.6, 116.8, 97.8, 56.0, 52.9 (C_a_), 52.4 (C_a_), 49.1 (C_b_), 45.5 (C_a_), 45.2 (C_a_), 41.6 (C_b_), 32.3 (C_b_), 31.3 (C_b_), 30.4 (C_b_), 29.5 (C_c_), 29.4 (C_c_), 29.0 (C_c_), 28.7 (C_c_), 25.9 (C_d_), 24.9 (C_d_), 23.1 (C_d_), 22.1 (C_d_).

##### m

LC-MS (ESI-MS): (/*z*) calculated for C_21_H_20_ClN_9_O_2_ ([M + H]^+^): 466.90; found: 466.15; > 95% purity was estimated based on area under the curve of the chromatogram measured at 280 nm.

##### m

HRMS (ESI-MS): (/*z*) calculated for C_21_H_20_ClN_9_O_2_ ([M + H]^+^): 466.1428; found: 466.1499.

##### 1-((*R*)-3-(4-Amino-3-(1-((*R*)-2-(4-bromophenyl)-2-hydroxy-ethyl)-1*H*-1,2,3-triazol-4-yl)-1*H*-pyrazolo[3,4-*d*]pyrimidin-1-yl)piperidin-1-yl)prop-2-en-1-one (**1e**)

According to the general procedure A, 15 mg (0.05 mmol, 1 equiv) of alkyne building block **1** was reacted. **1e** was obtained after purification by column chromatography in a yield of 3 mg (0.006 mmol, 11%). ^1^H NMR (600 MHz, DMSO-*d*_6_) δ 9.13 (s, 1H), 8.63 (d, *J* = 10.0 Hz, 1H), 8.25 (s, 1H), 8.09 (s, 1H), 7.57 (d, *J* = 8.4 Hz, 2H), 7.39 (d, *J* = 8.4 Hz, 2H), 6.94–6.65 (m, 1H), 6.19–6.04 (m, 1H), 6.00 (d, *J* = 4.6 Hz, 1H), 5.67 (dd, *J* = 62.8, 10.2 Hz, 1H), 5.17–5.07 (m, 1H), 4.78–4.63 (m, 2H), 4.63–4.50 (m, 1 + 0.5H_a_), 4.33–4.18 (m, 1H), 4.11 (d, *J* = 12.8 Hz, 0.5H_a_), 3.72–3.66 (m, 0.5H_b_), 3.24–3.07 (m, 1H), 3.00 (t, *J* = 11.0 Hz, 0.5H_b_), 2.34–2.20 (m, 1H), 2.19–2.05 (m, 1H), 2.04–1.85 (m, 1H), 1.74–1.52 (m, 1H). ^13^C NMR (151 MHz, DMSO-*d*_6_) δ 164.6, 158.3, 156.3, 153.8, 141.2, 140.8, 135.4, 131.2 (2C), 128.4 (2C), 128.2, 127.6, 123.3, 120.7, 97.6, 70.5, 56.7, 52.9 (C_a_), 52.2 (C_a_), 49.2 (C_b_), 45.7 (C_a_), 45.2 (C_a_), 41.6 (C_b_), 31.3 (C_b_), 29.6 (C_c_), 29.4 (C_c_), 29.0 (C_c_), 28.7 (C_c_), 24.9 (C_d_), 23.3 (C_d_), 22.1 (C_d_).

##### m

LC-MS (ESI-MS): (/*z*) calculated for C_23_H_24_BrN_9_O_2_ ([M + H]^+^): 539.41; found: 538.15. 75% purity estimate based on area under the curve of the chromatogram measured at 280 nm.

##### m

HRMS (ESI-MS): (/*z*) calculated for C_23_H_24_BrN_9_O_2_ ([M + H]^+^): 538.1236; found: 538.1307.

##### (*R*)-1-(3-(4-Amino-3-(1-(4-bromophenyl)-1*H*-1,2,3-triazol-4-yl)-1*H*-pyrazolo[3,4-*d*]pyrimidin-1-yl)piperidin-1-yl)prop-2-en-1-one (**1f**)

According to the general procedure A, 15 mg (0.05 mmol, 1 equiv) of alkyne building block **1** was reacted. **1f** was obtained after purification by column chromatography in a yield of 3 mg (0.006 mmol, 12%). ^1^H NMR (600 MHz, DMSO-*d*_6_) δ 9.36 (d, *J* = 7.0 Hz, 1H), 8.96 (s, 1H), 8.24 (d, *J* = 81.3 Hz, 2H), 8.03 (d, *J* = 8.6 Hz, 2H), 7.82 (d, *J* = 8.5 Hz, 2H), 6.92–6.69 (m, 1H), 6.20–6.01 (m, 1H), 5.68 (dd, *J* = 47.7, 10.1 Hz, 1H), 4.79–4.66 (m, 1H), 4.64 (d, *J* = 11.6 Hz, 0.5H_a_), 4.31 (dd, *J* = 33.0, 11.9 Hz, 1H), 4.13 (d, *J* = 12.6 Hz, 0.5H_a_), 3.67 (t, *J* = 11.5 Hz, 0.5H_b_), 3.16 (dd, *J* = 25.2, 12.8 Hz, 1H), 2.96–2.80 (m, 0.5H_b_), 2.40–2.23 (m, 1H), 2.20–2.10 (m, 1H), 2.05–1.77 (m, 1H), 1.70–1.54 (m, 1H). ^13^C NMR (151 MHz, DMSO-*d*_6_) δ 164.6, 158.3, 156.3, 153.8, 142.2, 135.4, 134.7, 132.7 (2C), 128.2 (d, *J* = 28.1 Hz), 127.5, 122.4 (2C), 122.0, 120.6, 98.1, 54.9, 52.5 (C_a_), 51.5 (C_b_), 49.2 (C_b_), 45.7 (C_a_), 45.2 (C_a_), 41.7 (C_b_), 31.3 (C_b_), 29.5 (C_c_), 29.4 (C_c_), 29.0 (C_c_), 28.7 (C_c_), 24.9 (C_d_), 23.4 (C_d_).

##### m

LC-MS (ESI-MS): (/*z*) calculated for C_21_H_20_BrN_9_O ([M + H]^+^): 495.35; found: 494.08. 94% purity estimate based on area under the curve of the chromatogram measured at 280 nm.

##### m

HRMS (ESI-MS): (/*z*) calculated for C_21_H_20_BrN_9_O ([M + H]^+^): 494.0974 found: 494.1042.

##### (*R*)-1-(3-(4-Amino-3-(1-phenethyl-1*H*-1,2,3-triazol-4-yl)-1*H*-pyrazolo[3,4-*d*]pyrimidin-1-yl)piperidin-1-yl)prop-2-en-1-one (**1h**)

According to the general procedure A, 15 mg (0.05 mmol, 1 equiv) of alkyne building block **1** was reacted. **1h** was obtained after purification by column chromatography in a yield of 7 mg (0.02 mmol, 32%). ^1^H NMR (600 MHz, DMSO-*d*_6_) δ 9.11 (s, 1H), 8.66 (d, *J* = 12.5 Hz, 1H), 8.22 (s, 1H), 8.07 (s, 1H), 7.31–7.25 (m, 2H), 7.25–7.16 (m, 3H), 6.79 (ddd, *J* = 83.6, 16.4, 10.6 Hz, 1H), 6.10 (dd, *J* = 32.4, 16.8 Hz, 1H), 5.65 (dd, *J* = 64.4, 10.3 Hz, 1H), 4.74 (t, *J* = 7.3 Hz, 2H), 4.71–4.60 (m, 1H), 4.56 (d, *J* = 11.3 Hz, 0.5H_a_), 4.29–4.16 (m, 1H), 4.09 (d, *J* = 13.2 Hz, 0.5H_a_), 3.71–3.60 (m, 0.5H_b_), 3.26 (t, *J* = 7.3 Hz, 2H), 3.20–3.10 (m, 1H), 2.96 (t, *J* = 11.0 Hz, 0.5H_b_), 2.28–2.17 (m, 1H), 2.15–2.05 (m, 1H), 1.97–1.85 (m, 1H), 1.62–1.51 (m, 1H). ^13^C NMR (151 MHz, DMSO-*d*_6_) δ 174.8, 164.7, 158.4, 156.4, 153.9, 141.0, 137.5, 128.8 (2C), 128.5 (2C), 128.3, 127.6, 126.7, 122.7, 97.6, 53.0 (C_a_), 52.3 (C_a_), 51.0, 49.2 (C_b_), 45.8 (C_a_), 45.3 (C_a_), 41.7 (C_b_), 35.5, 29.6 (C_c_), 29.4 (C_c_), 25.3 (C_d_), 24.9 (C_d_), 23.4 (C_d_).

##### m

LC-MS (ESI-MS): (/*z*) calculated for C_23_H_22_ClF_2_N_9_O ([M + H]^+^): 444.51; found: 444.25; > 95% purity was estimated based on area under the curve of the chromatogram measured at 280 nm.

##### m

HRMS (ESI-MS): (/*z*) calculated for C_23_H_22_ClF_2_N_9_O ([M + H]^+^): 444.2182; found: 444.2252.

##### (*R*)-1-(3-(4-Amino-3-(1-(4-methoxyphenyl)-1*H*-1,2,3-triazol-4-yl)-1*H*-pyrazolo[3,4-*d*]pyrimidin-1-yl)piperidin-1-yl)prop-2-en-1-one (**1i**)

According to the general procedure A, 15 mg (0.05 mmol, 1 equiv) of alkyne building block **1** was reacted. **1i** was obtained in a yield of 6 mg (0.01 mmol, 27%) after purification by column chromatography. ^1^H NMR (600 MHz, DMSO-*d*_6_) δ 9.23 (d, *J* = 10.0 Hz, 1H), 9.08 (s, *J* = 31.1 Hz, 1H), 8.23 (d, *J* = 90.3 Hz, 2H), 7.96 (d, *J* = 8.8 Hz, 2H), 7.16 (d, *J* = 8.8 Hz, 2H), 6.92–6.69 (m, 1H), 6.17–6.00 (m, 1H), 5.67 (dd, *J* = 47.6, 10.0 Hz, 1H), 4.79–4.65 (m, 1H), 4.62 (d, *J* = 11.0 Hz, 0.5H_a_), 4.29 (dd, *J* = 32.1, 12.1 Hz, 1H), 4.12 (d, *J* = 12.5 Hz, 0.5H_a_), 3.84 (s, 3H), 3.72–3.64 (m, 0.5H_b_), 3.25–3.08 (m, 1H), 2.97–2.83 (m, 0.5H_b_), 2.39–2.22 (m, 1H), 2.20–2.09 (m, 1H), 2.01–1.84 (m, 1H), 1.74–1.50 (m, 1H). ^13^C NMR (151 MHz, DMSO-*d*_6_) δ 164.6, 159.7, 158.3, 156.3, 153.8, 141.9, 135.1, 129.6, 128.2, 127.4, 122.1, 120.5, 114.9, 98.1, 55.6, 54.9 (C_b_), 53.2 (C_a_), 52.5 (C_a_), 51.5 (C_b_), 49.2 (C_b_), 45.7 (C_a_), 45.2 (C_a_), 41.7 (C_b_), 31.3 (C_b_), 31.1 (C_b_), 29.5 (C_c_), 29.4 (C_c_), 29.0 (C_c_), 28.7 (C_c_), 28.2 (C_c_), 24.9 (C_d_), 23.4 (C_d_), 22.1 (C_d_).

##### m

LC-MS (ESI-MS): (/*z*) calculated for C_22_H_23_N_9_O_2_ ([M + H]^+^): 446.48; found: 446.19. 89% purity estimate based on area under the curve of the chromatogram measured at 280 nm.

##### m

HRMS (ESI-MS): (/*z*) calculated for C_22_H_23_N_9_O_2_ ([M + H]^+^): 446.1975; found: 446.2047.

##### Methyl (*R*)-4-(4-(1-(1-Acryloylpiperidin-3-yl)-4-amino-1*H*-pyrazolo [3,4-*d*]pyrimidin-3-yl)-1*H*-1,2,3-triazol-1-yl)benzoate (**1j**)

According to the general procedure A, 15 mg (0.05 mmol, 1 equiv) of alkyne building block **1** was reacted. **1j** was obtained after purification by column chromatography in a yield of 10 mg (0.02 mmol, 42%). ^1^H NMR (600 MHz, DMSO-*d*_6_) δ 9.52 (s, 1H), 8.93 (s, 1H), 8.30–8.25 (m, 2H), 8.22–8.17 (m, 2H), 6.92–6.72 (m, 1H), 6.18–6.06 (m, 1H), 5.68 (dd, *J* = 51.7, 10.1 Hz, 1H), 4.77–4.67 (m, *J* = 10.0 Hz, 1H), 4.62 (d, *J* = 10.8 Hz, 0.5H_a_), 4.31 (dd, *J* = 30.1, 13.6 Hz, 1H), 4.13 (d, *J* = 14.4 Hz, 0.5H_a_), 3.91 (s, 3H), 3.71–3.65 (m, 0.5H_b_), 3.22–3.12 (m, 1H), 2.91 (t, *J* = 11.4 Hz, 0.5H_b_), 2.34–2.25 (m, 1H), 2.20–2.13 (m, *J* = 8.7, 4.1 Hz, 1H), 2.00–1.92 (m, *J* = 9.5 Hz, 1H), 1.68–1.56 (m, 1H). ^13^C NMR (151 MHz, DMSO-*d*_6_) δ 165.2, 164.6, 158.2, 156.3, 153.8, 142.3, 139.3, 134.6, 130.8, 129.8, 128.2, 127.5, 120.6, 120.2, 97.9, 54.9, 53.2 (C_a_), 52.4, 49.2 (C_b_), 45.7 (C_a_), 45.3 (C_a_), 41.7 (C_b_), 29.5 (C_c_), 29.4 (C_c_), 29.0 (C_c_), 24.9 (C_d_), 23.4 (C_d_), 22.1 (C_d_).

##### m

LC-MS (ESI-MS): (/*z*) calculated for C_23_H_23_N_9_O_3_ ([M + H]^+^): 474.49; found: 474.16. 90% purity estimate based on area under the curve of the chromatogram measured at 280 nm.

##### m

HRMS (ESI-MS): (/*z*) calculated for C_23_H_23_N_9_O_3_ ([M + H]^+^): 474.1924; found: 474.1993.

##### (*R*)-1-(3-(4-Amino-3-(1-(2,2-difluoro-2-phenylethyl)-1*H*-1,2,3-triazol-4-yl)-1*H*-pyrazolo[3,4-*d*]pyrimidin-1-yl)piperidin-1-yl)prop-2-en-1-one (**1k**)

According to the general procedure A, 15 mg (0.05 mmol, 1 equiv) of alkyne building block **1** was reacted. **1k** was obtained after purification by column chromatography in a yield of 4 mg (0.008 mmol, 17%). ^1^H NMR (600 MHz, DMSO-*d*_6_) δ 8.95 (s, 1H), 8.68 (d, *J* = 13.6 Hz, 1H), 8.25 (s, 1H), 8.12 (s, 1H), 7.66–7.61 (m, 2H), 7.60–7.54 (m, 3H), 6.80 (ddd, *J* = 85.2, 16.4, 10.5 Hz, 1H), 6.11 (dd, *J* = 27.0, 16.7 Hz, 1H), 5.66 (dd, *J* = 68.3, 10.4 Hz, 1H), 5.47 (t, ^3^*J*(H,F) = 14.6 Hz, 2H), 4.80–4.62 (m, 1H), 4.56 (d, *J* = 11.0 Hz, 0.5H_a_), 4.20 (t, *J* = 9.6 Hz, 1H), 4.09 (d, *J* = 13.2 Hz, 0.5H_a_), 3.77–3.69 (m, 0.5H_b_), 3.21 (t, *J* = 11.7 Hz, 1H), 3.07 (t, *J* = 11.1 Hz, 0.5H_b_), 2.31–2.20 (m, 1H), 2.17–2.10 (m, 1H), 1.99–1.88 (m, 1H), 1.63–1.51 (m, 1H). ^13^C NMR (151 MHz, DMSO-*d*_6_) δ 174.6, 164.6, 158.3, 157.8, 156.4, 153.8, 141.2, 134.9, 133.1, 131.1, 128.9 (2C), 128.0, 125.3, 124.3, 120.0 (d, ^1^*J*(C,F) = 245.2 Hz), 116.4, 97.6, 54.4, 52.8, 52.3 (C_a_), 49.1 (C_b_), 45.7 (C_a_), 45.2 (C_a_), 41.6 (C_b_), 29.5 (C_c_), 29.3 (C_c_), 25.0 (C_d_), 24.9 (C_d_), 23.2 (C_d_).

##### m

LC-MS (ESI-MS): (/*z*) calculated for C_23_H_23_F_2_N_9_O ([M + H]^+^): 480.49; found: 480.22. 72% purity estimate based on area under the curve of the chromatogram measured at 280 nm.

##### m

HRMS (ESI-MS): (/*z*) calculated for C_23_H_23_F_2_N_9_O ([M + H]^+^): 480.1994; found: 480.2061.

#### Synthesis of IB-1-24 (**2**) (See Overall Scheme in the Supporting Information)

Reaction conditions: (a) Dihydropyran, *p*TSOH, EtOAc overnight, 90 °C; (b) 3-methoxycarbonyl-5-nitrophenylboronic acid, PdCl_2_(dppf), K_2_CO_3_, dioxane/H_2_O (2.5:1), 1 h, 50 °C; (c) H_2_, Pd/C, EtOAc 2.5 h, RT; (d) acrylic acid, DMAP, EDC, DCM, 2.5h, 0 °C; (e) LiOH, THF/H_2_O (3:1), 4 h, RT; (f) propargylamine, DMAP, EDC, DCM/DMF (1:0.1), overnight, RT; (g) HCl, MeOH, 2 h, 0 °C.

##### 3-Iodo-1-(tetrahydro-2*H*-pyran-2-yl)-1*H*-indazole (**IB-1-18**)

To a solution of 3-iodoindazole (1 equiv, 1.0089 g) in ethyl acetate (10 mL), DHP (2 equiv, 700 μL) and *p*TsOH (0.1 equiv, 81.4 mg) were added. The reaction was stirred in a 150 mL bomb reactor. The mixture was heated to 90 °C overnight. The completion of the reaction was monitored by LC-MS. The crude mixture was diluted with 50 mL of EtOAc; washed once with water, NaHCO_3_, and brine; and dried with Na_2_SO_4_. It was filtered and concentrated under reduced pressure. The crude was purified by flash column chromatography (24 g prepacked SiO2 column, mobile phase 0 to 100% EtOAc in hexane in 20 min). The fractions with pure product were combined and concentrated. The desired intermediate **IB-1-18** was isolated in 80% yield.

^1^H NMR (400 MHz, DMSO-*d*_6_) δ ppm 7.76 (d, *J* = 8 Hz, 1H), 7.52 (dd, *J* = 4 Hz, 1H), 7.45 (d, *J* = 8.12 Hz, 1H), 7.28 (dd, *J* = 7.4 Hz, 1H), 5.86 (dd, *J* = 9.84, 2.4 Hz, 1H), 3.83–3.93 (m, 1H), 3.68–3.79 (m, 1H), 2.31–2.45 (m, 1H), 1.93–2.09 (m, 2H), 1.66–1.82 (m, 1H), 1.51–1.66 (m, 2H). ^13^C NMR (100 MHz, DMSO) δ ppm 139.78, 128.07, 127.59, 122.06, 120.83, 110.63, 94.47, 84.01, 66.59, 28.82, 24.63, 22.12.

##### m

LC-MS (ESI-MS): (/*z*) calculated for C_12_H_14_IN_2_O ([M + H]^+^) 329.0, found 329.2.

##### Methyl 3-Nitro-5-(1-(tetrahydro-2*H*-pyran-2-yl)-1*H*-indazol-3-yl)benzoate (**IB-1-19**)

**IB-1-18** (1.0883 g, 1 equiv) was dissolved in 10 mL of 1,4-dioxane in a 20 mL MW tube. To the tube, PdCl_2_(dppf) (121.1 mg, 5%) and (3-(methoxycarbonyl)-5-nitrophenyl)boronic acid (817.4 mg, 1.1 equiv) were added. Potassium carbonate (1.365 g, 3 equiv) and 4 mL of water were added. The tube was sealed. The atmosphere inside the vial was replaced with argon after flushing it three times: vacuum–argon. The reaction mixture was irradiated and stirred in a microwave at 50 °C for 1 h.

The crude was analyzed by LC-MS and showed ∼80% conversion. The crude was diluted with EtOAc and washed with water. The water layer was extracted three times with ethyl acetate. The combined organics were washed with saturated sodium bicarbonate solution and brine, dried over sodium sulfate, and concentrated under reduced pressure before purification by flash chromatography (prepacked SiO_2_ cartridge, mobile phase: gradient of 0–80% strong solvent in hexane, for 20 min, the strong solvent was EtOAc, then for 5 min, MeOH, total gradient 5 min). The target fractions with ∼95% pure compound were combined and concentrated. **IB-1-19** was obtained in ∼80% yield (1.006 g).

^1^H NMR (400 MHz, DMSO-*d*_6_) δ ppm 8.87 (s, 1H), 8.82 (s, 1H), 8.59 (s, 1H), 8.08 (d, *J* = 8 Hz, 1H), 7.87 (d, *J* = 8.4 Hz, 1H), 7.53 (dd, *J* = 7.2 Hz, 1H), 7.37 (dd, *J* = 7.6 Hz, 1H), 5.98 (dd, *J* = 9.32, 1.76 Hz, 1H), 3.96 (s, 3H), 3.86–3.94 (m, 1H), 3.72–3.83 (m, 1H), 1.97–2.13 (m, 2H), 1.70–1.85 (m, 2H), 1.53–1.69 (m, 2H). ^13^C NMR (100 MHz, DMSO) δ ppm 164.29, 148.55, 140.89, 139.71, 135.14, 132.15, 131.88, 127.01, 124.79, 122.89, 122.46, 120.84, 120.04, 111.25, 84.17, 66.66, 52.95, 28.83, 24.59, 22. 03.

##### m

LC-MS (ESI-MS): (/*z*) calculated for C_20_H_20_N_3_O_5_ ([M + H]^+^) 382.1, found 382.3.

##### Methyl 3-Amino-5-(1-(tetrahydro-2*H*-pyran-2-yl)-1*H*-indazol-3-yl)benzoate (**IB-1-20**)

**IB-1-19** (19 mg to 1 equiv) was dissolved in 2 mL of EtOAc, then heated slightly to dissolve all material, and then cooled to r.t. Pd/C (10%) was added to the flask. Then, H_2_ was bubbled into solution using a needle for 30 min with an exhaust needle and removed and stirred for 2 h in H_2_ atm. The reaction was monitored using LC-MS, and it was completed after 2 h. The crude was filtered through Celite. The solvent was evaporated, and **IB-1-20** was obtained in 100% yield.

^1^H NMR (300 MHz, MeOD) δ ppm 7.95 (d, *J* = 8.16 Hz, 1H), 7.87 (s, 1H), 7.62 (d, *J* = 8.46 Hz, 1H), 7.51 (s, 1H), 7.28–7.46 (m, 2H), 7.19 (dd, *J* = 7.56 Hz, 1H), 5.75 (dd, *J* = 7.8, 1.5 Hz, 1H), 3.91–4.01 (m, 1H), 3.87 (s, 3H), 3.66–3.81 (m, 1H), 2.42–2.68 (m, 2H), 1.88–2.17 (m, 2H), 1.49–1.84 (m, 2H). ^13^C NMR (75 MHz, MeOD) δ ppm 168.96, 150.01, 145.13, 142.50, 135.79, 132.55, 127.85, 123.35, 123.04, 122.11, 119.32, 118.85, 116.43, 111.63, 86.42, 68.63, 52.68, 30.57, 26.33, 23.76.

##### m

LC-MS (ESI-MS): (/*z*) calculated for ([M + H]^+^) C_20_H_22_N_3_O_3_, 352.2, found 352.3.

##### Methyl 3-Acrylamido-5-(1-(tetrahydro-2*H*-pyran-2-yl)-1*H*-indazol-3-yl)benzoate (**IB-1-21**)

**IB-1-20** (717.3 mg, 1 equiv) was dissolved in 15 mL of DCM. To a different flask: 420 μL (−3 equiv) of acrylic acid was added to 15 mL of DCM and then cooled to 0 °C, followed by the addition of 26.8 mg (0.1 equiv) of DMAP. Then, EDC (hydrochloride) was added (1.2851 g, 3.1 equiv). The solution was stirred at 0 °C for 35 min. Then, a solution of **IB-1-20** was added and stirred at 0 °C for 2 h. When the reaction ended, it was quenched with 15 mL of MeOH and concentrated under reducing pressure to give crude **IB-1-21**, which was purified by flash chromatography (column of 12 g) with EtOAc in DCM-increasing gradient until 100%. **IB-1-21** eluted in 15–50% EA. The fractions with pure product were combined, evaporated, and then dried to obtain 501.8 mg (56% yield) in 95% purity.

^1^H NMR (400 MHz, CDCl_3_) δ ppm 8.54 (s, 1H), 8.39 (s, 1H), 8.18 (s, 1H), 8.04 (d, *J* = 8.16 Hz, 1H), 7.59 (d, *J* = 8.44 Hz, 1H), 7.39 (dd, *J* = 7.36 Hz, 1H), 7.21 (dd, *J* = 7.6 Hz, 1H), 6.43 (dd, *J* = 16, 0.96 Hz, 1H), 6.21–6.34 (m, 1H), 5.75 (dd, *J* = 8, 4 Hz, 1H), 5.71 (dd, *J* = 7.36, 0.88 Hz, 1H), 4.01–4.11 (m, 1H), 3.90 (s, 3H), 3.69–3.82 (m, 1H), 2.56–2.74 (m, 2H), 2.03–2.24 (m, 2H), 1.50–1.84 (m, 2H). ^13^C NMR (100 MHz, CDCl_3_) δ ppm 166.88, 164.11, 143.16, 141.14, 138.66, 134.83, 131.36, 131.09, 128.24, 126.80, 124.48, 123.33, 122.40, 122.26, 121.35, 120.39, 110.46, 85.66, 67.77, 52.45, 29.56, 25.26, 22.78.

##### m

LC-MS (ESI-MS): (/*z*) calculated for ([M + H]^+^) C_23_H_24_N_3_O_4_, 406.2, found 406.4.

##### 3-Acrylamido-5-(1-(tetrahydro-2*H*-pyran-2-yl)-1*H*-indazol-3-yl)benzoic acid (**IB-1-22**)

**IB-1-21** (270.5 mg) was dissolved in 9 mL of THF. LiOH (anhydrous) (120.8 mg) was dissolved in 3 mL of water. The base solution was added to **IB-1-21**. The reaction was stirred for 4 h at r.t. under Ar atmosphere.

The crude mixture was acidified with HCl 37% until pH 1. Then, extractions were made with ethyl acetate three times. The organics were washed once with brine and dried with sodium sulfate. The filtrate evaporated, and the product was purified in combi-flash twice with EA in DCM, starting from 45 to 100%, for 20 min. Fractions with pure product were combined and evaporated. **IB-1-22** was obtained in 97% yield.

^1^H NMR (400 MHz, DMSO-*d*_6_) δ ppm 10.41 (s, 1H), 8.58 (s, 1H), 8.24 (s, 1H), 8.21 (s, 1H), 8.08 (d, *J* = 8.2 Hz, 1H), 7.82 (d, *J* = 8.52 Hz, 1H), 7.49 (dd, *J* = 7.32 Hz, 1H), 7.33 (dd, *J* = 7.6 Hz, 1H), 6.42–6.56 (m, 1H), 6.32 (dd, *J* = 15.52, 2.12 Hz, 1H), 5.94 (dd, *J* = 8.92, 2.36 Hz, 1H), 5.79 (dd, *J* = 10.52, 1.84 Hz, 1H), 3.88–3.97 (m, 1H), 3.73–3.83 (m, 1H), 1.92–2.14 (m, 2H), 1.72–1.89 (m, 2H), 1.55–1.69 (m, 2H). ^13^C NMR (100 MHz, MeOD) δ ppm 169.19, 166.34, 144.08, 142.56, 140.54, 135.73, 133.21, 132.25, 128.25, 127.84, 125.01, 123.89, 123.25, 123.18, 121.84, 121.41, 111.70, 86.33, 68.44, 30.44, 26.26, 23.59.

##### m

LC-MS (ESI-MS): (/*z*) calculated for C_22_H_22_N_3_O_4_ ([M + H]^+^) 392.2, found 392.3

##### 3-Acrylamido-*N*-(prop-2-yn-1-yl)-5-(1-(tetrahydro-2*H*-pyran-2-yl)-1*H*-indazol-3-yl)benzamide (**IB-1-23**)

**IB-1-22** (110.6 mg) was dissolved in 4 mL of DCM, and 0.5 mL of DMF was added to full dissolution. It was cooled in an ice bath. EDC was added (63.7 mg to 1.1 equiv), and then DMAP was added (3 mg to 0.1 equiv); after 20 min, prop-2-yn-1-amine (27 μL) was added (dissolved in 1 mL of DCM). The reaction mixture was stirred overnight. The reaction crude was purified by flash column chromatography with increasing gradient of EA in DCM (0–100%). The fractions with pure product were combined, evaporated, and dried in high vacuum. **IB-1-23** (95.6 mg) was obtained (79% yield).

^1^H NMR (300 MHz, DMSO-*d*_6_) δ ppm 10.491 (s, 1H), 9.14 (t, *J* = 4.89, 1H), 8.22 (s, 1H), 8.04–8.23 (m, 3H), 7.83 (d, *J* = 8.43 Hz, 1H), 7.50 (dd, *J* = 7.14 Hz, 1H), 7.33 (dd, *J* = 7.56 Hz, 1H), 6.40–6.55 (m, 1H), 6.24–6.39 (m, 1H), 5.88–6.01 (m, 1H), 5.73–5.87 (m, 1H), 4.01–4.15 (m, 2H), 3.87–3.99 (m, 1H), 3.69–3.85 (m, 1H), 1.22 (s, 1H), 1.91–2.18 (m, 2H), 1.71–1.90 (m, 2H), 1.51–1.69 (m, 2H). ^13^C NMR (75 MHz, DMSO-*d*_6_) δ ppm 165.79, 163.43, 142.12, 140.84, 139.62, 135.42, 133.60, 131.62, 127.40, 126.65, 122.07, 121.18, 120.77, 120.31, 120.14, 118.12, 110.86, 84.05, 81.27, 72.81, 66.70, 28.95, 28.58, 24.71, 22.21.

##### m

LC-MS (ESI-MS): (/*z*) calculated for C_25_H_25_N_4_O_3_ ([M + H]^+^) 429.2, found 429.4.

##### 3-Acrylamido-5-(1*H*-indazol-3-yl)-*N*-(prop-2-yn-1-yl)benzamide (**IB-1-24**; **2**)

**IB-1-23** (31 mg) was dissolved in 2 mL of methanol and 2 mL of HCl (6 M aq.). The reaction was stirred at room temperature under Ar atm. The reaction was complete after 2 h. The reaction mixture was extracted with ammonia solution (to increase the pH to ∼8–9). Then, it was extracted three times with DCM, dried with brine and sodium sulfate, and evaporated. The compound was purified by flash chromatography with the following eluant system: A: DCM (1% MeOH), B: ethyl acetate (1% MeOH). Fractions with pure product were combined, evaporated, and dried in high vacuum. The product (20.1 mg) was obtained (81% yield).

^1^H NMR (400 MHz, DMSO-*d*_6_) δ ppm 13.36 (s, 1H), 10.45 (s, 1H), 9.11 (t, *J* = 5.48 Hz, 1H), 8.60 (s, 1H), 8.08–8.19 (m, 3H), 7.61 (d, *J* = 8.44 Hz, 1H), 7.42 (dd, *J* = 7.24 Hz, 1H), 7.26 (dd, *J* = 7.6 Hz, 1H), 6.40–6.55 (m, 1H), 6.32 (dd, *J* = 17.36, 1.88 Hz, 1H), 5.81 (dd, *J* = 9.96, 1.88 Hz, 1H), 4.04–4.10 (m, 2H), 3.12 (t, *J* = 2.44 Hz, 1H). ^13^C NMR (100 MHz, DMSO-*d*_6_) δ ppm 165.90, 163.48, 142.35, 141.64, 139.64, 135.38, 134.36, 131.74, 127.40, 126.27, 121.25, 120.54, 120.15, 119.98, 117.779, 110.77, 81.35, 72.82, 28.65.

##### m

LC-MS (ESI-MS): (/*z*) calculated for C_20_H_17_N_4_O_2_ ([M + H]^+^) 345.1, found 345.3

##### *rac*-3-Acrylamido-5-(1*H*-indazol-3-yl)-*N*-((1-((*trans*-)-2-methoxycyclohexyl)-1*H*-1,2,3-triazol-4-yl)methyl)benzamide (**2a**)

Following general procedure B using *trans*-1-azido-2-methoxycyclohexane, compound **2a** was isolated as a white solid (30% yield). ^1^H NMR (400 MHz, DMSO-*d*_6_) δ 10.45 (s, 1H), 9.23 (t, *J* = 5.7 Hz, 1H), 8.61 (t, *J* = 1.7 Hz, 1H), 8.22–8.11 (m, 3H), 8.01 (s, 1H), 7.63 (d, *J* = 8.4 Hz, 1H), 7.44 (dd, *J* = 8.2, 6.7 Hz, 1H), 7.27 (dd, *J* = 8.2, 6.9 Hz, 1H), 6.49 (dd, *J* = 17.0, 10.0 Hz, 1H), 6.33 (dd, *J* = 17.0, 2.1 Hz, 1H), 5.82 (dd, *J* = 10.1, 2.1 Hz, 1H), 4.56 (d, *J* = 5.7 Hz, 2H), 4.40–4.28 (m, 1H), 4.06–4.02 (m, 1H), 3.93–3.87 (m, 1H), 3.67–3.54 (m, 2H), 3.01 (s, 3H), 2.25–2.16 (m, 1H), 2.02–1.83 (m, 1H), 1.79–1.70 (m, 2H), 1.57–1.46 (m, 2H).

HR-MS (ESI-MS): (*m*/*z*) calculated for C_27_H_30_N_7_O_3_ ([M + H]^+^) 500.2405, found 500.2410, 0.5 ppm deviation. 92% purity estimate based on area under the curve of an LR-UPLC-MS chromatogram measured at 200–498 nm (averaged).

##### 3-Acrylamido-5-(1*H*-indazol-3-yl)-*N*-((1-(*o*-tolyl)-1*H*-1,2,3-triazol-4-yl)methyl)benzamide (**2b**)

Following general procedure B using 2-azidotoluene, compound **2b** was isolated as a white solid (45% yield). ^1^H NMR (400 MHz, DMSO-*d*_6_) δ 13.37 (s, 1H), 10.46 (s, 1H), 9.27 (t, *J* = 5.6 Hz, 1H), 8.62 (t, *J* = 1.8 Hz, 1H), 8.36 (s, 1H), 8.24–8.12 (m, 3H), 7.63 (d, *J* = 8.4 Hz, 1H), 7.52–7.37 (m, 5H), 7.27 (t, *J* = 7.5 Hz, 1H), 6.49 (dd, *J* = 17.0, 10.1 Hz, 1H), 6.33 (dd, *J* = 17.0, 2.1 Hz, 1H), 5.82 (dd, *J* = 10.0, 2.1 Hz, 1H), 4.67 (d, *J* = 5.5 Hz, 2H), 2.18 (s, 3H).

HR-MS (ESI-MS): (*m*/*z*) calculated for C_27_H_23_N_7_O_2_Na ([M + Na]^+^) 500.1811, found 500.1808, 0.6 ppm deviation; >95% purity was estimated based on area under the curve of an LR-UPLC-MS chromatogram measured at 200–498 nm (averaged).

##### 3-Acrylamido-*N*-((1-cyclobutyl-1*H*-1,2,3-triazol-4-yl)methyl)-5-(1*H*-indazol-3-yl)benzamide (**2c**)

Following general procedure B using azidocyclobutane, compound **2c** was isolated as a white solid (50% yield). ^1^H NMR (400 MHz, DMSO-*d*_6_) δ 13.37 (s, 1H), 10.45 (s, 1H), 9.20 (t, *J* = 5.7 Hz, 1H), 8.61 (t, *J* = 1.8 Hz, 1H), 8.17 (dd, *J* = 8.0, 4.3 Hz, 3H), 8.10 (s, 1H), 7.63 (d, *J* = 8.4 Hz, 1H), 7.44 (dd, *J* = 8.3, 6.8 Hz, 1H), 7.27 (t, *J* = 7.5 Hz, 1H), 6.49 (dd, *J* = 16.9, 10.1 Hz, 1H), 6.33 (dd, *J* = 17.0, 2.0 Hz, 1H), 5.82 (dd, *J* = 9.9, 2.1 Hz, 1H), 5.10 (p, *J* = 8.4 Hz, 1H), 4.55 (d, *J* = 5.6 Hz, 2H), 2.59–2.36 (obscured by residual solvent signal, m, 4H), 1.92–1.73 (m, 2H).

HR-MS (ESI-MS): (*m*/*z*) calculated for C_24_H_24_N_7_O_2_ ([M + H]^+^) 442.1991, found 442.1985, 1.6 ppm deviation; >95% purity was estimated based on area under the curve of an LR-UPLC-MS chromatogram measured at 200–498 nm (averaged).

##### 3-Acrylamido-5-(1*H*-indazol-3-yl)-*N*-((1-(2-methoxyethyl)-1*H*-1,2,3-triazol-4-yl)methyl)benzamide (**2d**)

Following general procedure B using 3-azido-1-methoxyethane, compound **2d** was isolated as an off-white solid (60%). ^1^H NMR (400 MHz, DMSO-*d*_6_) δ 13.37 (s, 1H), 10.46 (s, 1H), 9.23 (t, *J* = 5.8 Hz, 1H), 8.61 (t, *J* = 1.8 Hz, 1H), 8.25–8.10 (m, 3H), 7.95 (s, 1H), 7.63 (d, *J* = 8.4 Hz, 1H), 7.44 (dd, *J* = 8.4, 6.7 Hz, 1H), 7.27 (dd, *J* = 8.1, 6.9 Hz, 1H), 6.49 (dd, *J* = 17.0, 10.1 Hz, 1H), 6.33 (dd, *J* = 16.9, 2.1 Hz, 1H), 5.82 (dd, *J* = 10.0, 2.1 Hz, 1H), 4.66–4.35 (m, 4H), 3.72 (t, *J* = 5.2 Hz, 2H), 3.24 (s, 3H).

HR-MS (ESI-MS): (*m*/*z*) calculated for C_23_H_23_N_7_O_3_Na ([M + Na]^+^) 468.1760, found 468.1755, 1.5 ppm deviation; >95% purity was estimated based on area under the curve of an LR-UPLC-MS chromatogram measured at 200–498 nm (averaged).

##### 3-Acrylamido-*N*-((1-(3-chloro-4-fluorophenyl)-1*H*-1,2,3-triazol-4-yl)methyl)-5-(1*H*-indazol-3-yl)benzamide (**2e**)

Following general procedure B using 4-azido-2-chloro-1-fluorobenzene, compound **2e** was isolated as a white solid (41% yield). ^1^H NMR (400 MHz, DMSO-*d*_6_) δ 13.37 (s, 1H), 10.46 (s, 1H), 9.31 (t, *J* = 5.7 Hz, 1H), 8.78 (s, 1H), 8.66–8.58 (m, 1H), 8.34–8.13 (m, 4H), 8.00 (ddd, *J* = 9.0, 4.1, 2.6 Hz, 1H), 7.74–7.59 (m, 2H), 7.52–7.37 (m, 1H), 7.28 (t, *J* = 7.5 Hz, 1H), 6.49 (dd, *J* = 16.9, 10.0 Hz, 1H), 6.34 (dd, *J* = 16.8, 2.0 Hz, 1H), 5.83 (dd, *J* = 10.0, 2.1 Hz, 1H), 4.65 (d, *J* = 5.5 Hz, 2H).

HR-MS (ESI-MS): (*m*/*z*) calculated for C_26_H_19_N_7_O_2_FNaCl ([M + Na]^+^) 538.1170, found 538.1166, 0.7 ppm deviation. 70% purity estimate based on area under the curve of an LR-UPLC-MS chromatogram measured at 200–498 nm (averaged).

#### Tissue Culture

U2OS (ATCC HTB-96) cells were maintained in high-glucose DMEM, supplemented with 10% fetal bovine serum, 1% glutamine 200 mM, 1% sodium pyruvate 100 mM, and 1% penicillin–streptomycin solution (all reagents from Biological Industries, Beit Haemek, Israel), incubated at 37 °C with 5% CO_2_.

#### Procedure for In-Cell Western (ICW) Detection of p-JNK

384-Well clear-bottom black flat plates (Corning) were coated with fibronectin (Sigma) diluted in PBS to a concentration of 5 mg/mL for 45 min, aspired, and 4 × 10^5^ U2OS cells/mL were plated using Multidrop Combi reagent dispenser (Thermo Fisher Scientific), 50 μL per well, and left in an incubator for 24 h. Compound, reagents, and antibody dispensing as well as washes were performed using CyBio liquid handler (Analytik Jena). To create compound plates, 1.5 μL of 3.33 mM compounds were taken from plates 1 and 2 and diluted to 83.33 μM by completing to 60 μL volume (PBS) in 384-well plates. The cells were preincubated with 10 μL from the compound plate on cells (total of 60 μL, series **1**: 13.89 μM, series **2**: 10 μM) for 2 h before fixation. U2OS cells were treated with 0.2 M d-Sorbitol/PBS (Sigma) for 40 min before fixation, except for control wells that were added with PBS. Media was removed, and wells were immediately fixed with 150 μL per well of 3.7% formaldehyde/PBS fixing solution for 20 min. The fixing solution was then removed, and the cells were permeabilized with 150 μL of 0.5% Triton X-100/PBS solution per well for 10 min. After aspiration, the cells were washed with 50 μL per well of ice-cold methanol and stored at −20 °C, for 10 min. The methanol was aspirated, and the cells were blocked with 150 μL of blocking buffer (LICOR) for 90 min. Primary antibody Phospho-SAPK/JNK T183/Y185 (CST; 9251S) was diluted 1:1000 in blocking buffer and was applied 50 μL per well, except control wells to which only blocking buffer was applied, O.N. (16 h) at 4 °C with mild shaking. Primary antibody was aspired, and the wells were washed with 150 μL of 0.1% Tween-20/PBS (Sigma) washing solution three times for 7 min. IRDye 800CW Goat anti-Rabbit IgG Secondary antibody (LICOR) was then diluted 1:1200 in blocking buffer with 50 μL applied per well on control wells only (no CellTag control), then CellTag700 (1:2000) was added to the solution, and 50 μL was added to all experiment wells for 1 h at room temperature. The wells were aspirated and washed again with 150 μL of 0.1% Tween-20/PBS (Sigma) washing solution three times for 7 min. The plates were dried and scanned with Odyssey CLx imaging system at 700 nm and 800 nm (LICOR). Well signals were analyzed with Odyssey software. The secondary antibody signal was normalized by the CellTag700 signal. Curve fitting was performed using Prism software (GraphPad), using the log(inhibitor) *vs* response-variable slope (four parameters).

#### Western Blotting

U2OS cells were incubated a day before the experiment in a six-well plate. On the day of the experiment, the cells underwent media change and were incubated with the compound containing 0.1% DMSO, or DMSO only, for 80 min. Sorbitol was then added to the concentration of 0.2 M for 40 min, except for an “untreated” well. After 40 min, the cells were harvested using Trypsin B and were washed with ice-cold PBS. The cells were lysed using a mixture of 50 μL of RIPA buffer (Sigma; R0278) and a protease inhibitor (Sigma; P8340) for 20 min, in which every 5 min, the cells undergo a 5 s vortex. After 20 min, cell lysates underwent a 20 min centrifuge at 4 °C at 21,000 rpm. The lysates were then separated from the cell membrane remains. Protein concentration was determined by the BCA analysis, and 35 μg of protein was taken from each sample, supplemented with 4× loading buffer that has 0.02 mM DTT, and heated for 10 min at 70 °C. Samples were loaded into a 4–20% SurePAGE bis-tris gel (GenScript; M00655/M00656/MO00657) at 140 V for 70 min and then transferred into nitrocellulose membrane (Biorad) using Trans-Blot Turbo transfer system (Biorad; 1704158). The membrane was stained with Ponceau (Sigma; 6226-79-5) to assure the quality of the transfer. The membrane was de-stained with ddH_2_O and then blocked for 60 min with a blocking buffer that is composed of 5% BSA in TBS-T. Next, the membrane was incubated overnight at 4 °C with anti-rabbit p-c-Jun (Ser63) II antibody (CST; 9261S). The next day, the membrane was washed three times for 10 min with TBS-T. The membrane was incubated with anti-rabbit IgG, HRP-linked secondary antibody (CST; 7074S) for 1 h at room temperature, and washed four times for 7 min each with TBS-T. The antibody was then mixed for 90 s with its substrate using an EZ-ECL Kit (Biological Industries; 20-500-1000) and imaged using ChemiDoc XRS+ (BioRad). Following the imaging, the membrane was washed three times for 10 min with TBS-T and then stripped of the antibodies using Restore Western Blot Stripping Buffer (Thermo Fisher; 21059) for 15 min. The membrane was washed again with TBS-T three times for 10 min and reincubated with the blocking buffer for 30 min. After that, the membrane was incubated with the housekeeping primary antibody GAPDH (CST; 5174) at room temperature for an hour. The protocol repeats itself, using an anti-mouse HRP secondary antibody instead.

Quantification was done using ImageJ software. The EC_50_ values were determined using PRISM, applying a sigmoidal dose–response equation against the logarithmic compound concentration model and constraining the top to 1 and the bottom to 0.

#### Protein Expression, Purification, and Crystallization

The used construct of MKK7 contained amino acids 117–423 with a noncleavable N-terminal His_6_-tag. The protein was expressed in BL21 DE3 *Escherichia coli* at 18 °C for 20 h, and the cells were lysed using French press. After centrifugation, the supernatant was loaded on a Ni-affinity chromatography (Qiagen Ni-NTA Superflow 5 mL), washed with buffer A (50 mM Tris, 500 mM NaCl, 25 mM imidazole, 5% glycerol, pH 8), and eluted with a gradient of buffer B (50 mM Tris, 500 mM NaCl, 500 mM imidazole, 5% glycerol, pH 8). The protein was concentrated and applied to a gel filtration chromatograph (GE HiLoad 16/600 75pg) using buffer C (25 mM Tris, 100 mM NaCl, 10% glycerol, pH 7.4). The eluted protein fractions were combined and concentrated to 23 mg/mL and directly used for crystallization.

Crystallization of MKK7 was performed either by co-crystallization or soaking protocols. In the case of co-crystallization, 10 mg/mL MKK7 was incubated with a 3-fold molar excess of inhibitor (10 mM DMSO stock) for 1 h at 4 °C. Crystals were grown using the hanging drop method at 4 °C after mixing 1 μL of protein–inhibitor solution with 1 μL of reservoir solution (180–220 mM sodium citrate, 15–25% PEG3350). After 3 days, needle-shaped crystals of sufficient size for diffraction analysis were obtained. Following soaking procedures, apo-protein was crystallized analogous to described above, but in the absence of compound. The corresponding apo-crystals were incubated in a separate reservoir containing a 0.5 μL drop, 30% (v/v) glycerol, and 1 mM inhibitor (10% final DMSO content) for 24 h.

When necessary, the crystals were cryo-protected with 20–25% (v/v) glycerol and all crystals were subsequently cooled in liquid N_2_. The datasets were collected at the PXII X10SA beamline of the Swiss Light Source (PSII, Villigen, Switzerland). All datasets were processed with XDS and scaled using XSCALE.^45^ Structure determination and refinement of the complex crystal structures were solved by molecular replacement with phaser^46^ using PDB entry 6QFL as template. The MKK7 molecule in the asymmetric unit was manually adjusted using the program COOT.^47^ The refinement was performed with Phenix.refine.^48^ Inhibitor topology files were generated using the Dundee PRODRG2 server^49^ and Phenix.elbow,^48^ respectively. Refined structures were validated with the PDB validation server. Data collection, structure refinement statistics, PDB codes, and further details for data collection are provided in Table 1. PyMOL was used for generating Figures 4 and S9.^50^

#### General Procedure for LC-MS Labeling

LC/MS runs were performed on a Waters ACUITY UPLC class H instrument, in positive-ion mode using electrospray ionization. UPLC separation for small molecules used a C18 column of 1.7 μm, 2.1 mm × 50 mm, for all of the LC-MS-based assays.

The purified compounds (2 μM) with the addition of 5 μM ATP and 2 μM MKK7 protein (see the protein expression above) were incubated for 10 min at 4 °C. After 10 min, 5 μL of 4.4% formic acid was added to the final concentration of 0.4% in the well. Next, they were run in the LC/MS at 10 °C. *n* = 2, except for compounds that showed high variability, and therefore were repeated (*n* = 3 for **1i**, *n* = 4 for **1c**, **1d**, **1h**, **3**), while having compound **4** for reference, to make sure that the LC/MS labeling conditions were kept the same. The buffer was 25 mM Tris, 100 mM NaCl, and 5 mM MgCl_2_ at pH 7.4 at 4 °C.

#### *In Vitro* Kinase Activity Assays (Carried Out by Nanosyn, Santa Clara, CA)

Test compounds were diluted in DMSO to a final concentration that ranged from 10 μM to 0.0565 nM, while the final concentration of DMSO in all assays was kept at 1%. The reference compound, Staurosporine, was tested in a similar manner as a positive control. MKK7 (1 nM) was preincubated with inactive JNK1 in a buffer comprising 100 mM HEPES, 5 mM MgCl_2_, 1 mM DTT, 0.1% BSA, 0.01% Triton X-100, and 2 μM ATP for 2 h. After incubation (17 h), the activity of JNK1 activated by MKK7 was tested in the presence of 30 μM ATP.

#### Pull-Down Sample Preparation

U2OS cells (10–15 million) were grown per sample and incubated as follows: DMSO-treated samples: 0.1% DMSO for 2 h, followed by 0.1% DMSO for 2 h; **1-**treated samples: 0.1% DMSO for 2 h, followed by 5 μM **1** in 0.1% DMSO for 2 h; **2**-treated samples: 5 μM **2** in 0.1% DMSO for 2 h. The samples were prepared in quadruplicates, except for the DMSO-treated samples that were prepared in triplicate.

The cells were washed with cold PBS, scraped from the plates, and frozen. Then, each sample was lysed in 200 μL of RIPA for 15 min on ice and centrifuged at 20,000*g* at 4 °C. The protein in the supernatant was quantified using BCA. For each sample, 250 μL of 1.7 mg/mL was prepared. At this point, 5 μL of 5 mM biotin azide and 9 μL of 100 mM CuSO_4_/THPTA complex were added. The click reaction was initiated by the addition of 7.5 μL of 150 mM sodium ascorbate, and the samples are incubated at room temperature for 1 h. The samples were then precipitated with methanol/chloroform (1 mL methanol, 250 μL chloroform, 750 μL water), washed with 1 mL of methanol, and air-dried.

The dry pellet was resuspended in 1.2% SDS in Ca/Mg free PBS (250 μL), sonicated (4 × 2 s with 2 s off), and heated to 95 °C for 5 min. The samples were then diluted to 1.5 mL with PBS, and 50 μL of streptavidin agarose beads, prewashed with 0.2% SDS in PBS, was added, followed by 3 h incubation at room temperature. Following the incubation, the beads were centrifuged at 2000*g* for 2 min and washed four times with the following buffers (4 mL in each wash): 2% SDS; 0.1% sodium deoxycholate, 1% Triton X-100, 0.5 M NaCl, 1 mM EDTA, 50 mM HEPES pH = 7.5; 0.25 M NaCl, 0.5% IGEPAL, 0.5% sodium deoxycholate, 1 mM EDTA, 10 mM Tris pH = 8.1; 50 mM Tris pH = 7.4, 50 mM NaCl.

Following the last wash, the buffer was removed and 100 μL of 7.5% SDS in Tris 50 mM pH = 8 was added, with heating to 95 °C for 6 min and occasional vortexing. Finally, the beads were spun down (2 min 2000*g*) and 80 μL of supernatant was removed to separate tubes.

Then, 4 μL of 0.1 M DTT was added and samples were incubated at 65 °C for 45 min. After the samples had cooled, 4 μL of iodoacetamide (0.2 M) were added, and the samples were incubated in the dark for 40 min at room temperature. At this point, 1/10 volume of 12% phosphoric acid was added, and the samples were diluted 6-fold with 90% methanol + 50 mM ammonium bicarbonate. The samples were then loaded on s-trap micro columns (Protify), and the columns were washed three times with 150 μL of 90% methanol + 50 mM ammonium bicarbonate. Then, 20 μL of 0.05 μg/μL of trypsin in 50 mM ammonium bicarbonate was added to the columns and the samples were incubated at 47 °C for 90 min. Then, 40 μL of 50 mM ammonium bicarbonate was added, followed by centrifugation and addition of 1 μL of 0.5 μg/μL trypsin to the eluate, which was incubated at 37 °C overnight. The column itself was then eluted using 40 μL of 0.2% formic acid and 40 μL 0.2% formic acid in 50% acetonitrile into a separate tube, which was kept at 4 °C. The two eluates were then combined and evaporated. The samples were further desalted using Oasis desalting columns (Waters) and then evaporated again and dissolved in 30 μL of 3% acetonitrile with 0.1% formic acid.

#### LC/MS/MS Analysis

Samples were analyzed using EASY-nLC 1200 nano-flow UPLC system, using PepMap RSLC C18 column (2 μm particle size, 100 Å pore size, 75 μm diameter × 50 cm length), mounted using an EASY-Spray source onto an Exploris 240 mass spectrometer. uLC/MS-grade solvents were used for all chromatographic steps at 300 nL/min. The mobile phase was: (A) H_2_O + 0.1% formic acid and (B) 80% acetonitrile + 0.1% formic acid. Each sample (2 μL) was injected. Peptides were eluted from the column into the mass spectrometer using the following gradient: 1–40% B in 160 min, 40–100% B in 5 min, maintained at 100% for 20 min, 100 to 1% in 10 min, and finally 1% for 5 min. Ionization was achieved using a 1900 V spray voltage with an ion transfer tube temperature of 275 °C. Data were acquired in data-dependent acquisition (DDA) mode. MS1 resolution was set to 120,000 (at 200 *m*/*z*), a mass range of 375–1650 *m*/*z*, normalized AGC of 300%, and the maximum injection time was set to 20 ms. MS2 resolution was set to 15,000, quadrupole isolation 1.4 *m*/*z*, normalized AGC of 50%, dynamic exclusion of 45 s, and automatic maximum injection time.

#### Proteomics Data Analysis

The data were analyzed using MaxQuant 1.6.0.16. Human Proteome fasta file downloaded on March 2022 was used, and contaminants were included. The digestion enzyme was set to Trypsin/P with a maximum number of missed cleavages of 2. Oxidation of methionine and N terminal acetylation were included as variable modifications. The “Re-quantify” option was enabled. Carbamidomethyl (C_2_H_3_NO) was used as fixed modification on cysteine. Contaminants were included. Peptides were searched with a minimum peptide length of 7 and a maximum peptide mass of 6500 Da. “Second peptides” was enabled, “Dependent peptides” were disabled, and the option “Match between run” was enabled with a Match time window of 0.7 min and an alignment window of 20 min. An FDR of 0.01 was used for Protein FDR, PSM FDR, and XPSM FDR. Proteins were identified and quantified based on the label-free quantification (LFQ)^51^ values reported by MaxQuant. Following MaxQuant analysis, proteins identified only through razor peptides or modified peptides, as well as common contaminants, were removed, and only proteins that gave at least three nonzero LFQ intensity values in at least one of the datasets were retained for analysis. Missing values were then set so Log2(LFQ intensity) was 15.

To identify proteins significantly enriched by molecules, the datasets were analyzed using Student’s *t*-test, and proteins exhibiting a *p*-value lower than 0.01 and more than 4-fold enrichment (log difference larger than 2) were retained. This analysis yielded two proteins enriched by molecule **1** and 720 proteins enriched by molecule **2**.

## Supporting Information Available

Binding modes or parent alkynes; plots of chemical properties of triazole libraries; LC/MS labeling of resynthesized compounds; omit maps of the co-crystalized compounds; raw Western blots; model of 1e binding mode; GSH consumption assay results; pulldown proteomics enrichment results; crystallographic statistics table for new structures; and ^1^H and ^13^C spectra and HPLC traces for synthesized compounds (PDF)
Molecular formula strings (CSV)
Supplementary Datasets including azide library coded and SMILES and detailed proteomics results (XLS)

## Accession Codes

PDB ID Code: Coordinates and structure factors have been deposited at the PDB with PDB codes: 7OVK, 7OVI, 7OVJ, 7OVL, 7OVN, 7OVM. The authors will release the atomic coordinates upon article publication.

## Abbreviations Used

CuAAC: copper(I)-catalyzed alkyne–azide cycloaddition
DELs: DNA-encoded libraries
ICW: In Cell Western
LSF: late-stage functionalization
MAPK: mitogen-activated protein kinase
SAR: structure–activity relationship
TBTA: tris((1-benzyl-4-triazolyl)methyl)amine
