# Supplementary material for: Optimization of Covalent MKK7 Inhibitors via Crude Nanomole-Scale Libraries
Source: J Med Chem. 2022 Jul 30;65(15):10341–56. doi: 10.1021/acs.jmedchem.1c02206 (PMC9376956; doi:10.1021/acs.jmedchem.1c02206)

Supporting Information

Optimization of covalent MKK7 inhibitors via crude nanomole-scale libraries

Paul Gehrtz[1,2,#], Shir Marom[1,#], Mike Bührmann[3,4,#], Julia Hardick[3,4,#], Silke Kleinbölting[3,4], Amit Shraga[1,5], Christian Dubiella[1,6], Ronen Gabizon[1], Jan N. Wiese[3,4], Matthias P. Müller[3,4], Galit Cohen[7], Ilana Babaev[7], Khriesto Shurrush[7], Liat Avram[8], Efrat Resnick[1], Haim Barr[7], Daniel Rauh[3,4,*], Nir London[1,*]

[1] Department of Chemical and Structural Biology, Weizmann Institute of Science, 7610001 Rehovot, Israel

[3] Department of Chemistry and Chemical Biology, TU Dortmund University, Otto-Hahn-Strasse 4a, 44227, Dortmund, Germany.

[4] Drug Discovery Hub Dortmund (DDHD) am Zentrum für integrierte Wirkstoffforschung (ZIW), 44227 Dortmund, Germany

[7] The Nancy and Stephen Grand Israel National Center for Personalized Medicine, Weizmann Institute of Science, 7610001 Rehovot, Israel.

[8] Department of Chemical Research Support, Weizmann Institute of Science, 7610001, Rehovot, Israel

[#] These authors contributed equally.

[*] Corresponding:

N.L.: nir.london@weizmann.ac.il

D.R.: daniel.rauh@tu-dortmund.de

Keywords: CuAAC, Click-Chemistry, Covalent Inhibitors, MKK7, High-Throughput Chemistry

Contents

Figure S1. The binding modes of alkynes **1** and **2** in the ATP binding site of MKK7 S2
Figure S2. Plot of cLogS of series **1** members and their associated p-JNK levels S3
Figure S3. Plot of cLogP of series **1** members and their associated p-JNK levels S3
Figure S4. Plot of tPSA of series **1** members and their associated p-JNK levels S4
Figure S5. Plot of cLogS of series **2** members and their associated p-JNK levels S4
Figure S6. Plot of cLogP of series **2** members and their associated p-JNK levels S5
Figure S7. Plot of tPSA of series **2** members and their associated p-JNK levels S5
Figure S8. Intact protein LC/MS labelling experiment of re-synthesized compounds S6
Figure S9. Omit maps of the co-crystallized MKK7 inhibitors S6
Figure S10. Dose response western blots with p-c-Jun S7-S8
Figure S11. A model of compound **1e** based on the structure of MKK7 in complex with **1k** S9
Figure S12. Biphasic in vitro kinase inhibition behavior by alkyne **2.** S9
Figure S13. GSH consumption assay of selected compounds **1**, **1b**, **1k**, **2**, and **2b**. S10
Figure S14. Pull down proteomics analysis of alkynes **1** and **2.** S11
Figure S15. Chemical structure of compound **4**. S11
Table S1. Crystallographic statistics of MKK7 in complex with triazolyl inhibitor derivatives. S12
HPLC and NMR spectra of reported compounds S13-S52

Supplementary Figures

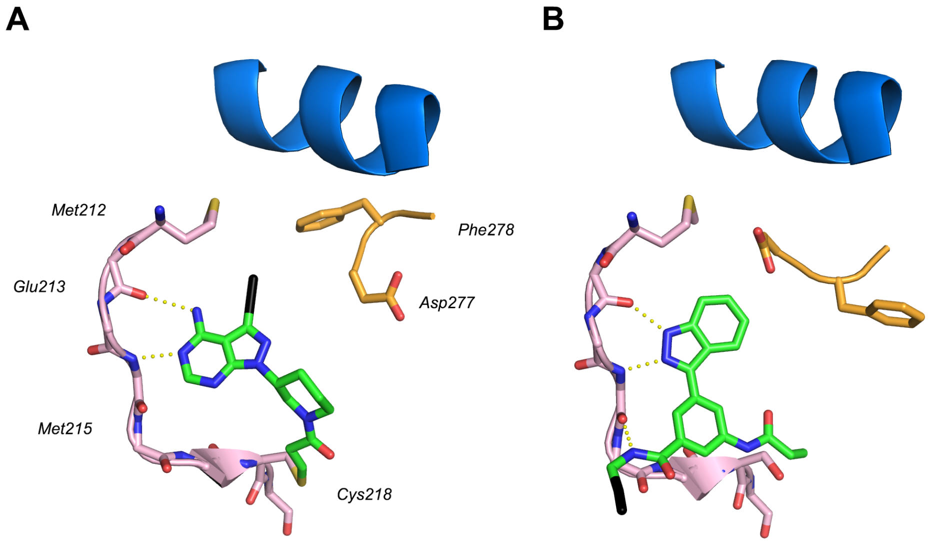

Figure S1. The binding modes of alkynes **1** and **2** in the ATP binding site of MKK7. (A) **1** co-crystalized in complex with MKK7 (PDB: 6IB0), where the alkyne (black) is protruding into the back pocket. (B) **2** co-crystalized in complex with MKK7 (PDB: 7CBX), where the alkyne (black) is pointing outside the binding pocket towards the solvent-exposed region.

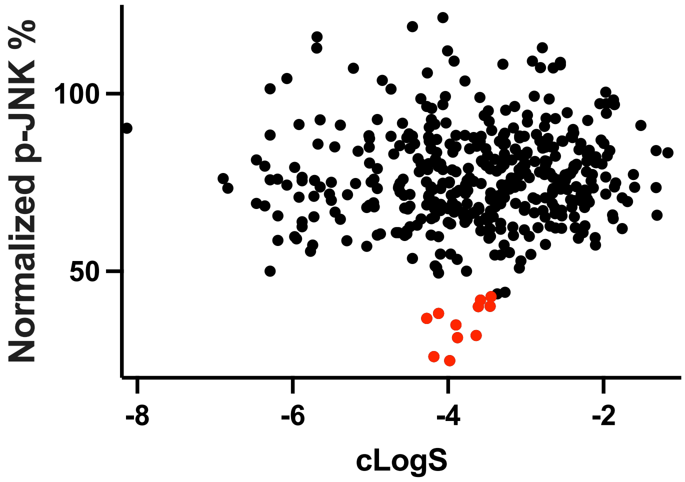

Figure S2. Plot of cLogS of series **1** members and their associated p-JNK levels determined via an ICW assay (13.8 µM, 2 h, U2OS cells). Top ten compounds from the series are highlighted in red.

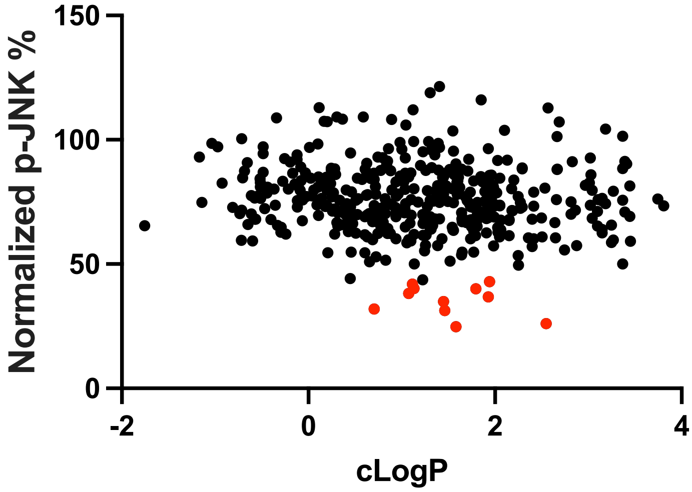

Figure S3. Plot of cLogP of series **1** members and their associated p-JNK levels determined via an ICW assay (13.8 µM, 2 h, U2OS cells). Top ten compounds from the series are highlighted in red.

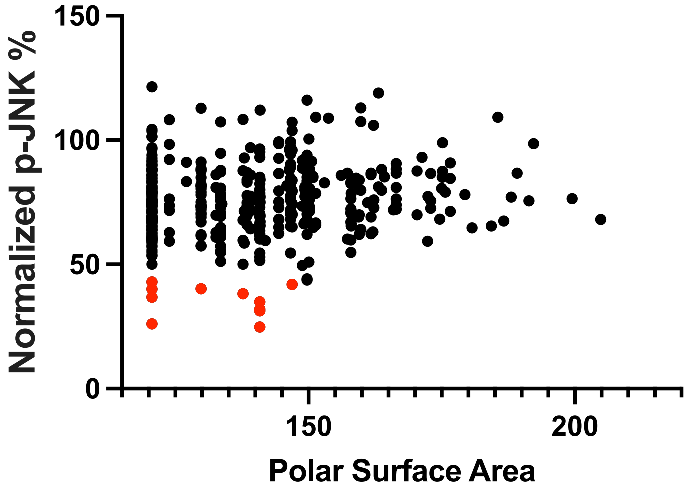

Figure S4. Plot of tPSA of series **1** members and their associated p-JNK levels determined via an ICW assay (13.8 µM, 2 h, U2OS cells). Top ten compounds from the series are highlighted in red.

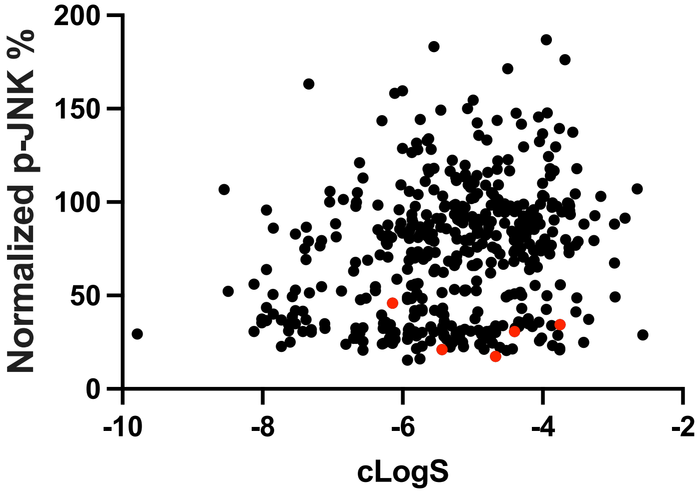

Figure S5. Plot of cLogS of series **2** members and their associated p-JNK levels determined via an ICW assay (10 µM, 2 h, U2OS cells). Selected five compounds from the series are highlighted in red.

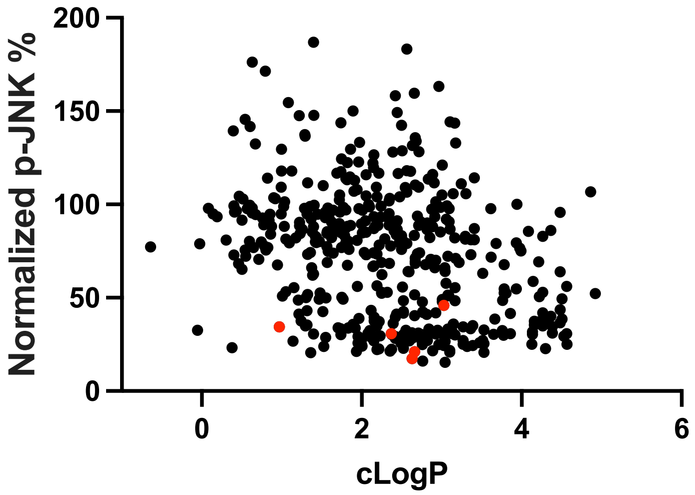

Figure S6. Plot of cLogP of series **2** members and their associated p-JNK levels determined via an ICW assay (10 µM, 2 h, U2OS cells). Selected five compounds from the series are highlighted in red.

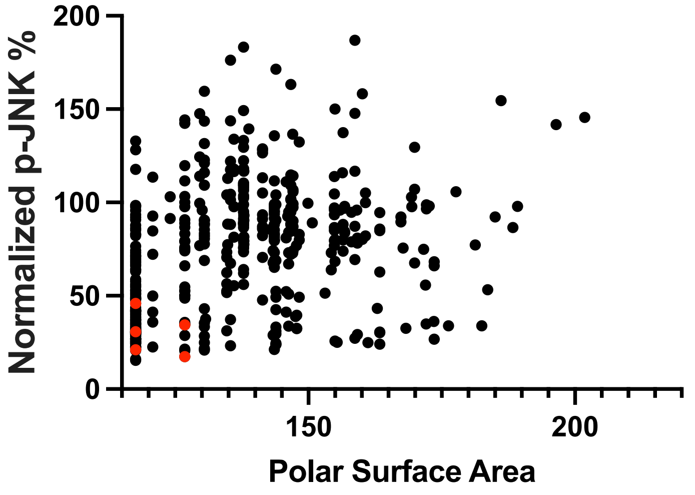

Figure S7. Plot of tPSA of series **2** members and their associated p-JNK levels determined via an ICW assay (13.8 µM, 2 h, U2OS cells). Selected five compounds from the series are highlighted in red.

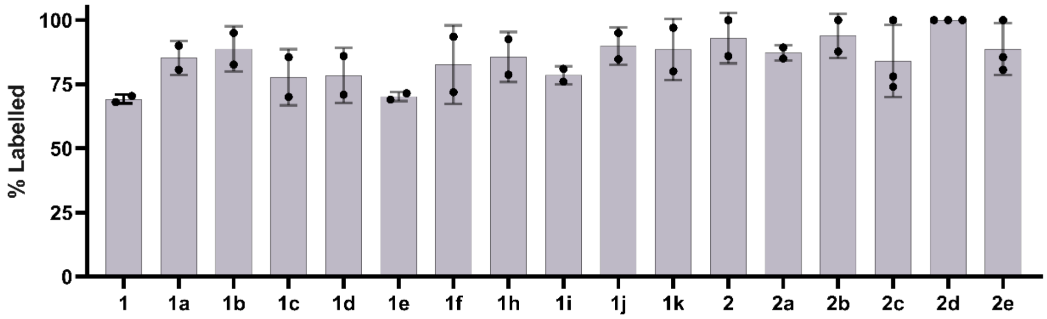

Figure S8. Intact protein LC/MS labelling experiment of re-synthesized compounds derived from top crude screening hits. Reaction conditions: 2 µM compound, 2 µM protein, 10 min, 4 °C, quenched with formic acid to a final concentration of 0.4% (v/v). In series **1**, all except compounds **1e**, showed improved binding abilities compared to precursor **1** with over 75% labelling. In series **2**, all compounds showed high binding abilities of over 75%.

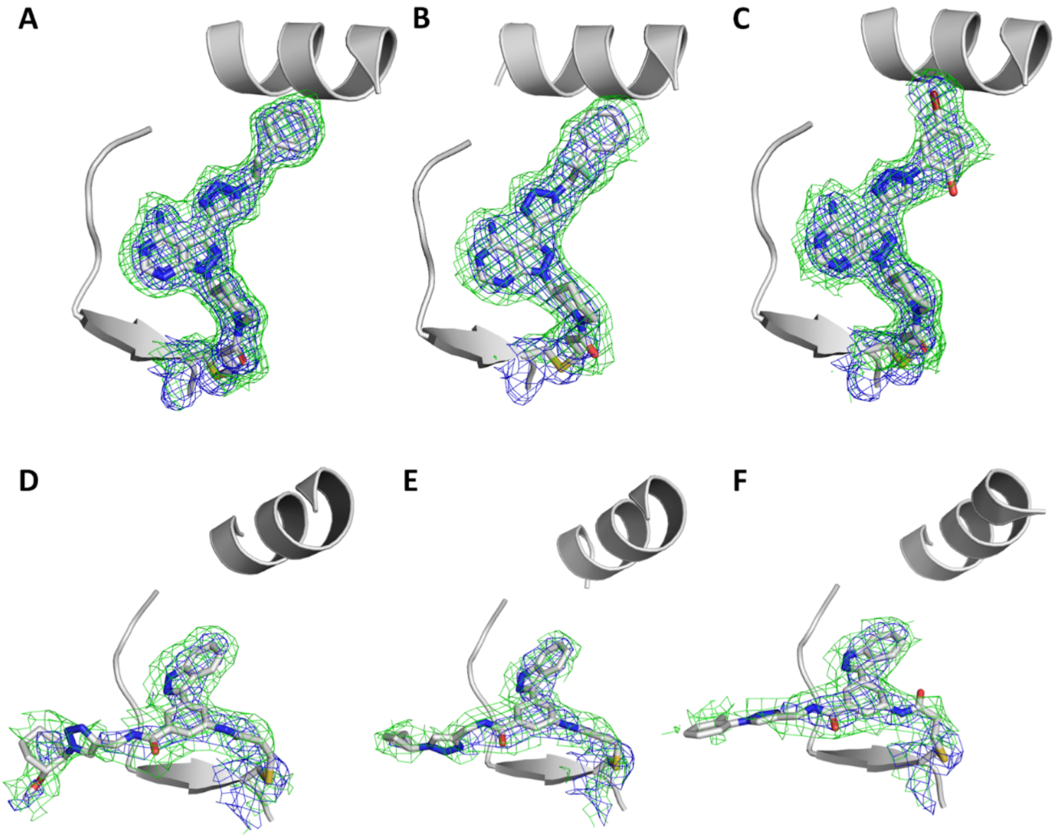

Figure S9. Omit maps of the co-crystallized MKK7 inhibitors. Performing a simulated annealing refinement, mFo-DFc omit maps (green, contoured at 2.5σ) were calculated for (A) **1h** (B) **1k**, (C) **1a**, (D) **2b**, (E) **2d**, and (F) **2c** as well as for the targeted Cys218. 2Fo-Fc maps for the ligands and Cys218 were contoured at 1.0σ (blue). Maps indicate partial occupancy for the compounds of series **2** in the area of the solvent-exposed triazolyl moiety.

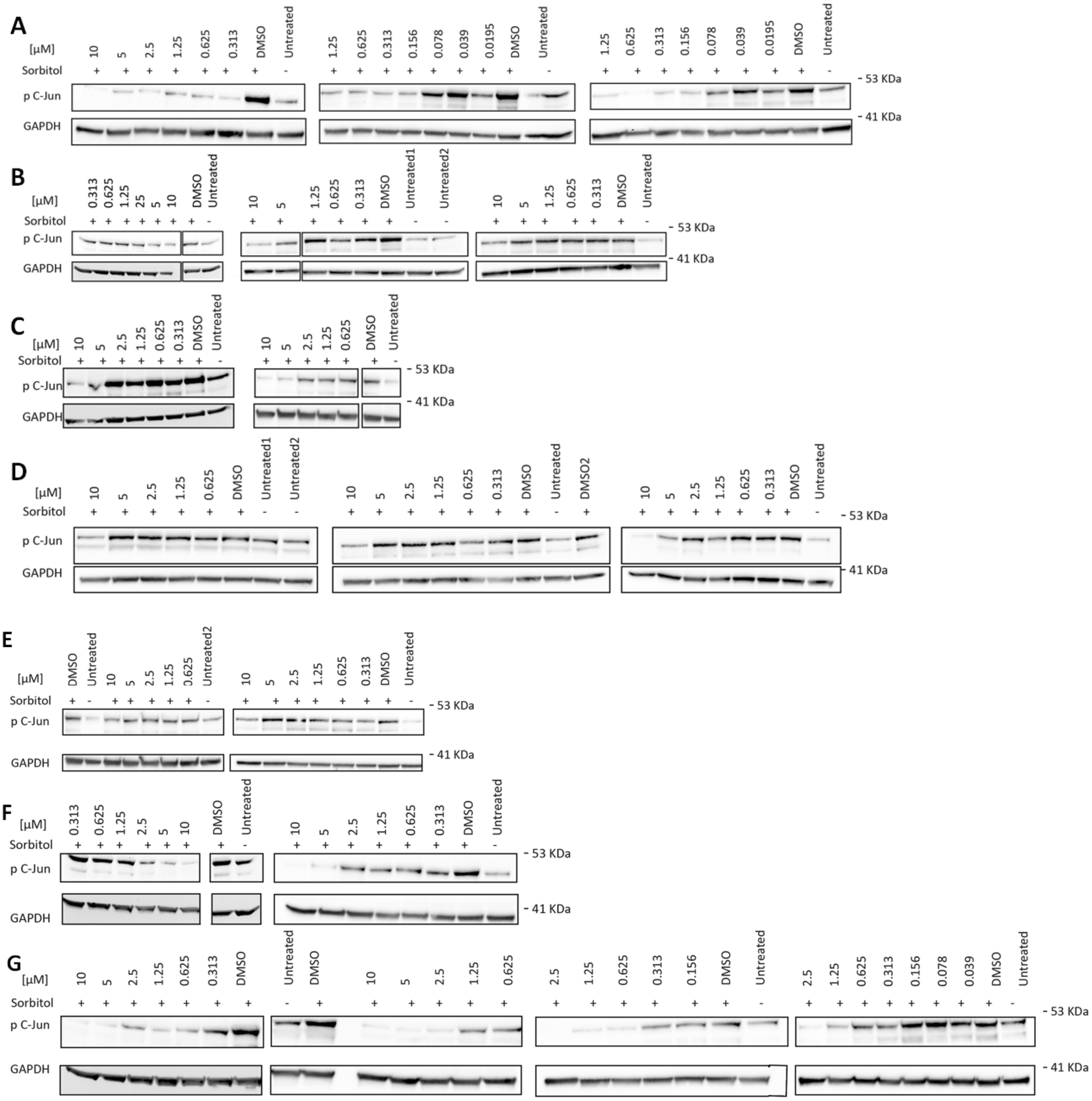
A
B
C
D
E
F
G
[µM]
Sorbitol
p C-Jun
GAPDH
10
5
2.5
1.25
0.625
0.313
0.156
0.078
0.039
0.0195
DMSO
DMSO2
Untreated
Untreated1
Untreated2
25
- 53 KDa
- 41 KDa

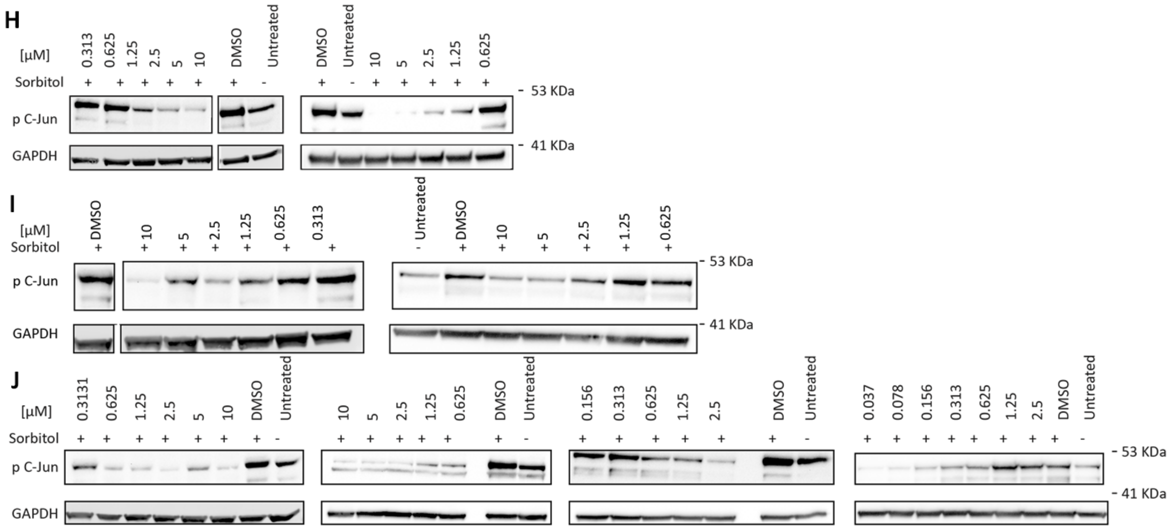

Figure S10. Dose response western blot with p-c-Jun as the readout and GAPDH as the housekeeping gene for normalization. (A) **2**, (B) **2a**, (C) **2b**, (D) **2c**, (E) **1**, (F) **1a**, (G) **1b**, (H) **1d**, (I) **1e**, (J) **1k**.

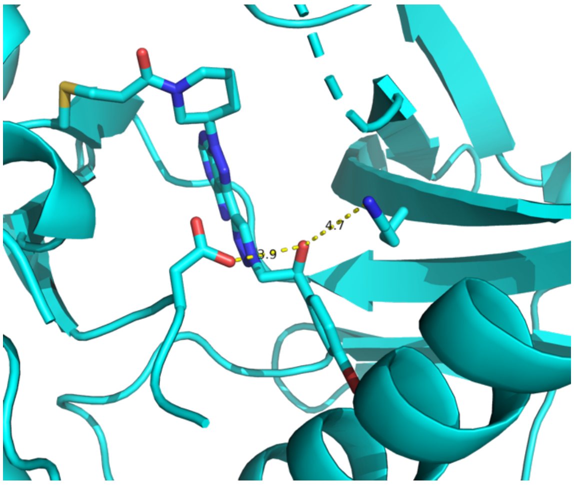

Figure S11. A model of compound **1e** based on the co-crystal structure of MKK7 in complex with **1k** shows the hydroxy side-chain may form a hydrogen bond with either Asp277 (similar to **1a**; see Fig. 4), or with Lys155. Note that the side-chains were not remodeled and may in fact adopt a rotamer that places closer to the hydroxy moiety.

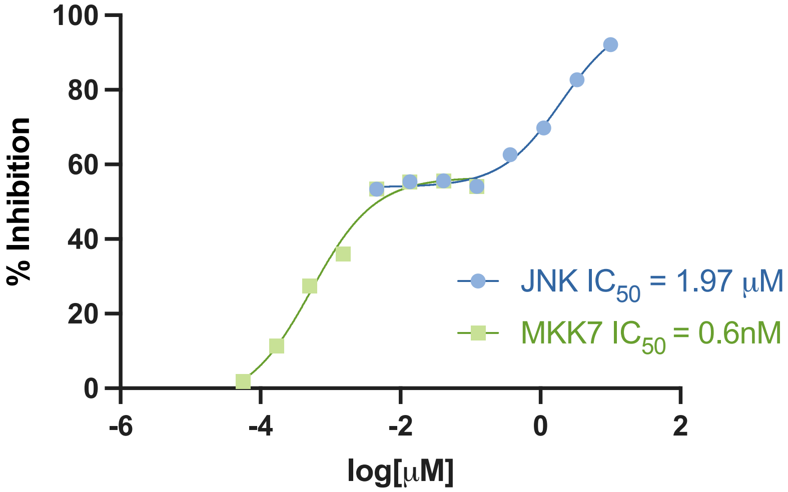

Figure S12. Biphasic in vitro kinase inhibition behavior by alkyne **2.** In a coupled JNK/ MKK7 in vitro kinase activity assay, alkyne **2** shows a biphasic inhibition curve. Each sigmoidal curve can be fitted separately with very potent inhibition of MKK7 at lower concentrations (IC_{50} = 0.6nM; R^2=0.986) and likely weak inhibition of JNK at higher concentrations (IC_{50}=1.975 μM; R^2=0.966).

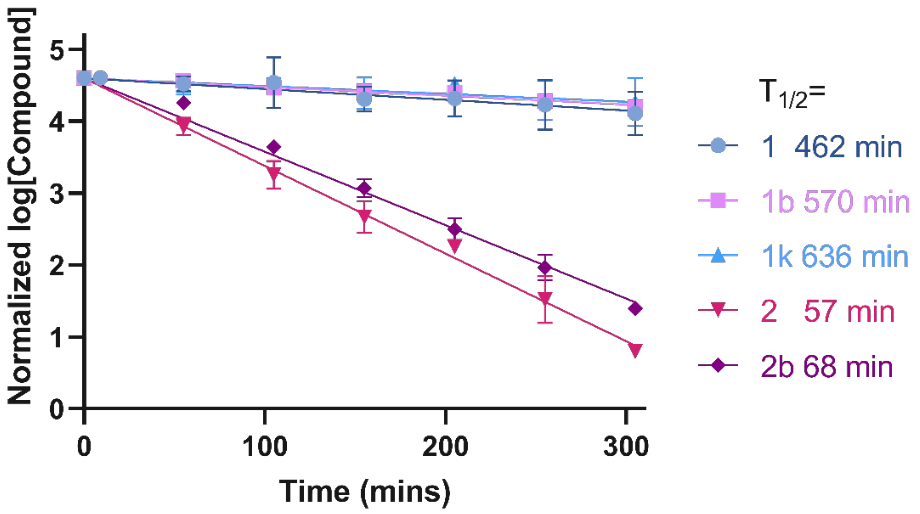

Figure S13. GSH consumption assay of selected compounds **1**, **1b**, **1k**, **2**, and **2b**. The assay was done at 37 °C using 100 µM reference compound 4-nitrobenzonitrile, 100 µM of tested compounds and 5 mM GSH. The buffer was 90% PBS and 10% DMF. Every 50 min, the vial was taken from the incubator, shaken well, and then 50 µL was taken and analyzed in the LC/MS. The series **1** compounds were significantly more stable and less reactive than **2** and **2b**. **2b** appeared slightly less reactive than the scaffold **2**.

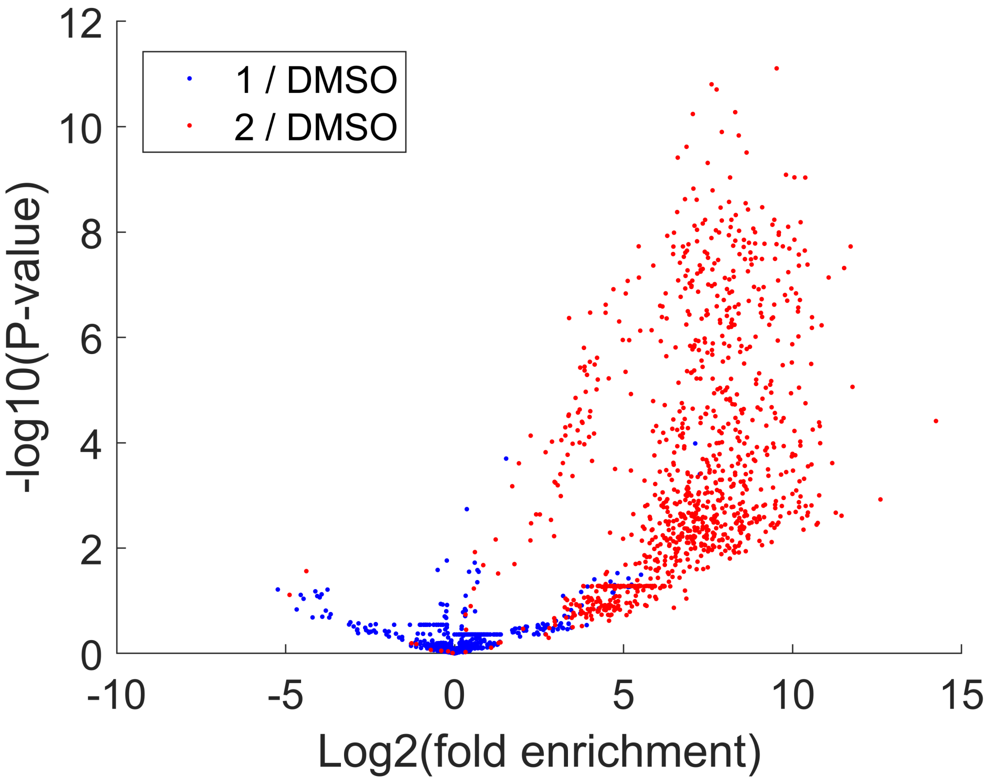

Figure S14. Pull down proteomics analysis of alkynes **1** and **2.** Volcano plot for proteins identified in pull-down proteomics experiments using molecules **1** and **2**, the x-axis shows Log2 fold enrichment of proteins detected in samples treated with either alkyne compared to DMSO. The y-axis shows the significance of the difference.

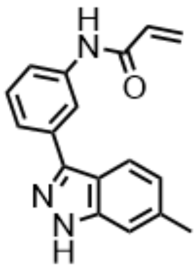

Figure S15. Chemical structure of compound **4**.

Supplementary Tables

	1a (PDB: 7OVK)	1h (PDB: 7OVI)	1k (PDB: 7OVJ)	2a (PDB: 7OVL)	2b (PDB: 7OVN)	2c (PDB: 7OVM)
**Data collection**						
**Space group**	P 2_1 2_1 2_1	P 2_1 2_1 2_1	P 2_1 2_1 2_1	P 2_1 2_1 2_1	P 2_1 2_1 2_1	P 2_1 2_1 2_1
**Cell dimensions**						
a, b, c (Å)	61.72, 68.49, 83.99	61.05, 68.80, 83.22	61.08, 68.09, 85.08	60.23, 67.68, 84.73	60.91, 69.21, 84.29	60.65, 68.90, 84.08
α, β, γ (°)	90, 90, 90	90, 90, 90	90, 90, 90	90, 90, 90	90, 90, 90	90, 90, 90
**Resolution (Å)**	50.0 – 2.05 (2.15 – 2.05)	50.0 – 1.95 (2.05 – 1.95)	50.0 – 2.35 (2.40 – 2.35)	50.0 – 2.90 (3.00 – 2.90)	50.0 – 2.90 (3.00 – 2.90)	50.0 – 2.90 (3.00 – 2.90)
**R_{meas} (%)**	6.9 (128.1)	5.2 (157.3)	7.1 (94.2)	11.2 (151.1)	18.2 (158.1)	11.0 (144.9)
**I/σI**	19.69 (2.23)	23.53 (1.55)	23.30 (3.21)	15.00 (1.57)	15.61 (2.55)	17.65 (2.18)
**$CC_{1/2}$**	99.9 (79.8)	100 (67.9)	99.9 (88.4)	99.9 (75.7)	99.8 (79.7)	99.9 (81.3)
**Completeness (%)**	100 (100)	99.5 (100)	100 (100)	100 (100)	99.7 (100)	100 (100)
**Redundancy**	13.37 (13.87)	13.00 (12.29)	13.22 (13.91)	12.99 (13.66)	13.13 (12.78)	13.14 (13.84)
**Refinement**						
**Resolution (Å)**	45.47 – 2.05	49.74 – 1.95	45.66 – 2.35	49.09 – 2.90	45.72 – 2.90	49.19 – 2.90
**No. reflections**	22883	26461	15130	8083	8276	8217
**R_{work}/R_{free}**	21.67/24.44	21.12/25.04	20.38/24.45	24.83/27.03	22.34/25.72	22.98/28.58
**No. atoms**						
Protein	2062	2125	2051	2163	2202	2177
Ligand/ion	33	35	39	37	36	34
Water	83	77	40	3	0	1
**B-factors**						
Protein	58.38	60.31	57.61	99.66	72.46	84.97
Ligand/ion	50.21	70.69	60.85	102.79	96.82	105.81
Water	56.71	56.74	61.97	75.15	-	74.77
**rms deviations**						
Bond lengths (Å)	0.009	0.002	0.001	0.002	0.006	0.002
Bond angles (°)	0.945	0.422	0.448	0.520	0.667	0.557
**Wavelength (Å)**	0.9999	0.9999	0.9197	0.9197	0.9197	0.9197
**Temperature (K)**	100	100	100	100	100	100
**X-ray source**	PXII at SLS, Villigen, CH	PXII at SLS, Villigen, CH	PXII at SLS, Villigen, CH	PXII at SLS, Villigen, CH	PXII at SLS, Villigen, CH	PXII at SLS, Villigen, CH
**Detector**	EIGER2 16M	EIGER2 16M	EIGER2 16M	EIGER2 16M	EIGER2 16M	EIGER2 16M
**Ramachandran plot**						
**Residues in**						
favored regions	99.22 %	97.76 %	97.60 %	97.42 %	98.55 %	98.18 %
allowed regions	0.78 %	2.24 %	2.40 %	2.21 %	1.09 %	1.82 %
outlier regions	0.00 %	0.00 %	0.00 %	0.37 %	0.36 %	0.00 %

Table S1. Crystallographic statistics of MKK7 in complex with triazolyl inhibitor derivatives. Statistics for co-crystals with compounds **1a**, **1h**, **1k**, **2a**, **2b**, **2c** (PDB: 7OVI, 7OVJ, 7OVK, 7OVL, 7OVM, 7OVN). Values in parenthesis refer to the highest resolution shell.

HPLC and NMR spectra of reported compounds

(*R*)-1-(3-(4-amino-3-(1-(4-bromo-2-hydroxyphenyl)-1*H*-1,2,3-triazol-4-yl)-1*H*-pyrazolo[3,4-*d*]pyrimidin-1-yl)piperidin-1-yl)prop-2-en-1-one (1a)

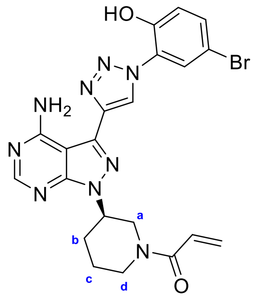

LC-MS chromatogram, detection at 280 nm

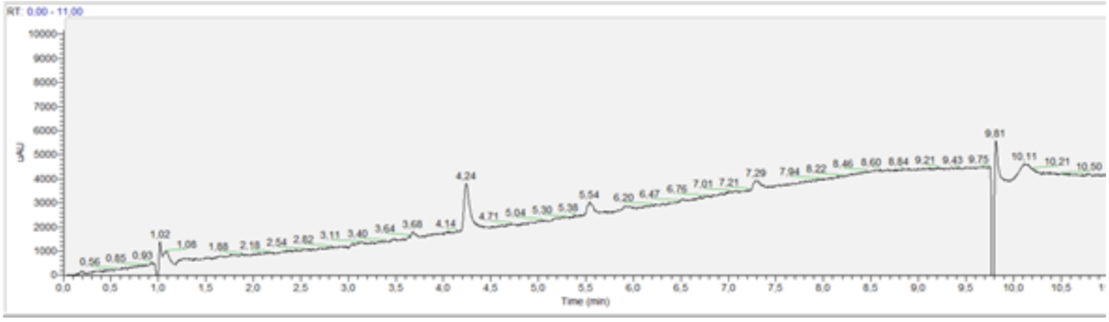

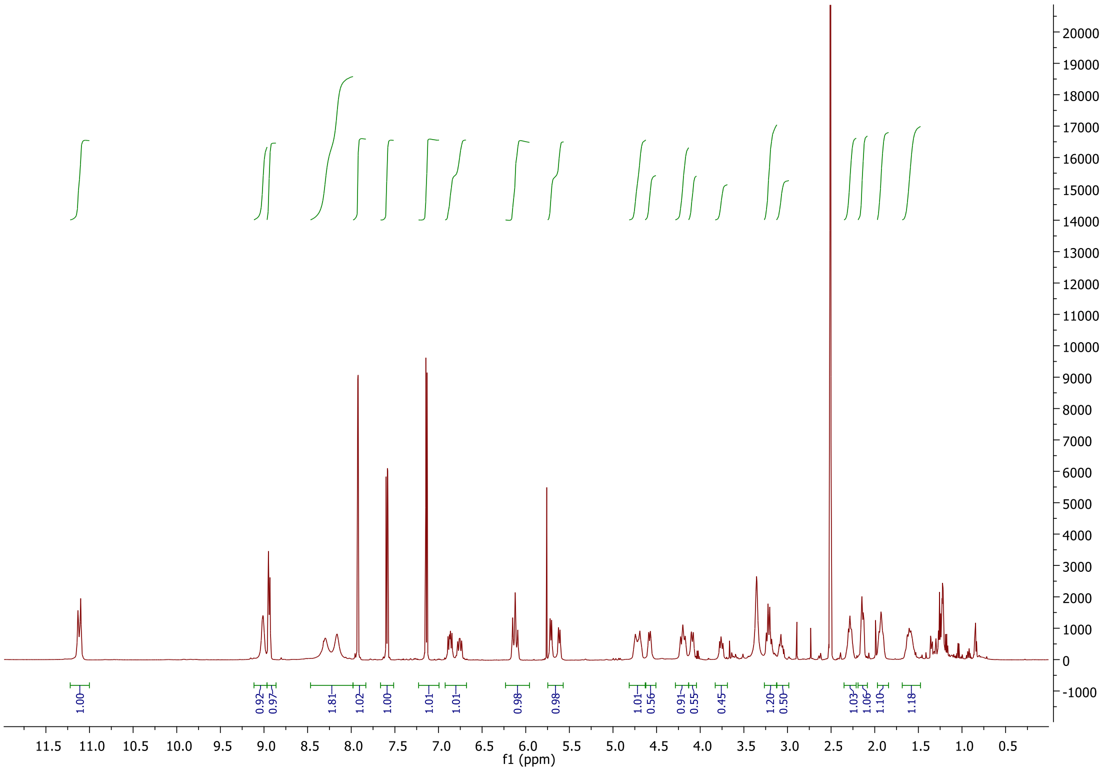
1.00
0.92
0.97
1.81
1.02
1.00
1.01
1.01
0.98
0.98
1.01
0.56
0.91
0.55
0.45
1.20
0.50
1.03
1.06
1.10
1.18
f1 (ppm)

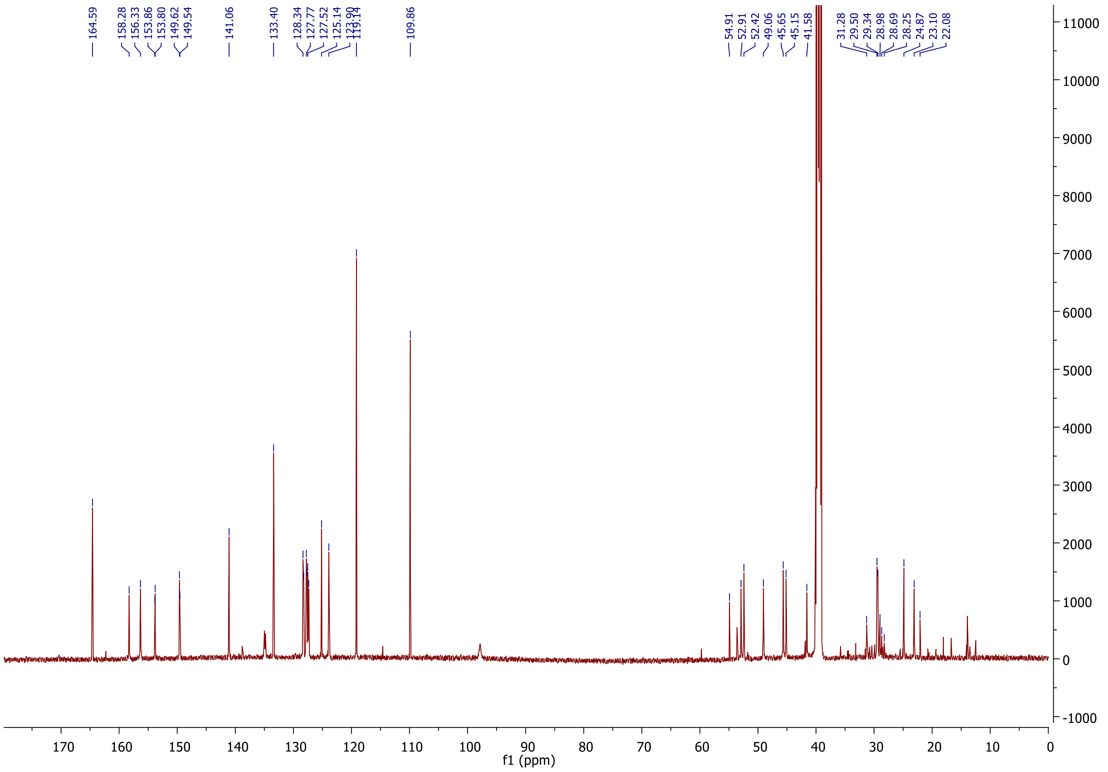
164.59
158.28
156.33
153.86
153.80
149.62
149.54
141.06
133.40
128.34
127.77
127.52
125.14
123.90
119.14
109.86
54.91
52.91
52.42
49.06
45.65
45.15
41.58
31.28
29.50
29.34
28.98
28.69
28.25
24.87
23.10
22.08
f1 (ppm)

(*R*)-1-(3-(4-amino-3-(1-(2-(4-chlorophenyl)-2,2-difluoroethyl)-1*H*-1,2,3-triazol-4-yl)-1*H*-pyrazolo[3,4-*d*]pyrimidin-1-yl)piperidin-1-yl)prop-2-en-1-one (1b)

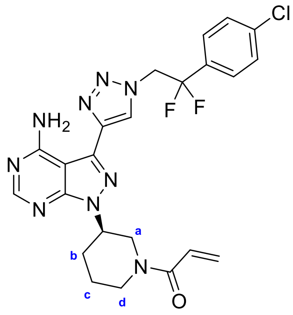

LC-MS chromatogram, detection at 280 nm

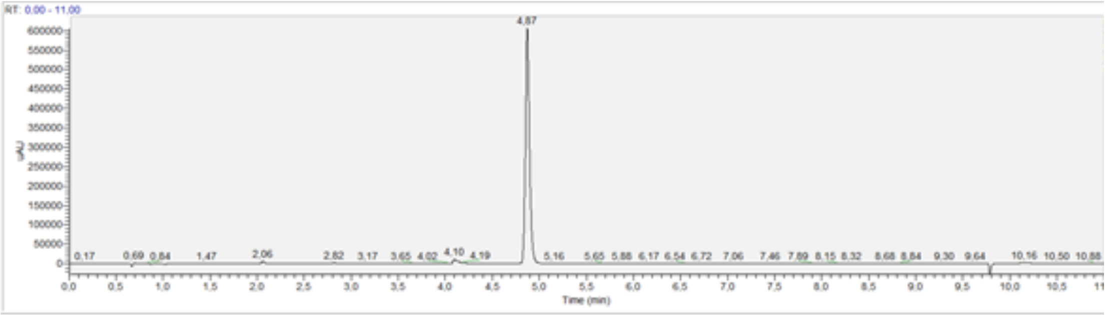

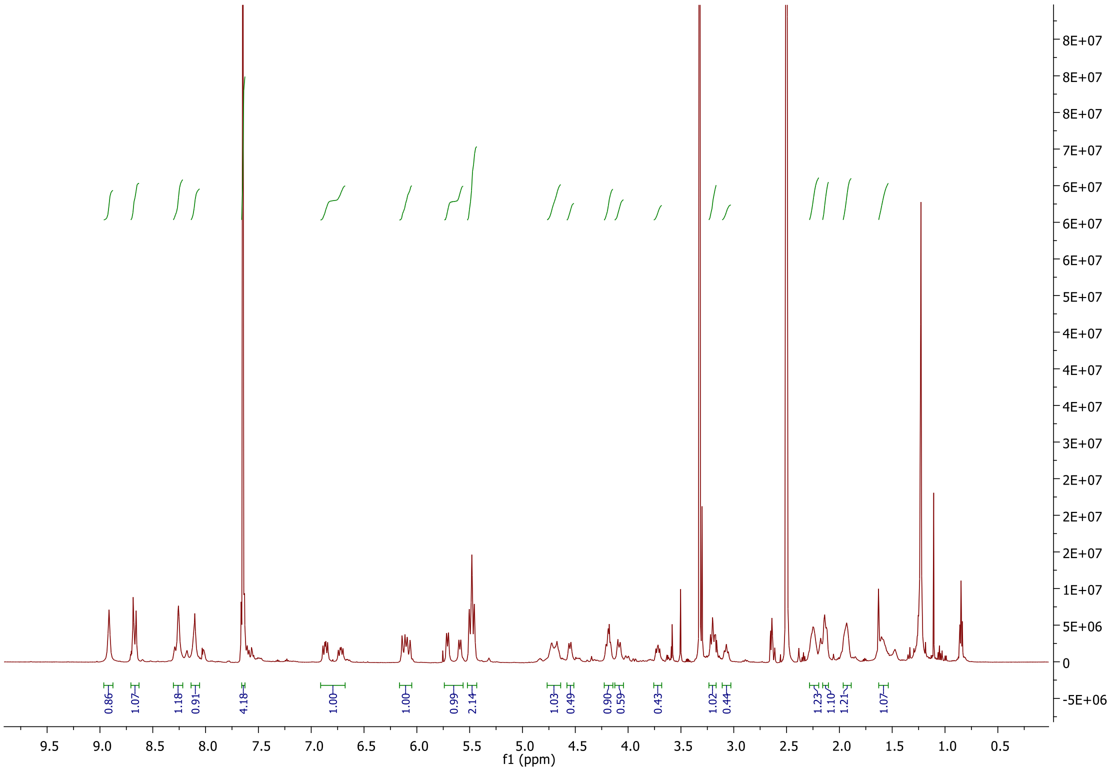

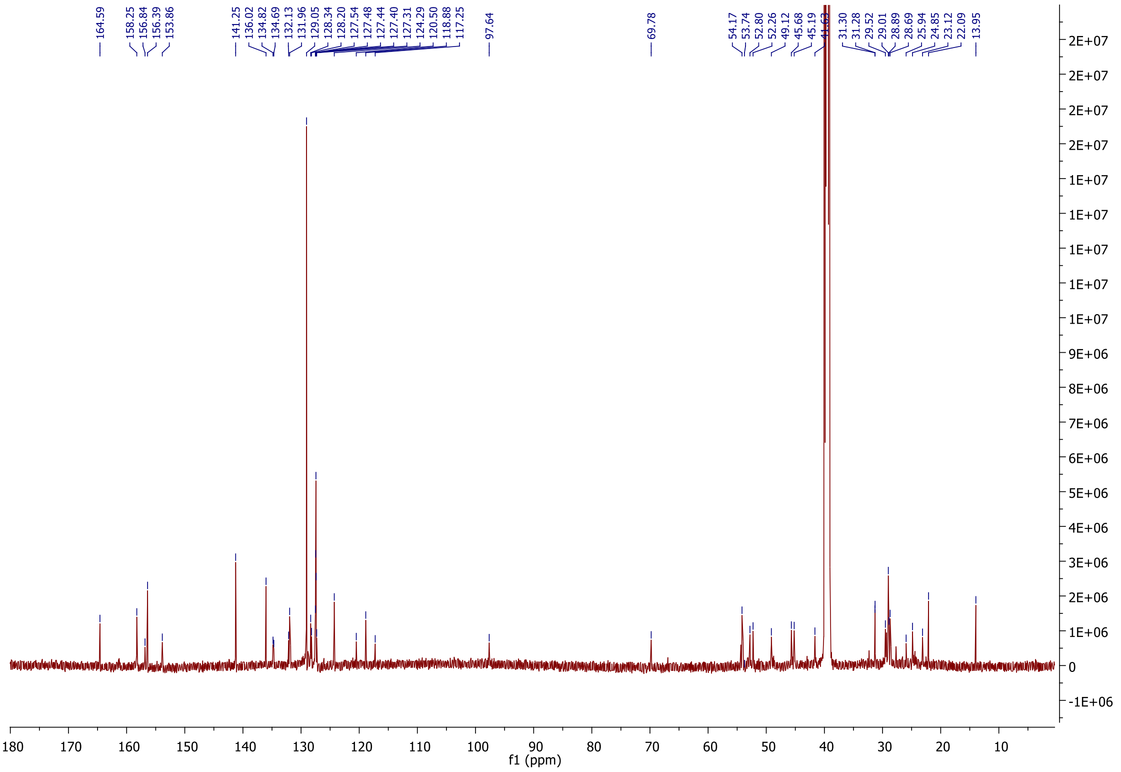

(*R*)-1-(3-(4-amino-3-(1-(3-fluoro-4-(hydroxymethyl)phenyl)-1*H*-1,2,3-triazol-4-yl)-1*H*-pyrazolo[3,4-*d*]pyrimidin-1-yl)piperidin-1-yl)prop-2-en-1-one (1c)

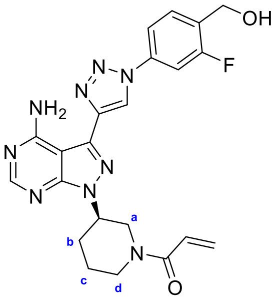

LC-MS chromatogram, detection at 280 nm

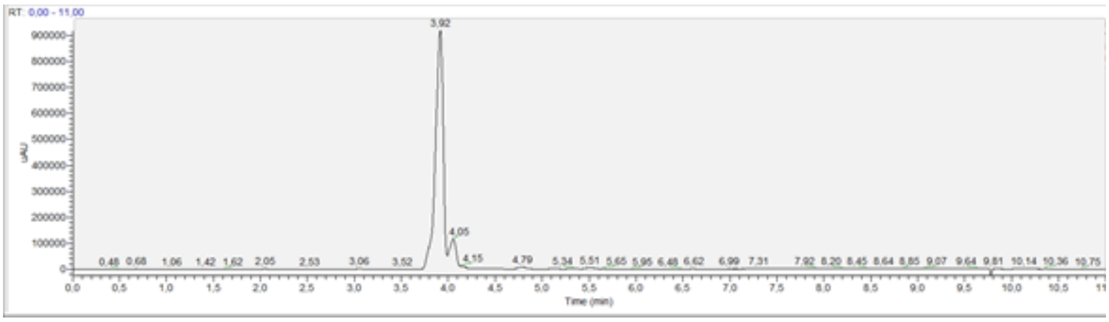

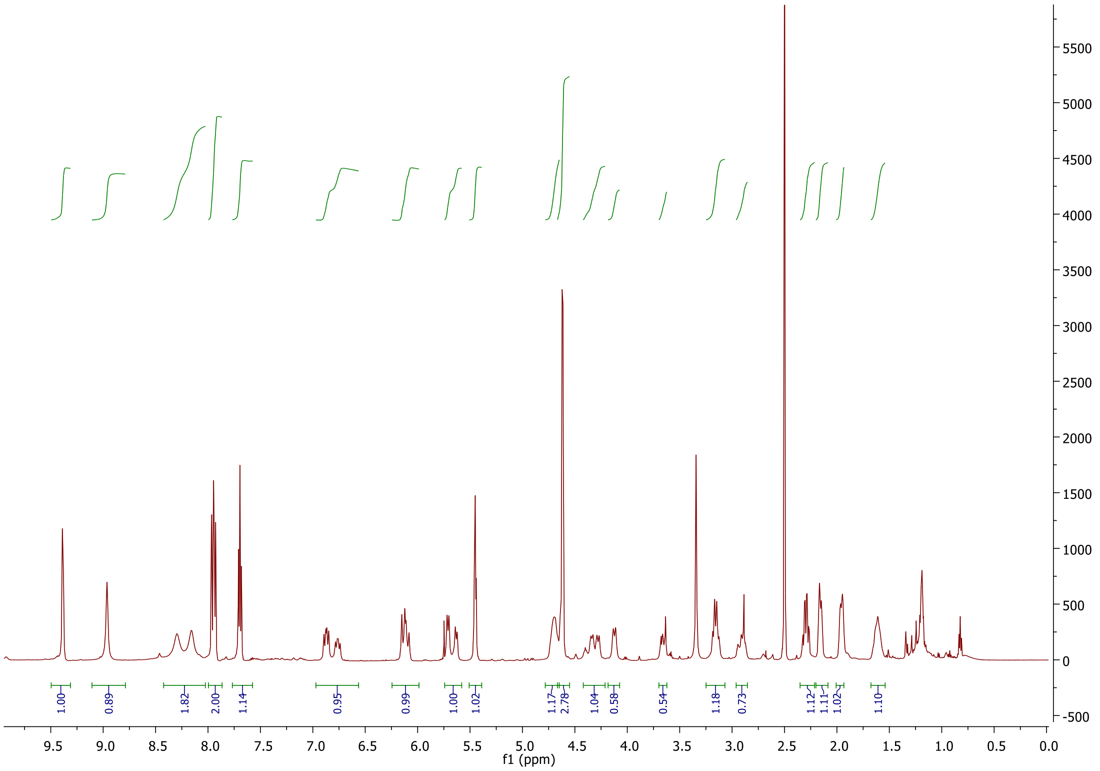
1.00
0.89
1.82
2.00
1.14
0.95
0.99
1.00
1.02
1.17
2.78
1.04
0.58
0.54
1.18
0.73
1.12
1.11
1.02
1.10
f1 (ppm)

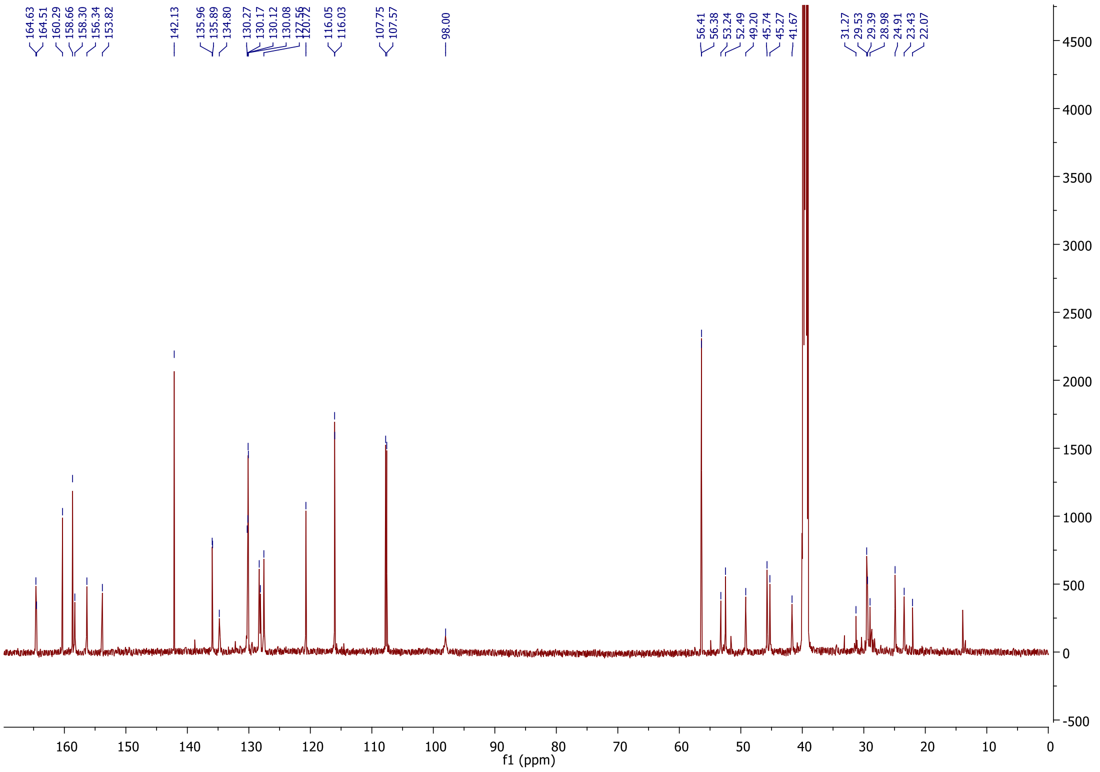
164.63
164.51
160.29
158.66
158.30
156.34
153.82
142.13
135.96
135.89
134.80
130.27
130.17
130.12
130.08
127.56
120.72
116.05
116.03
107.75
107.57
98.00
56.41
56.38
53.24
52.49
49.20
45.74
45.27
41.67
31.27
29.53
29.39
28.98
24.91
23.43
22.07
f1 (ppm)

(*R*)-1-(3-(4-amino-3-(1-(4-chloro-2-hydroxyphenyl)-1*H*-1,2,3-triazol-4-yl)-1*H*-pyrazolo[3,4-*d*]pyrimidin-1-yl)piperidin-1-yl)prop-2-en-1-one (1d)

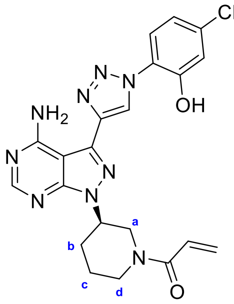

LC-MS chromatogram, detection at 280 nm

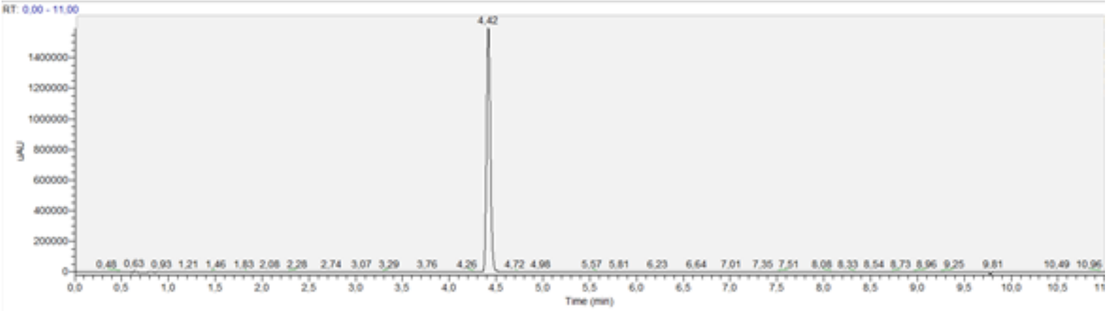

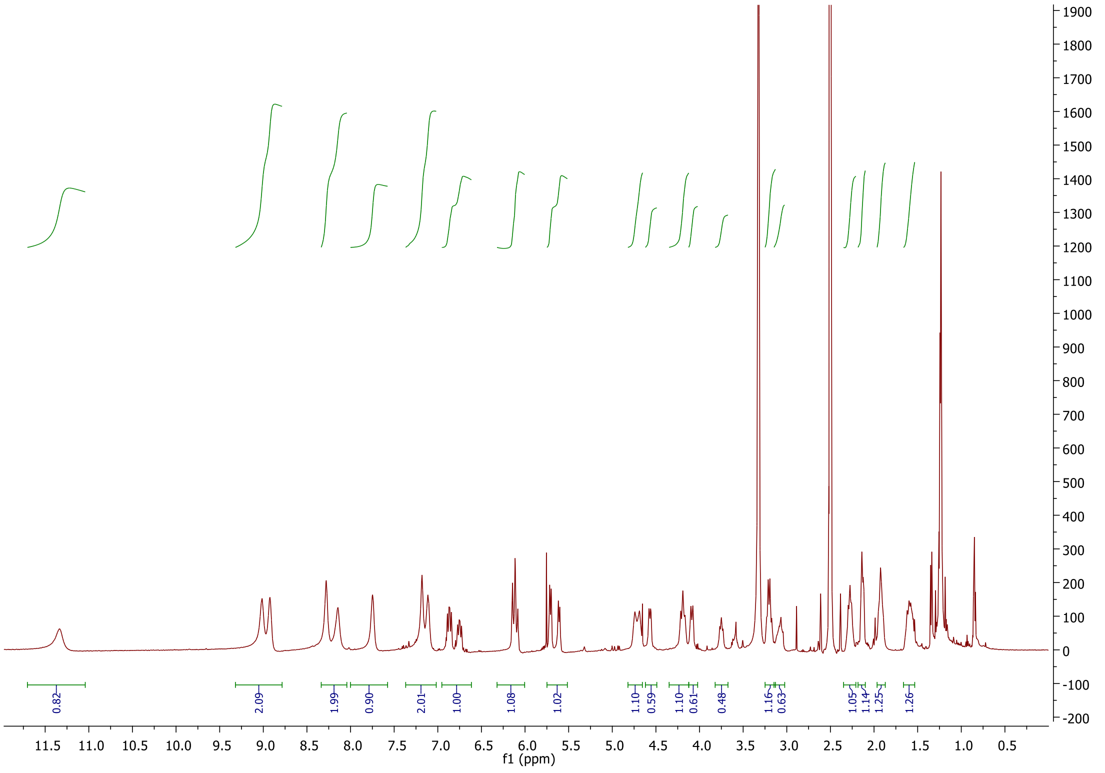
0.82
2.09
1.99
0.90
2.01
1.00
1.08
1.02
1.10
0.59
1.10
0.61
0.48
1.16
0.63
1.05
1.14
1.25
1.26
f1 (ppm)

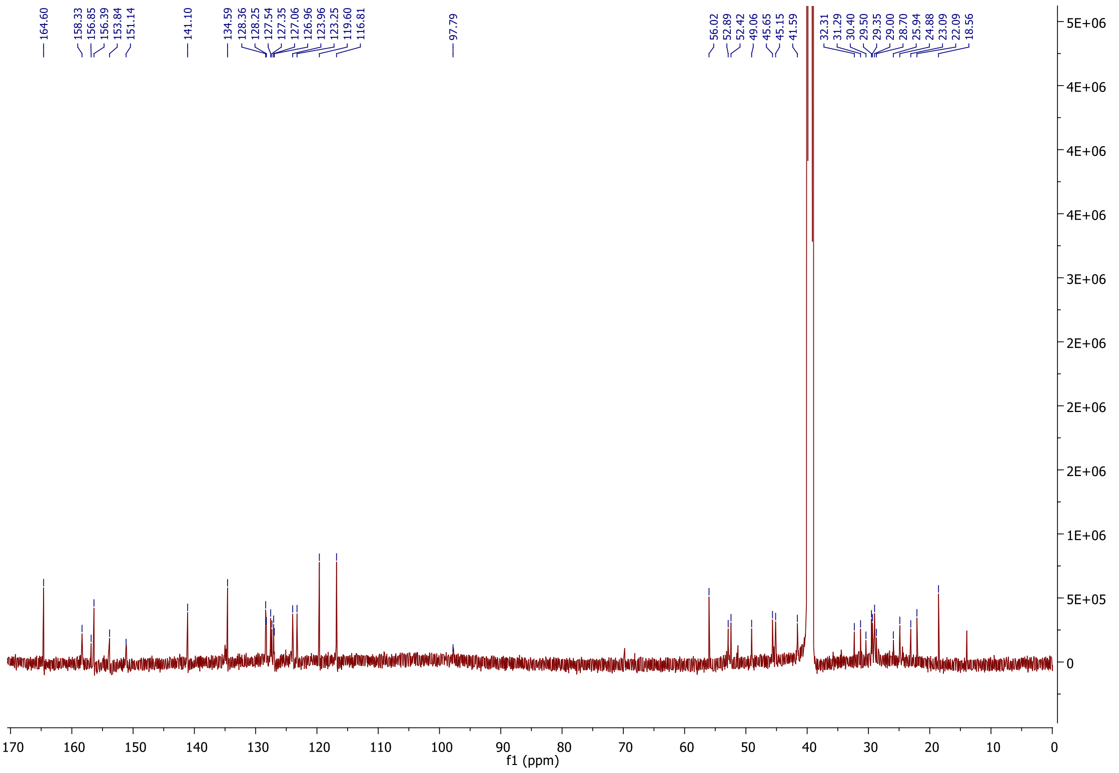
164.60
158.33
156.85
156.39
153.84
151.14
141.10
134.59
128.36
128.25
127.54
127.35
127.06
126.96
123.96
123.25
119.60
116.81
97.79
56.02
52.89
52.42
49.06
45.65
45.15
41.59
32.31
31.29
30.40
29.50
29.35
29.00
28.70
25.94
24.88
23.09
22.09
18.56
f1 (ppm)

1-((*R*)-3-(4-amino-3-(1-((*R*)-2-(4-bromophenyl)-2-hydroxy-ethyl)-1*H*-1,2,3-triazol-4-yl)-1*H*-pyrazolo[3,4-*d*]pyrimidin-1-yl)piperidin-1-yl)prop-2-en-1-one (1e)

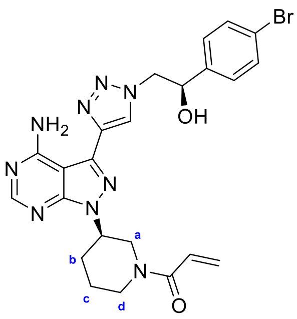

LC-MS chromatogram, detection at 280 nm

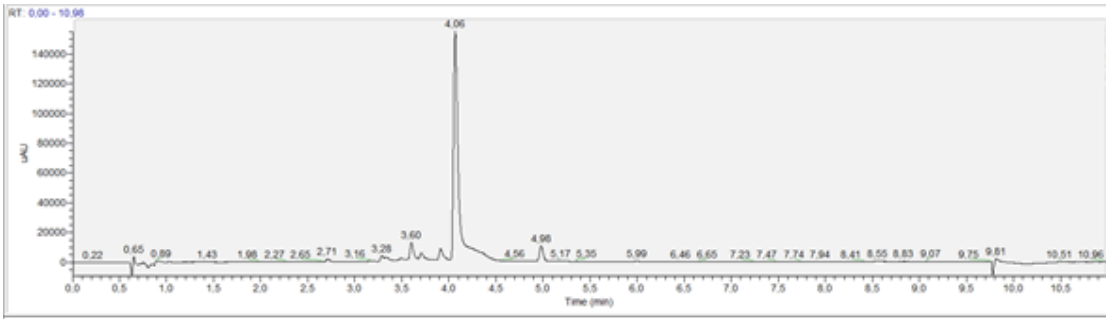

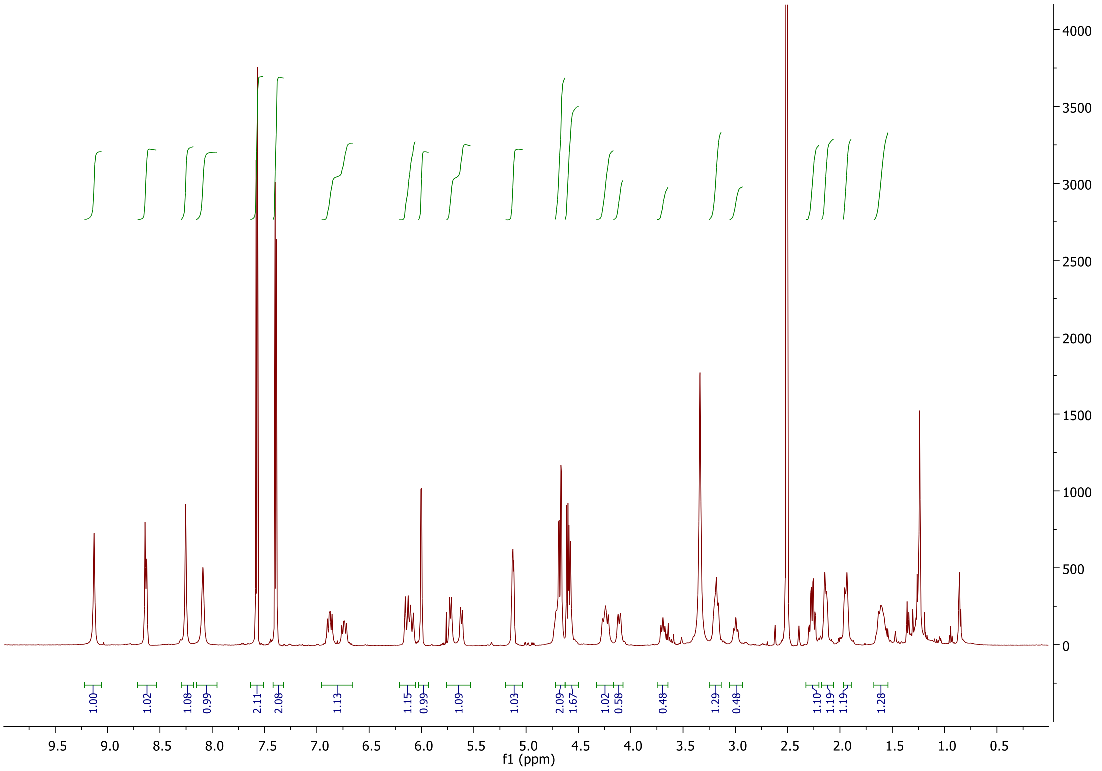
1.00
1.02
1.08
0.99
2.11
2.08
1.13
1.15
0.99
1.09
1.03
2.09
1.67
1.02
0.58
0.48
1.29
0.48
1.10
1.19
1.19
1.28
f1 (ppm)

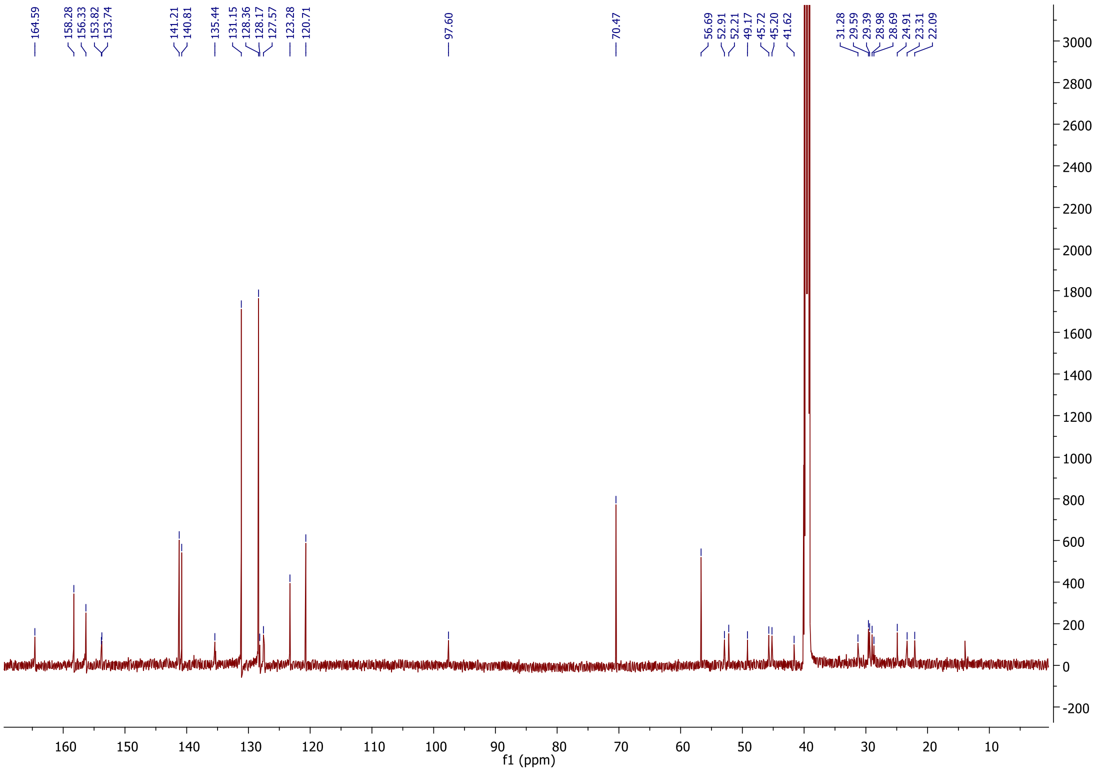
164.59
158.28
156.33
153.82
153.74
141.21
140.81
135.44
131.15
128.36
128.17
127.57
123.28
120.71
97.60
70.47
56.69
52.91
52.21
49.17
45.72
45.20
41.62
31.28
29.59
29.39
28.98
28.69
24.91
23.31
22.09
f1 (ppm)

(*R*)-1-(3-(4-amino-3-(1-(4-bromophenyl)-1H-1,2,3-triazol-4-yl)-1*H*-pyrazolo[3,4-*d*]pyrimidin-1-yl)piperidin-1-yl)prop-2-en-1-one (1f)

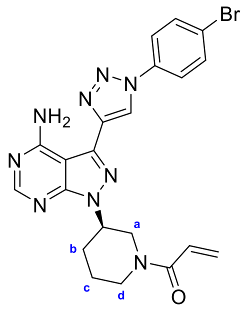

LC-MS chromatogram, detection at 280 nm

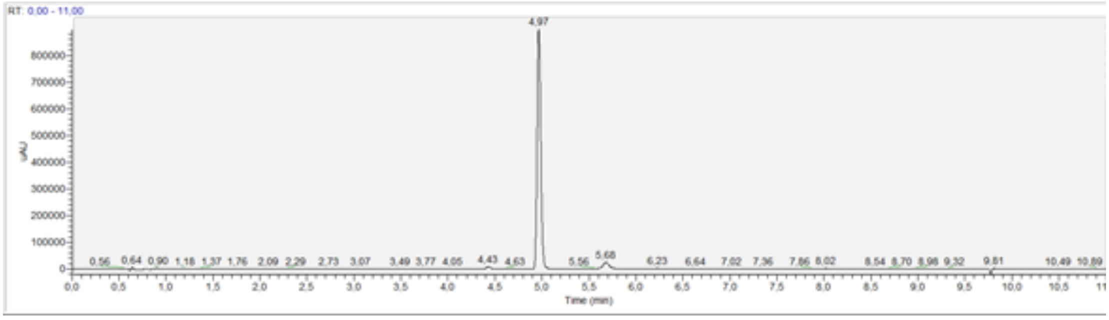

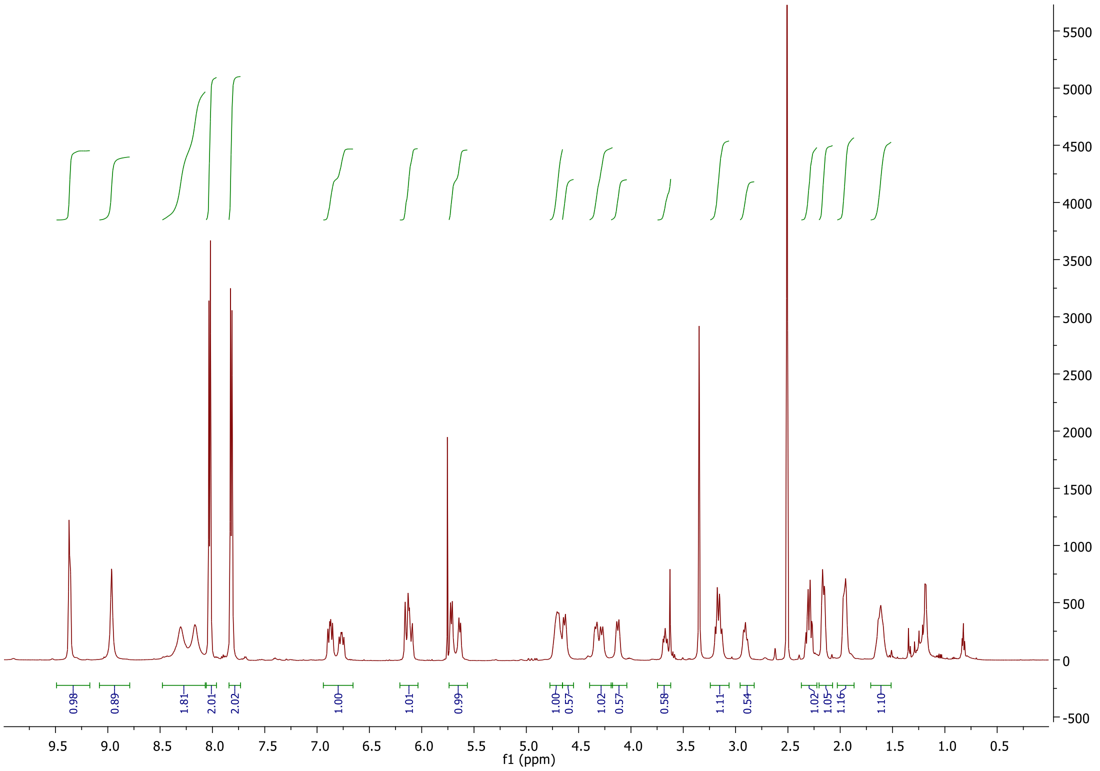
0.98
0.89
1.81
2.01
2.02
1.00
1.01
0.99
1.00
0.57
1.02
0.57
0.58
1.11
0.54
1.02
1.05
1.16
1.10
f1 (ppm)

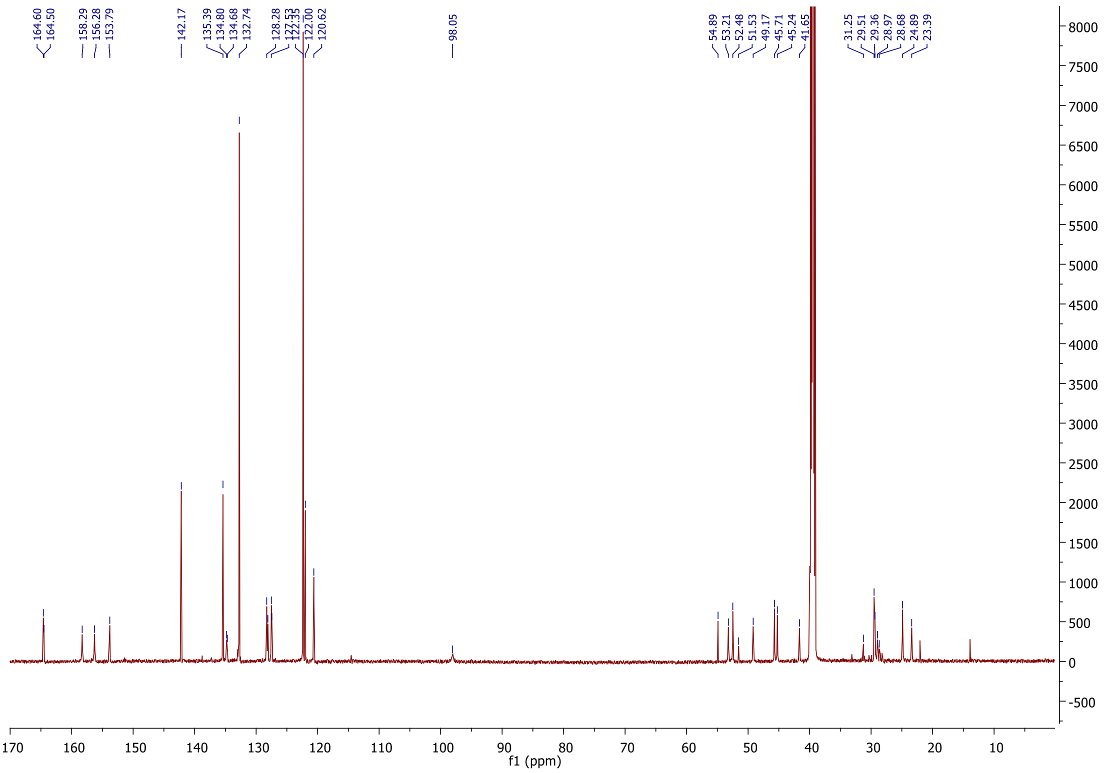
164.60
164.50
158.29
156.28
153.79
142.17
135.39
134.80
134.68
132.74
128.28
127.53
122.35
122.00
120.62
98.05
54.89
53.21
52.48
51.53
49.17
45.71
45.24
41.65
31.25
29.51
29.36
28.97
28.68
24.89
23.39
f1 (ppm)

(*R*)-1-(3-(4-amino-3-(1-phenethyl-1*H*-1,2,3-triazol-4-yl)-1*H*-pyrazolo[3,4-*d*]pyrimidin-1-yl)piperidin-1-yl)prop-2-en-1-one (1h)

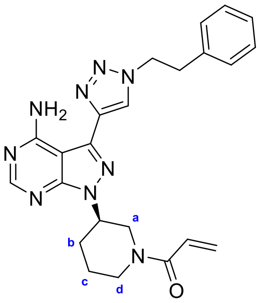

LC-MS chromatogram, detection at 280 nm

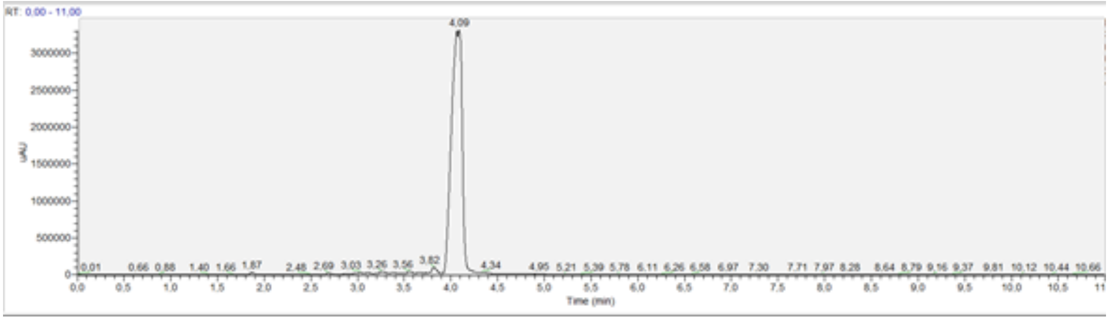

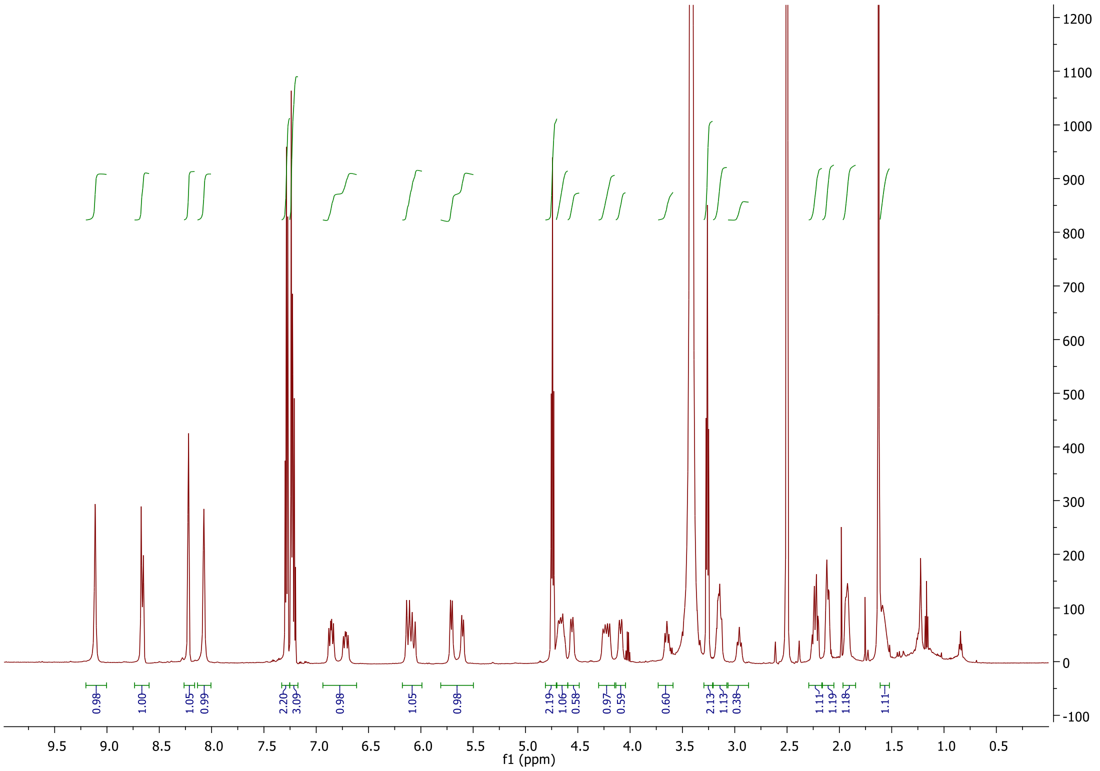
0.98
1.00
1.05
0.99
2.20
3.09
0.98
1.05
0.98
2.19
1.06
0.58
0.97
0.59
0.60
2.13
1.13
0.38
1.11
1.19
1.18
1.11
f1 (ppm)

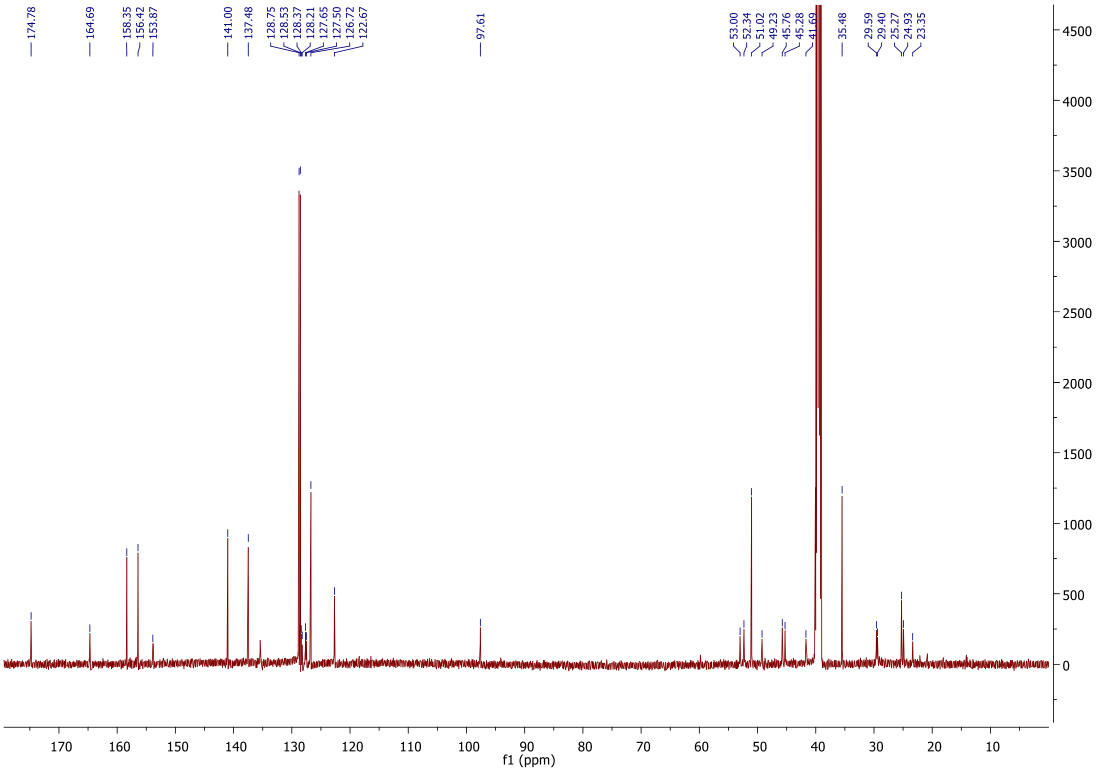
174.78
164.69
158.35
156.42
153.87
141.00
137.48
128.75
128.53
128.37
128.21
127.65
127.50
126.72
122.67
97.61
53.00
52.34
51.02
49.23
45.76
45.28
41.69
35.48
29.59
29.40
25.27
24.93
23.35
f1 (ppm)

(*R*)-1-(3-(4-Amino-3-(1-(4-methoxyphenyl)-1*H*-1,2,3-triazol-4-yl)-1*H*-pyrazolo[3,4-*d*]pyrimidin-1-yl)piperidin-1-yl)prop-2-en-1-one (1i)

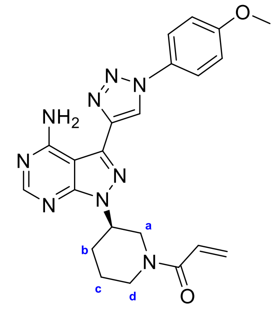

LC-MS chromatogram, detection at 280 nm

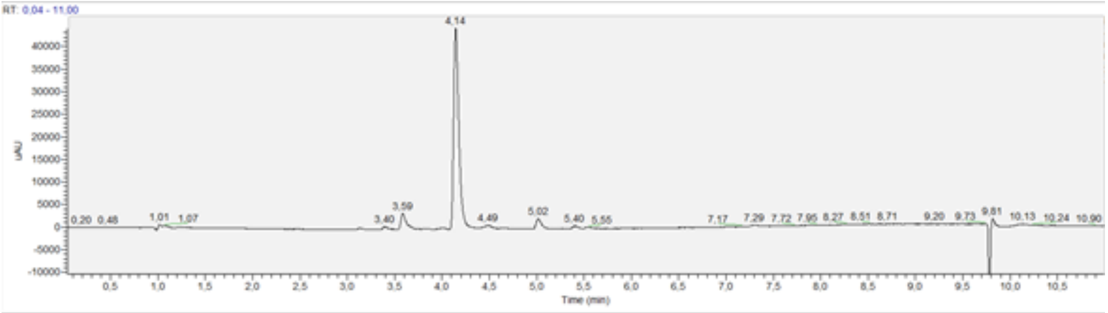

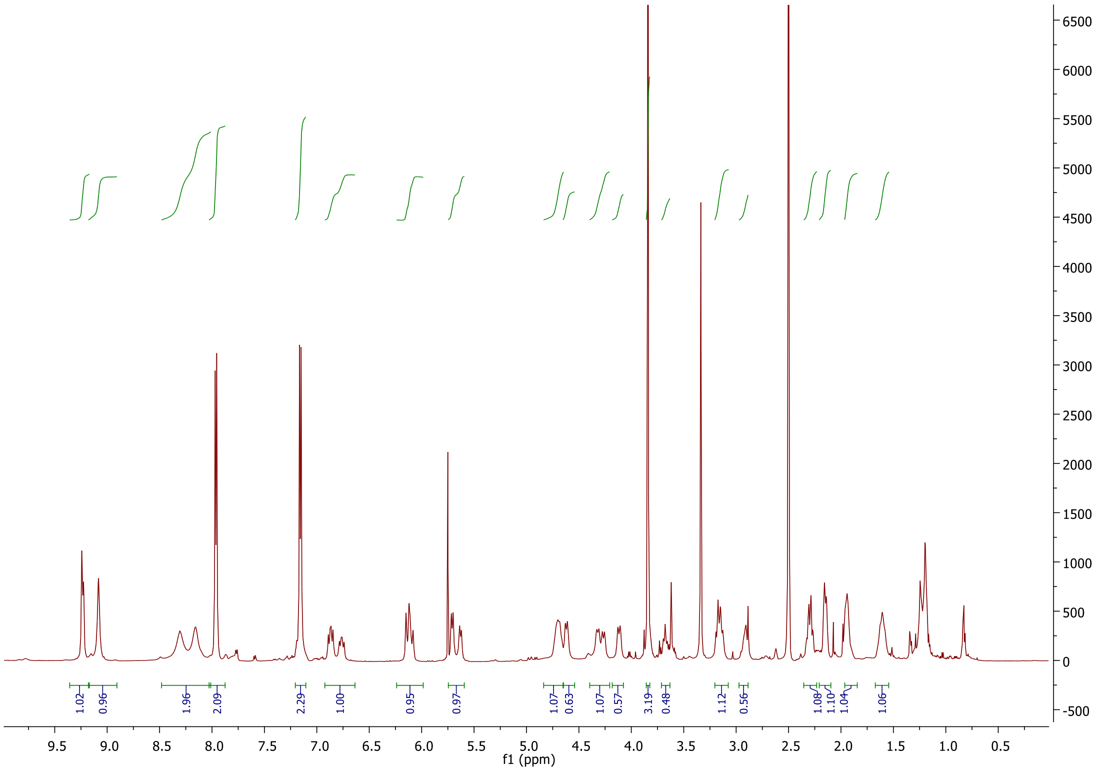
1.02
0.96
1.96
2.09
2.29
1.00
0.95
0.97
1.07
0.63
1.07
0.57
3.19
0.48
1.12
0.56
1.08
1.10
1.04
1.06
f1 (ppm)

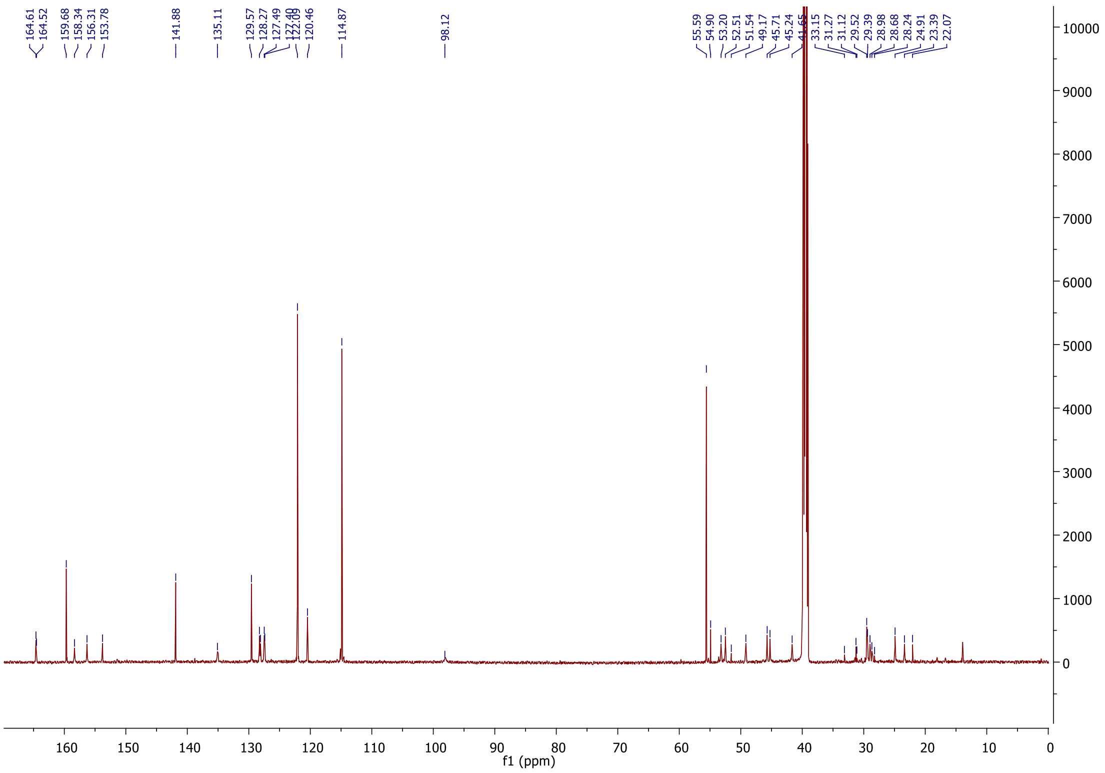
164.61
164.52
159.68
158.34
156.31
153.78
141.88
135.11
129.57
128.27
127.49
122.09
120.46
114.87
98.12
55.59
54.90
53.20
52.51
51.54
49.17
45.71
45.24
33.15
31.27
31.12
29.52
29.39
28.98
28.68
28.24
24.91
23.39
22.07
f1 (ppm)

Methyl (*R*)-4-(4-(1-(1-acryloylpiperidin-3-yl)-4-amino-1*H*-pyrazolo [3,4-*d*]pyrimidin-3-yl)-1H-1,2,3-triazol-1-yl)benzoate (1j)

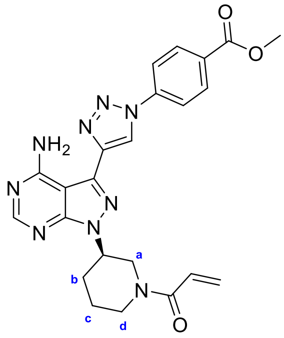

LC-MS chromatogram, detection at 280 nm

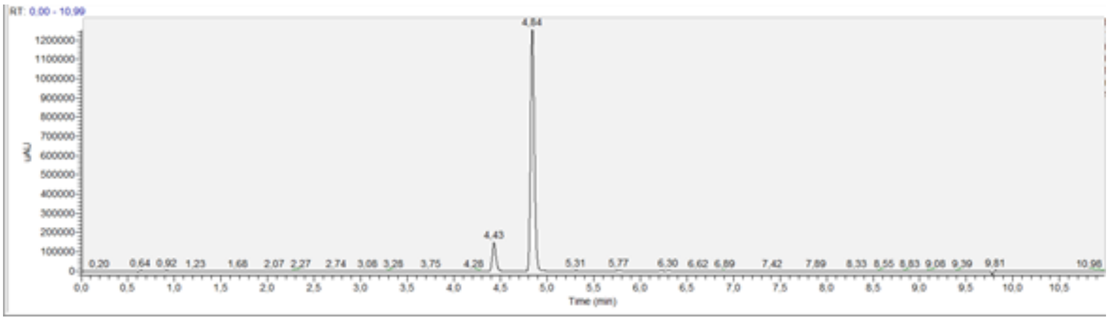

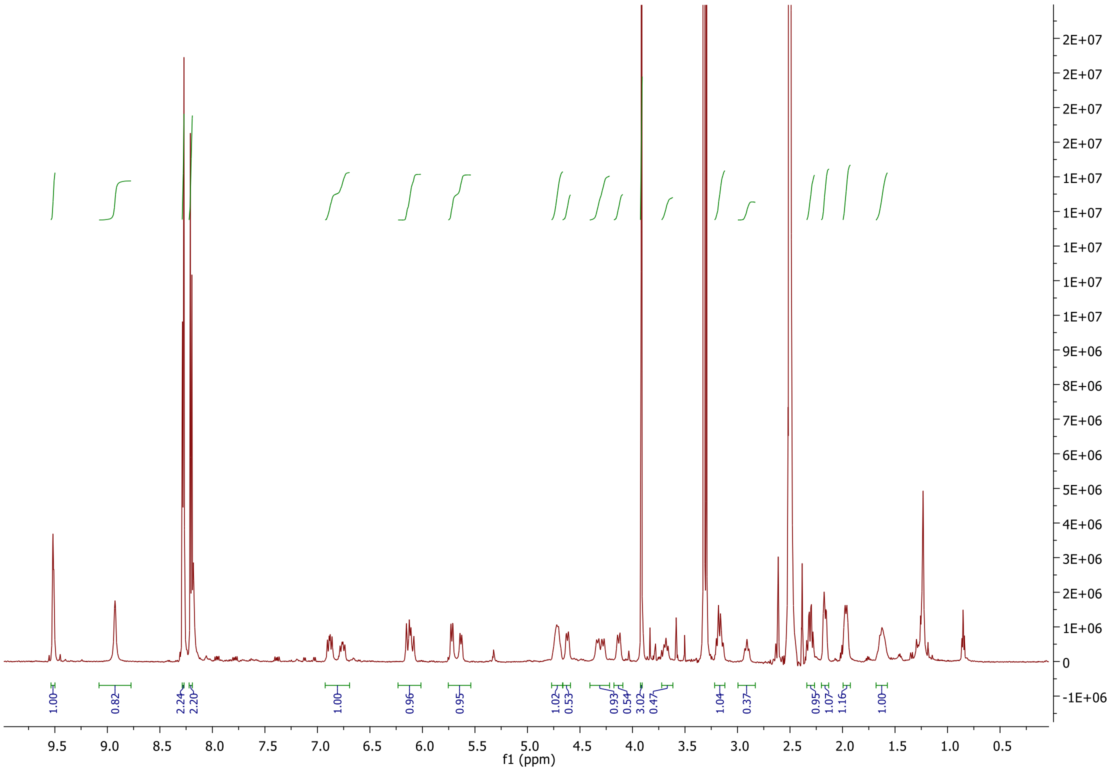

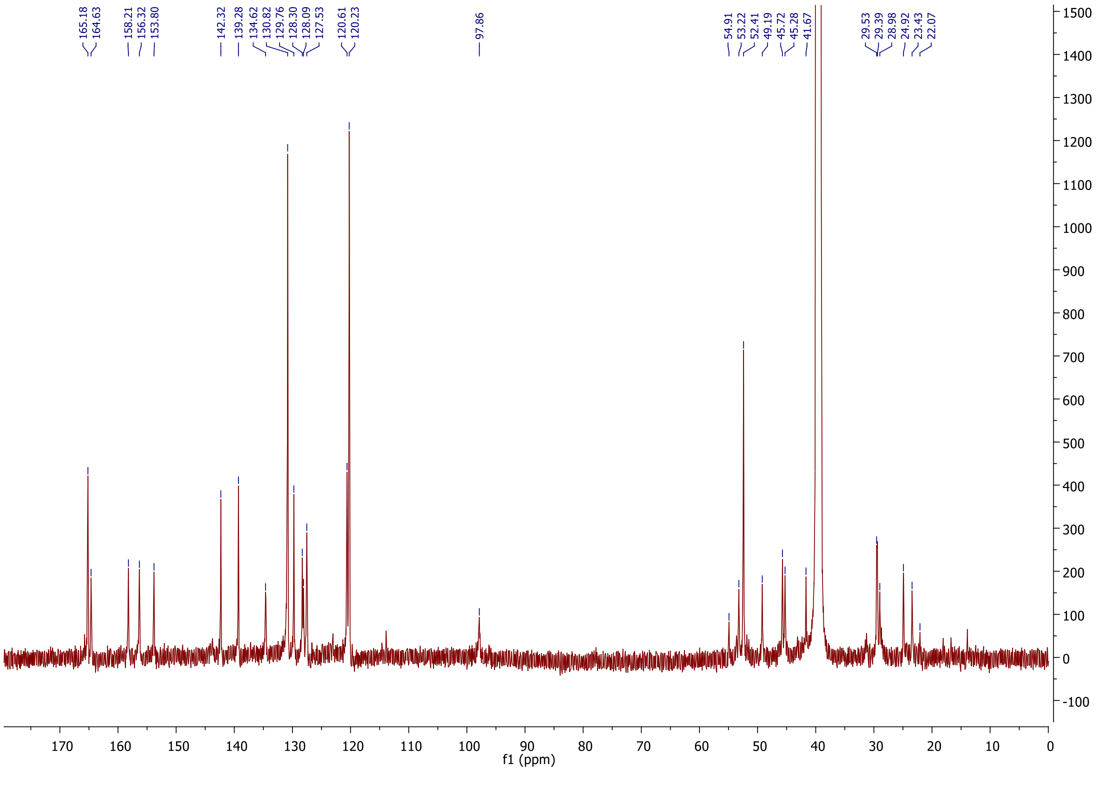

(*R*)-1-(3-(4-amino-3-(1-(2,2-difluoro-2-phenylethyl)-1*H*-1,2,3-triazol-4-yl)-1*H*-pyrazolo[3,4-*d*]pyrimidin-1-yl)piperidin-1-yl)prop-2-en-1-one (1k)

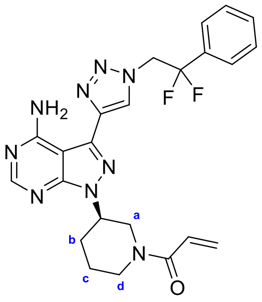

LC-MS chromatogram, detection at 280 nm

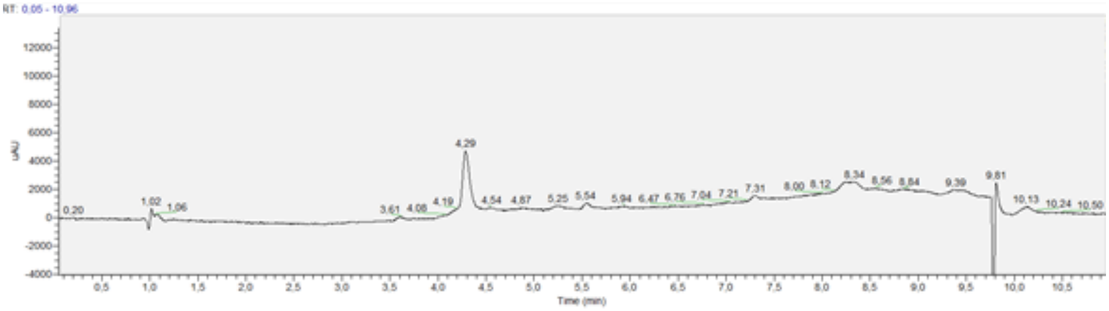

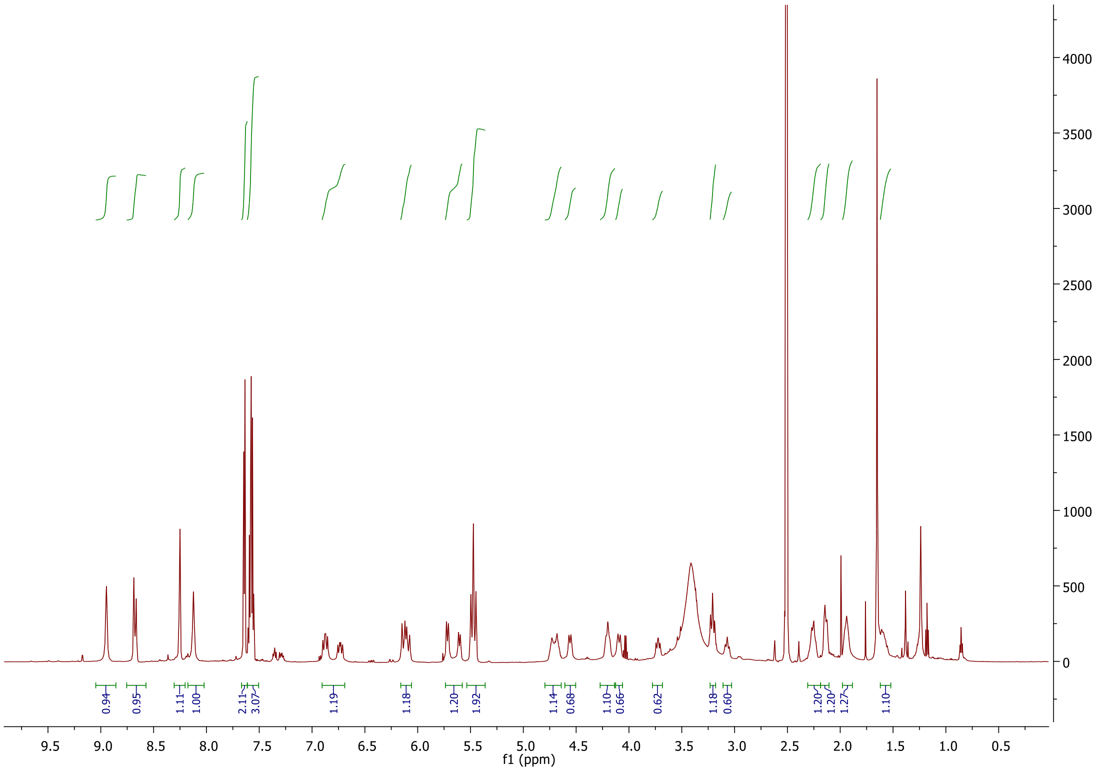
0.94
0.95
1.11
1.00
2.11
3.07
1.19
1.18
1.20
1.92
1.14
0.68
1.10
0.66
0.62
1.18
0.60
1.20
1.20
1.27
1.10
f1 (ppm)

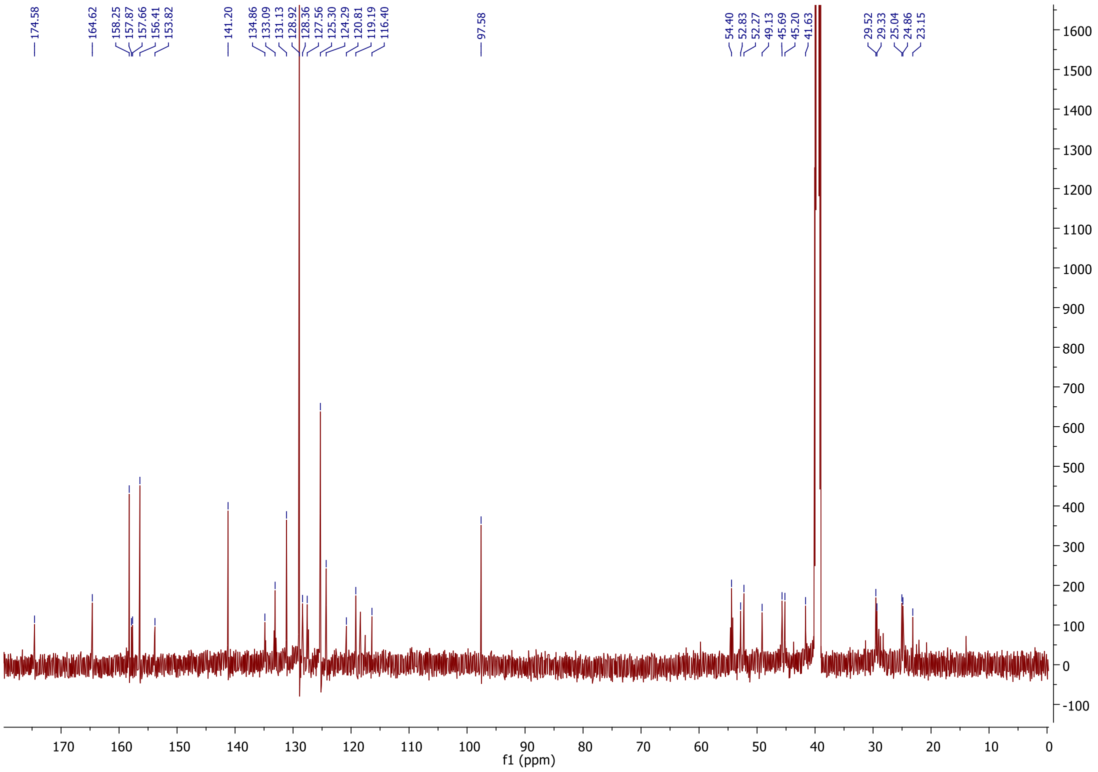
174.58
164.62
158.25
157.87
157.66
156.41
153.82
141.20
134.86
133.09
131.13
128.92
128.36
127.56
125.30
124.29
120.81
119.19
116.40
97.58
54.40
52.83
52.27
49.13
45.69
45.20
41.63
29.52
29.33
25.04
24.86
23.15
f1 (ppm)

<u>Synthesis of IB-1-24*:*</u>

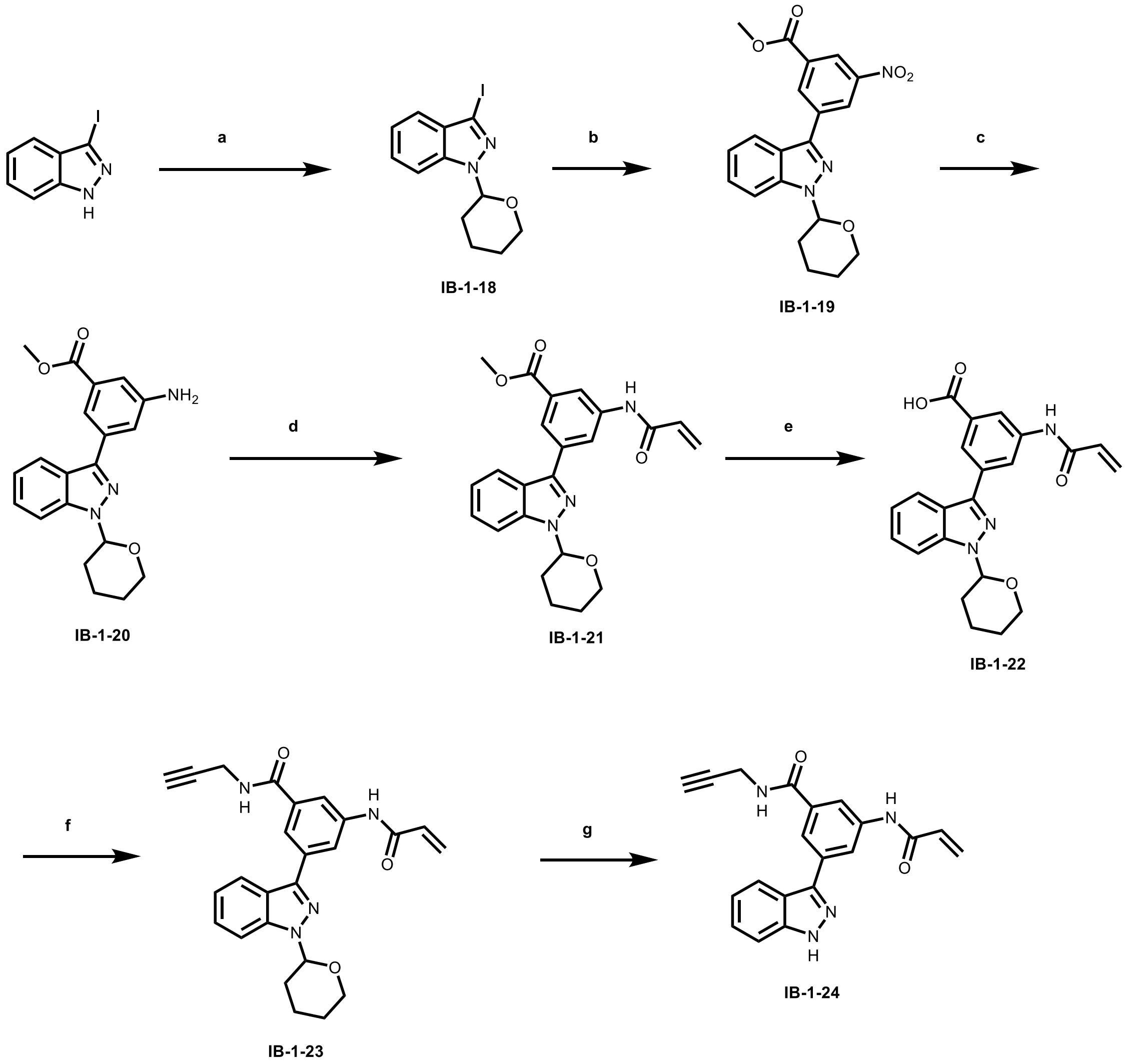

3-iodo-1-(tetrahydro-2H-pyran-2-yl)-1H-indazole (IB-1-18)

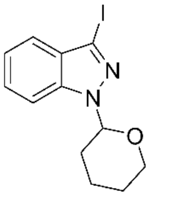

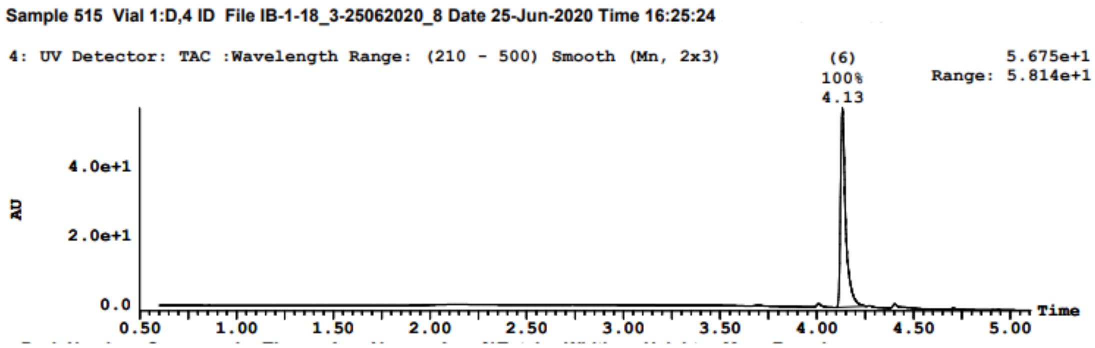
Sample 515 Vial 1:D,4 ID File IB-1-18_3-25062020_8 Date 25-Jun-2020 Time 16:25:24
4: UV Detector: TAC :Wavelength Range: (210 - 500) Smooth (Mn, 2x3)
(6)
100%
4.13
5.675e+1
Range: 5.814e+1
AU
4.0e+1
2.0e+1
0.0
0.50
1.00
1.50
2.00
2.50
3.00
3.50
4.00
4.50
5.00
Time

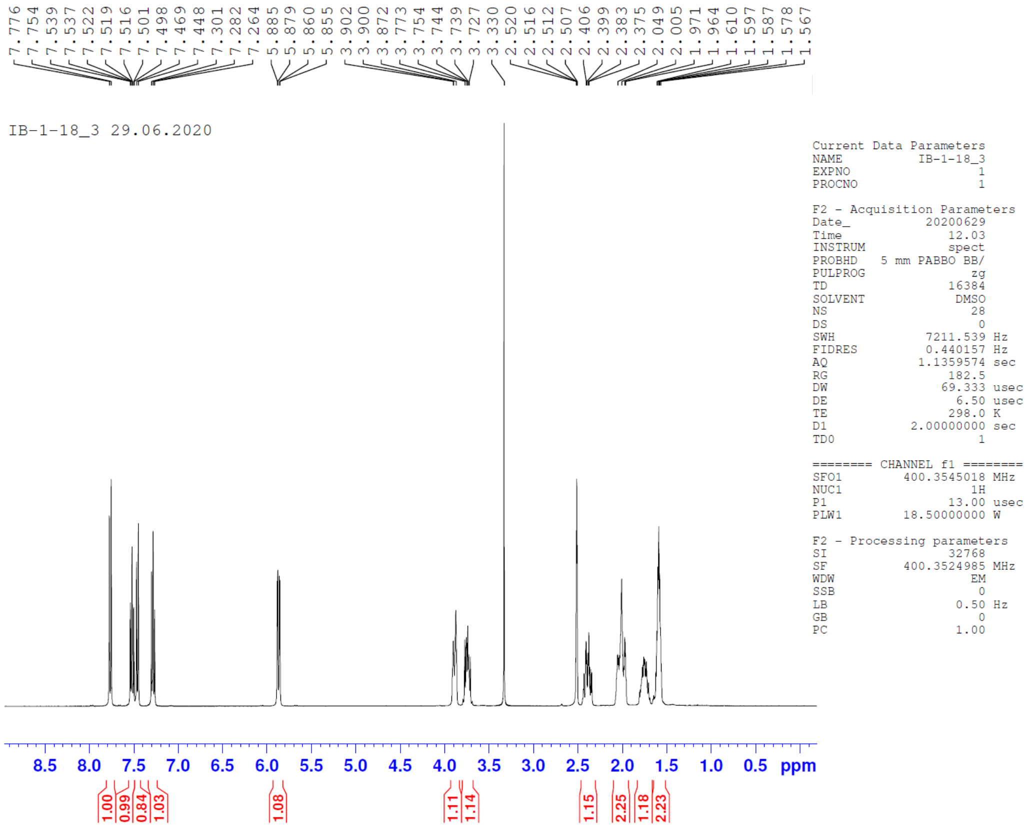
7.776
7.754
7.539
7.537
7.522
7.519
7.516
7.501
7.498
7.469
7.448
7.301
7.282
7.264
5.885
5.879
5.860
5.855
3.902
3.900
3.872
3.773
3.754
3.744
3.739
3.727
3.330
2.520
2.516
2.512
2.507
2.406
2.399
2.383
2.375
2.049
2.005
1.971
1.964
1.610
1.597
1.587
1.578
1.567
IB-1-18_3 29.06.2020
Current Data Parameters
NAME IB-1-18_3
EXPNO 1
PROCNO 1
F2 - Acquisition Parameters
Date_ 20200629
Time 12.03
INSTRUM spect
PROBHD 5 mm PABBO BB/
PULPROG zg
TD 16384
SOLVENT DMSO
NS 28
DS 0
SWH 7211.539 Hz
FIDRES 0.440157 Hz
AQ 1.1359574 sec
RG 182.5
DW 69.333 usec
DE 6.50 usec
TE 298.0 K
D1 2.00000000 sec
TD0 1
======== CHANNEL f1 ========
SFO1 400.3545018 MHz
NUC1 1H
P1 13.00 usec
PLW1 18.50000000 W
F2 - Processing parameters
SI 32768
SF 400.3524985 MHz
WDW EM
SSB 0
LB 0.50 Hz
GB 0
PC 1.00
8.5
8.0
7.5
7.0
6.5
6.0
5.5
5.0
4.5
4.0
3.5
3.0
2.5
2.0
1.5
1.0
0.5
ppm
1.00
0.99
0.84
1.03
1.08
1.11
1.14
1.15
2.25
1.18
2.23

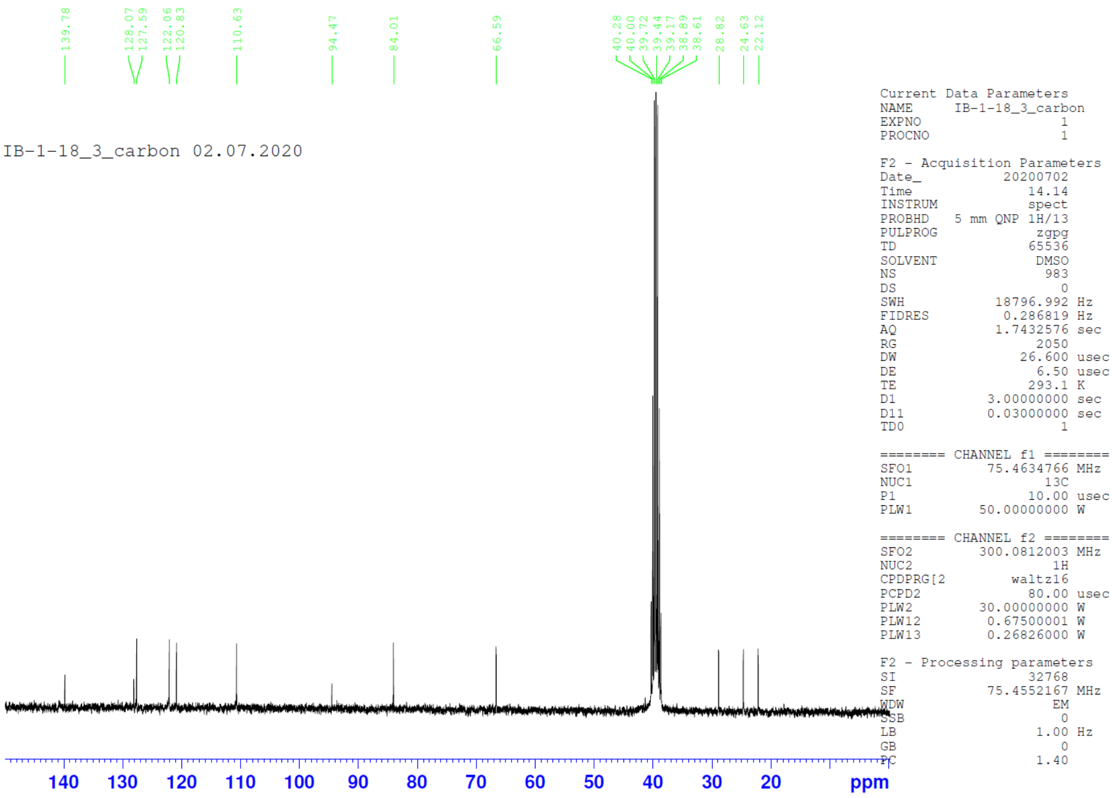

Methyl 3-nitro-5-(1-(tetrahydro-2H-pyran-2-yl)-1H-indazol-3-yl)benzoate (IB-1-19).

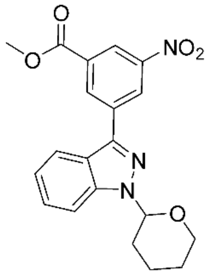

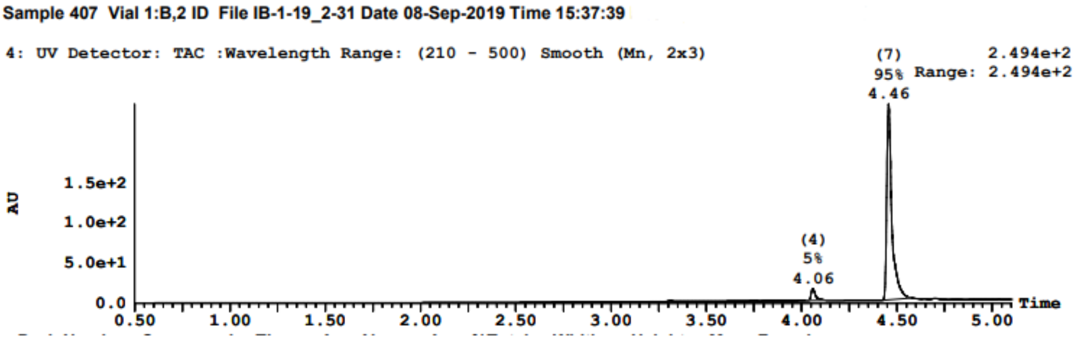

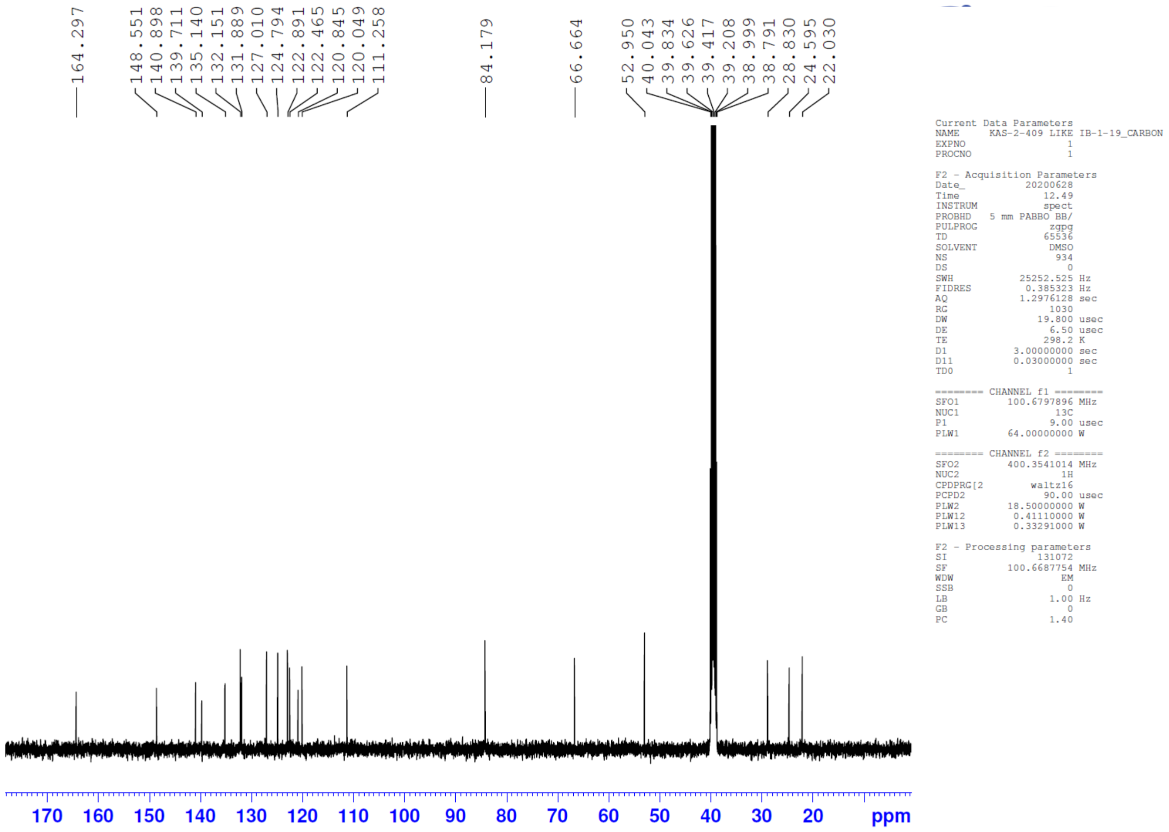

Methyl 3-amino-5-(1-(tetrahydro-2H-pyran-2-yl)-1H-indazol-3-yl)benzoate (IB-1-20).

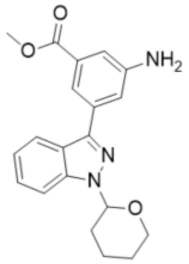

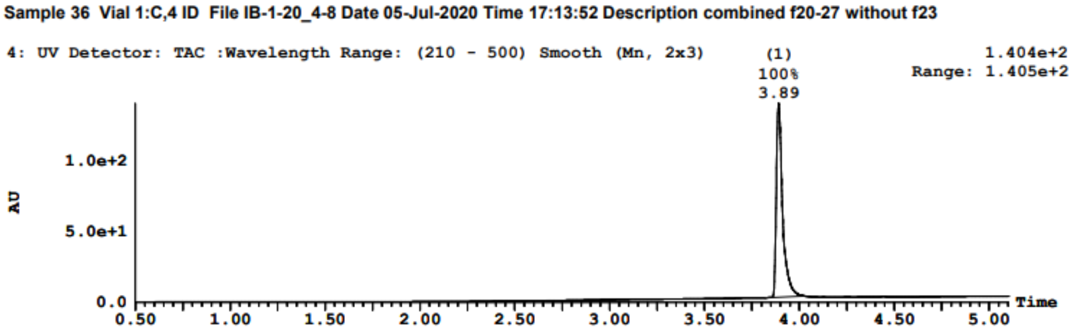
Sample 36 Vial 1:C,4 ID File IB-1-20_4-8 Date 05-Jul-2020 Time 17:13:52 Description combined f20-27 without f23
4: UV Detector: TAC :Wavelength Range: (210 - 500) Smooth (Mn, 2x3)
(1)
100%
3.89
1.404e+2
Range: 1.405e+2
AU
1.0e+2
5.0e+1
0.0
Time
0.50
1.00
1.50
2.00
2.50
3.00
3.50
4.00
4.50
5.00

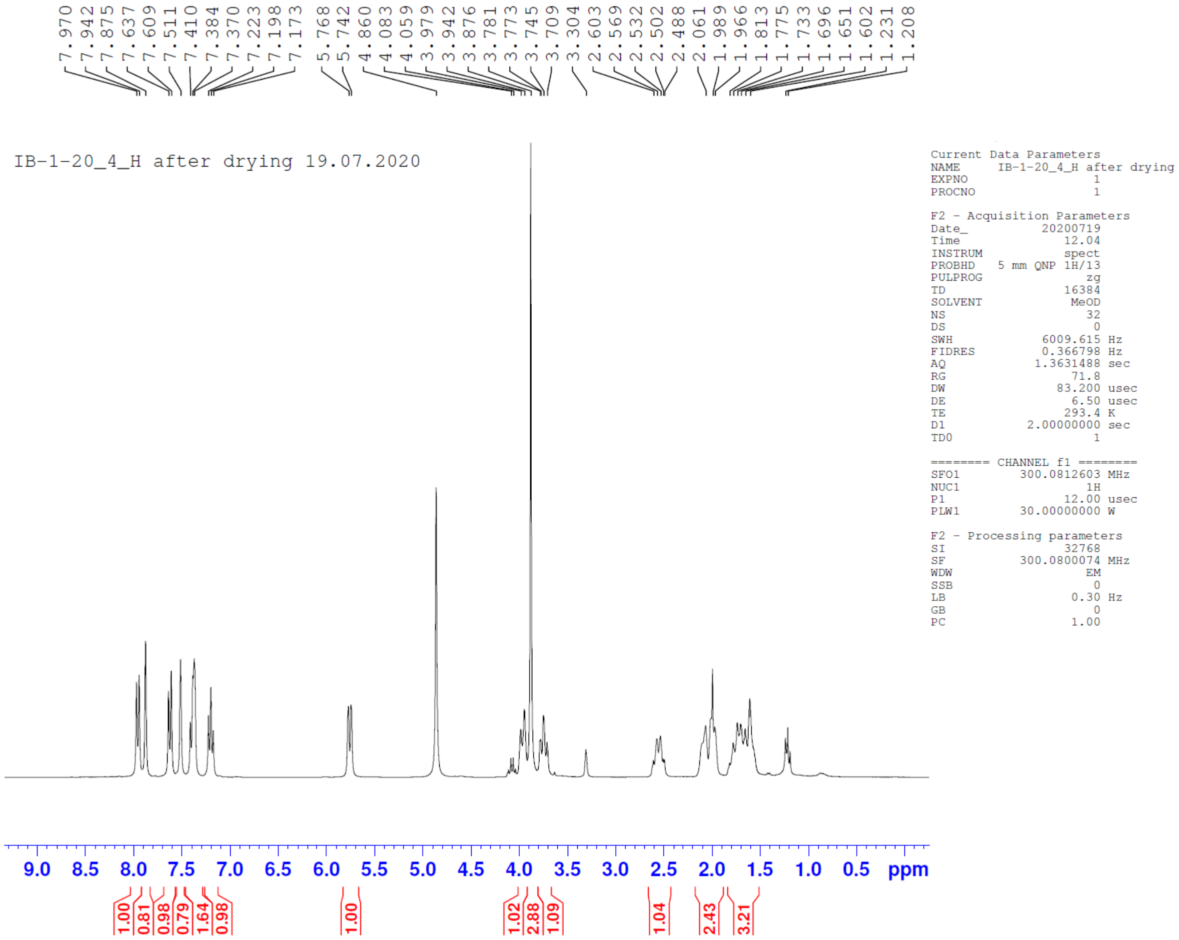
7.970
7.942
7.875
7.637
7.609
7.511
7.410
7.384
7.370
7.223
7.198
7.173
5.768
5.742
4.860
4.083
4.059
3.979
3.942
3.876
3.781
3.773
3.745
3.709
3.304
2.603
2.569
2.532
2.502
2.488
2.061
1.989
1.966
1.813
1.775
1.733
1.696
1.651
1.602
1.231
1.208
IB-1-20_4_H after drying 19.07.2020
Current Data Parameters
NAME IB-1-20_4_H after drying
EXPNO 1
PROCNO 1
F2 - Acquisition Parameters
Date_ 20200719
Time 12.04
INSTRUM spect
PROBHD 5 mm QNP 1H/13
PULPROG zg
TD 16384
SOLVENT MeOD
NS 32
DS 0
SWH 6009.615 Hz
FIDRES 0.366798 Hz
AQ 1.3631488 sec
RG 71.8
DW 83.200 usec
DE 6.50 usec
TE 293.4 K
D1 2.00000000 sec
TD0 1
======== CHANNEL f1 ========
SFO1 300.0812603 MHz
NUC1 1H
P1 12.00 usec
PLW1 30.00000000 W
F2 - Processing parameters
SI 32768
SF 300.0800074 MHz
WDW EM
SSB 0
LB 0.30 Hz
GB 0
PC 1.00
9.0
8.5
8.0
7.5
7.0
6.5
6.0
5.5
5.0
4.5
4.0
3.5
3.0
2.5
2.0
1.5
1.0
0.5
ppm
1.00
0.81
0.98
0.79
1.64
0.98
1.00
1.02
2.88
1.09
1.04
2.43
3.21

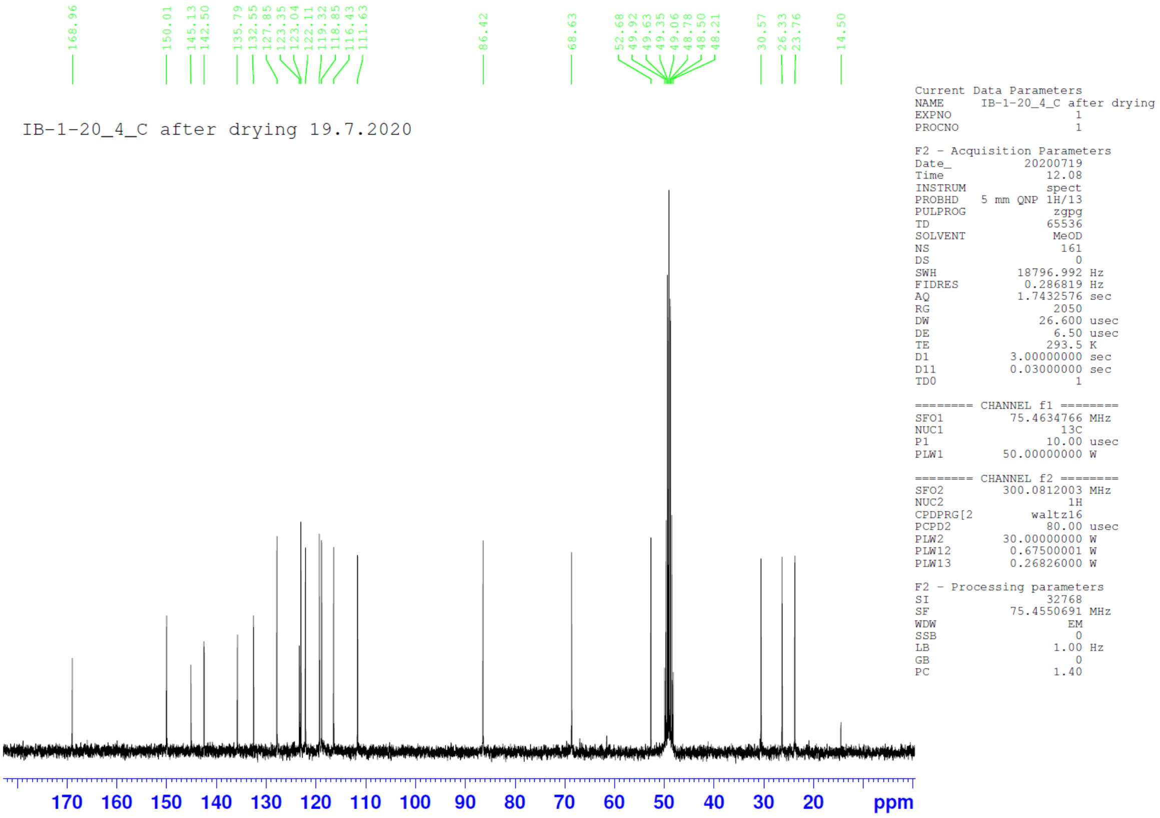

Methyl 3-acrylamido-5-(1-(tetrahydro-2H-pyran-2-yl)-1H-indazol-3-yl)benzoate (IB-1-21).

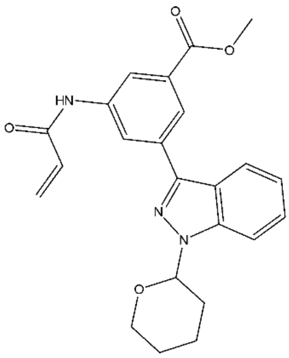

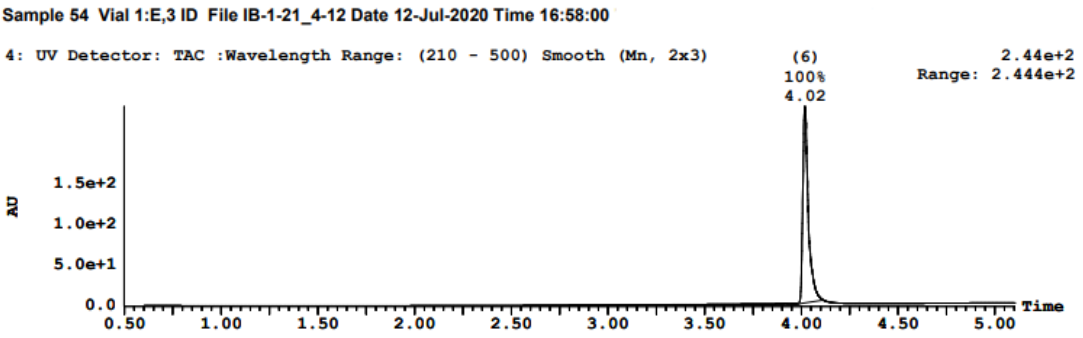
Sample 54 Vial 1:E,3 ID File IB-1-21_4-12 Date 12-Jul-2020 Time 16:58:00
4: UV Detector: TAC :Wavelength Range: (210 - 500) Smooth (Mn, 2x3)
(6)
100%
4.02
2.44e+2
Range: 2.444e+2
AU
1.5e+2
1.0e+2
5.0e+1
0.0
0.50 1.00 1.50 2.00 2.50 3.00 3.50 4.00 4.50 5.00
Time

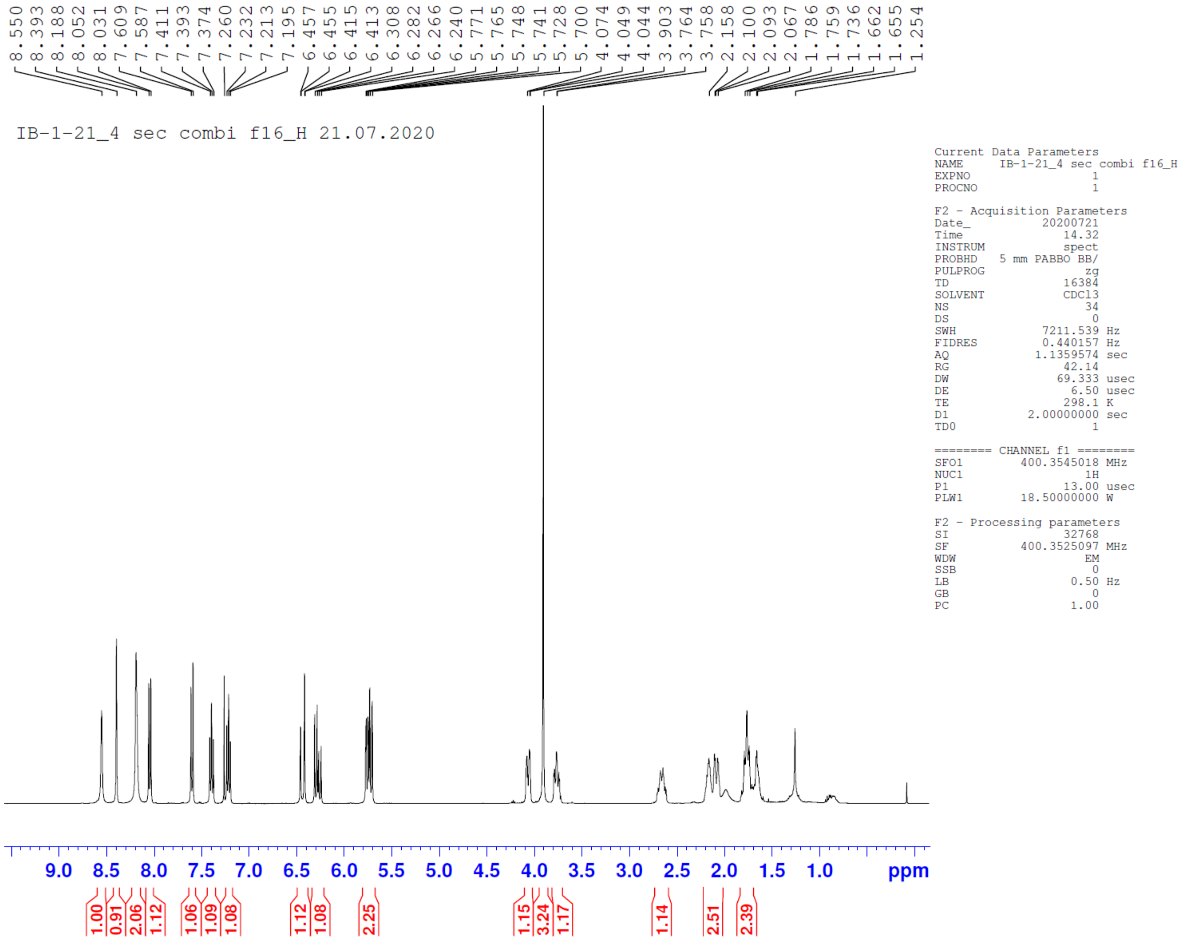
8.550 8.393 8.188 8.052 8.031 7.609 7.587 7.411 7.393 7.374 7.260 7.232 7.213 7.195 6.457 6.455 6.415 6.413 6.308 6.282 6.266 6.240 5.771 5.765 5.748 5.741 5.728 5.700 4.074 4.049 4.044 3.903 3.764 3.758 2.158 2.100 2.093 2.067 1.786 1.759 1.736 1.662 1.655 1.254
IB-1-21_4 sec combi f16_H 21.07.2020
Current Data Parameters
NAME IB-1-21_4 sec combi f16_H
EXPNO 1
PROCNO 1
F2 - Acquisition Parameters
Date_ 20200721
Time 14.32
INSTRUM spect
PROBHD 5 mm PABBO BB/
PULPROG zg
TD 16384
SOLVENT CDCl3
NS 34
DS 0
SWH 7211.539 Hz
FIDRES 0.440157 Hz
AQ 1.1359574 sec
RG 42.14
DW 69.333 usec
DE 6.50 usec
TE 298.1 K
D1 2.00000000 sec
TD0 1
======== CHANNEL f1 ========
SFO1 400.3545018 MHz
NUC1 1H
P1 13.00 usec
PLW1 18.50000000 W
F2 - Processing parameters
SI 32768
SF 400.3525097 MHz
WDW EM
SSB 0
LB 0.50 Hz
GB 0
PC 1.00
9.0 8.5 8.0 7.5 7.0 6.5 6.0 5.5 5.0 4.5 4.0 3.5 3.0 2.5 2.0 1.5 1.0 ppm
1.00 0.91 2.06 1.12 1.06 1.09 1.08 1.12 1.08 2.25 1.15 3.24 1.17 1.14 2.51 2.39

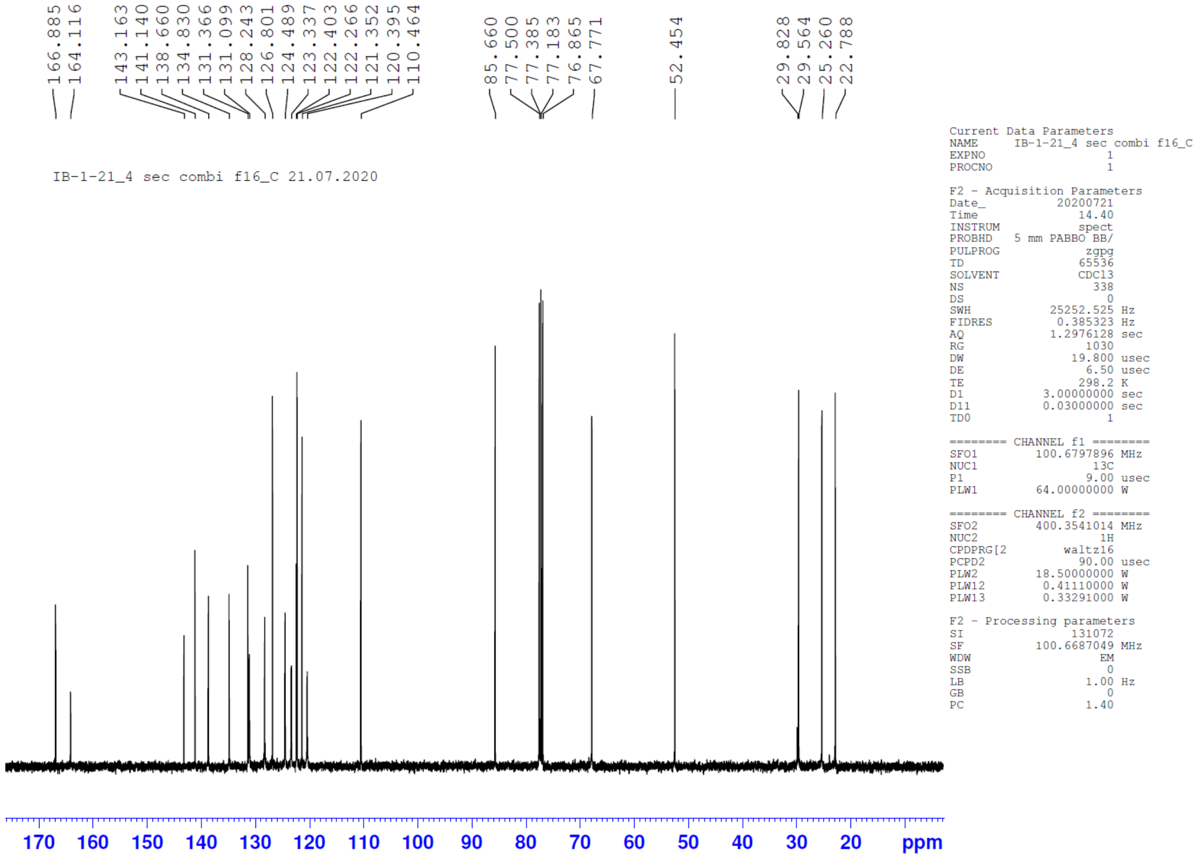

3-acrylamido-5-(1-(tetrahydro-2H-pyran-2-yl)-1H-indazol-3-yl)benzoic acid (IB-1-22).

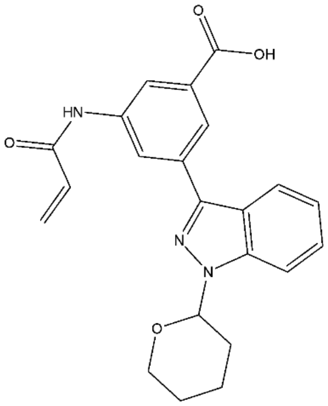

Sample 137 Vial 1:F,8 ID File IB-1-22_5-40 Date 05-Aug-2020 Time 15:46:17 Description after sec combi f20,22,23,24. after heating

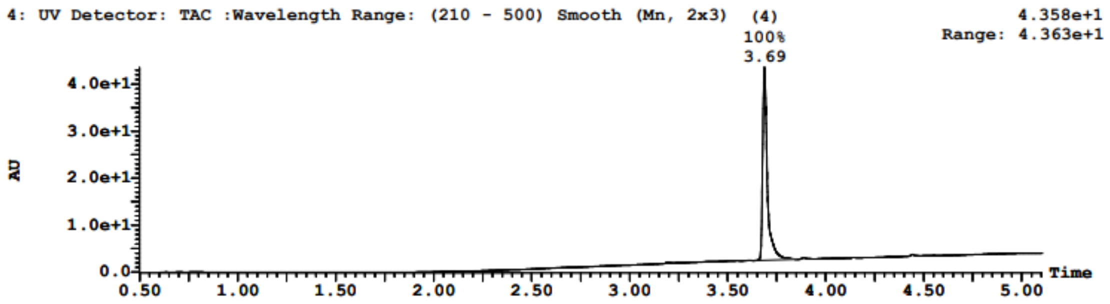

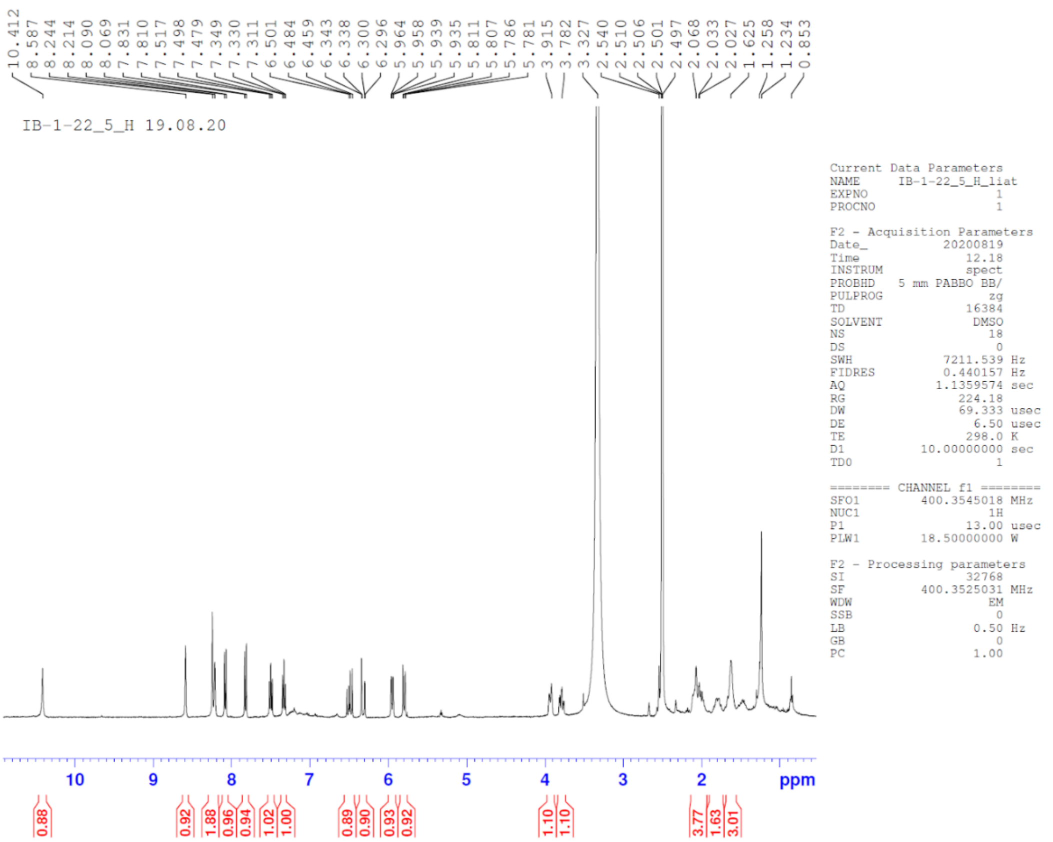

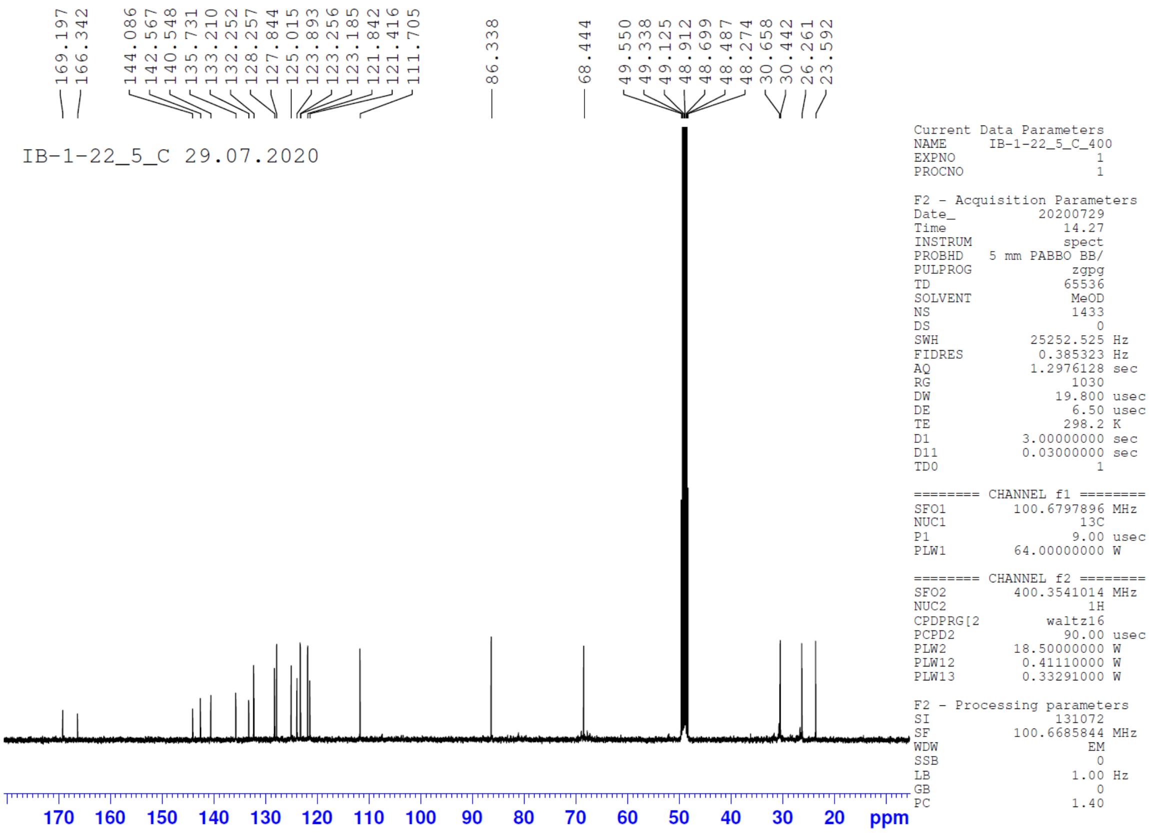

3-acrylamido-N-(prop-2-yn-1-yl)-5-(1-(tetrahydro-2H-pyran-2-yl)-1H-indazol-3-yl)benzamide (IB-1-23).

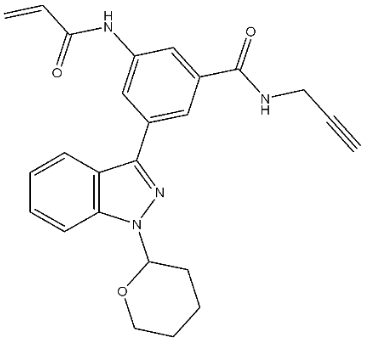

Sample 174 Vial 1:E,2 ID File IB-1-23_4-20 Date 10-Aug-2020 Time 16:07:05 Description after sec f22-23

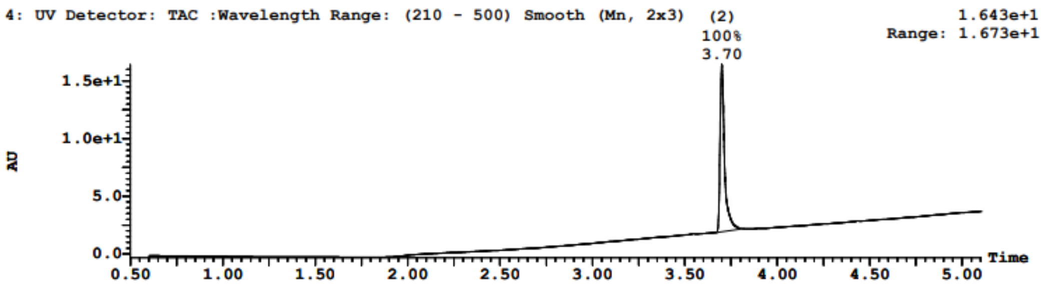

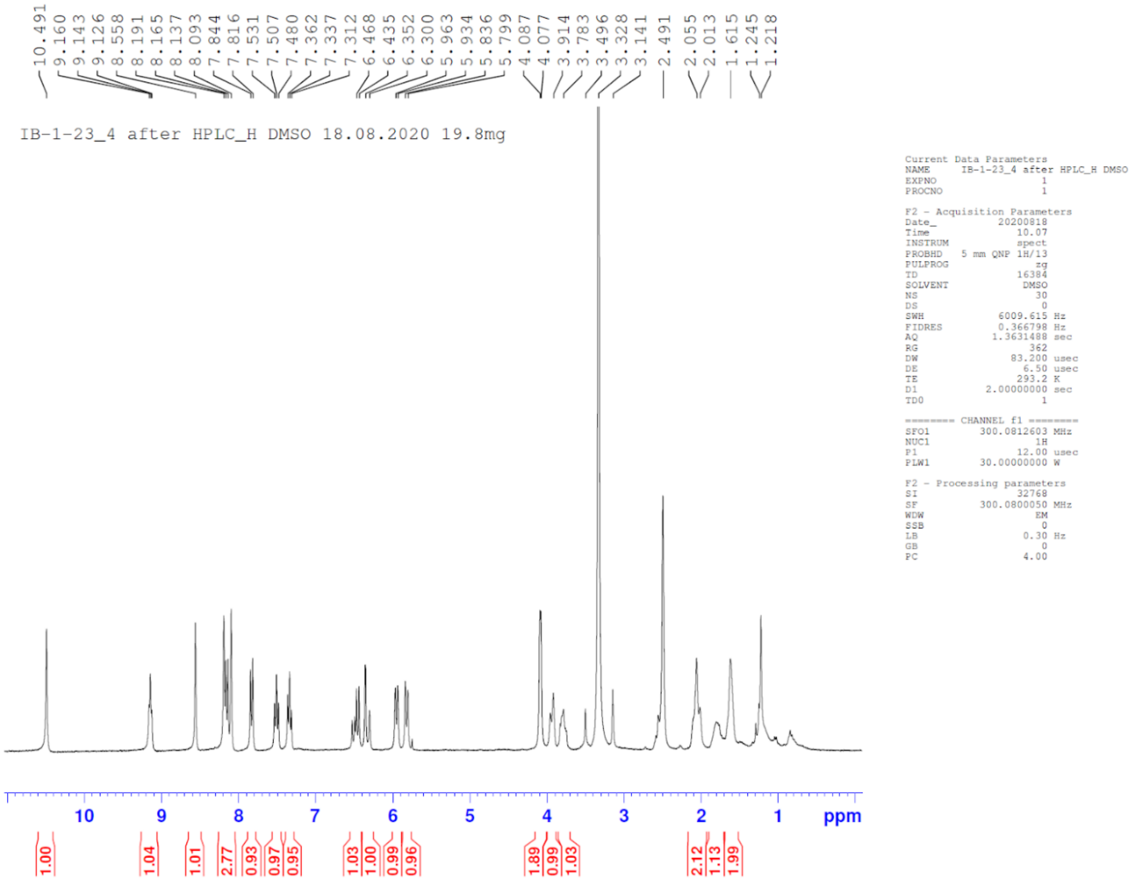

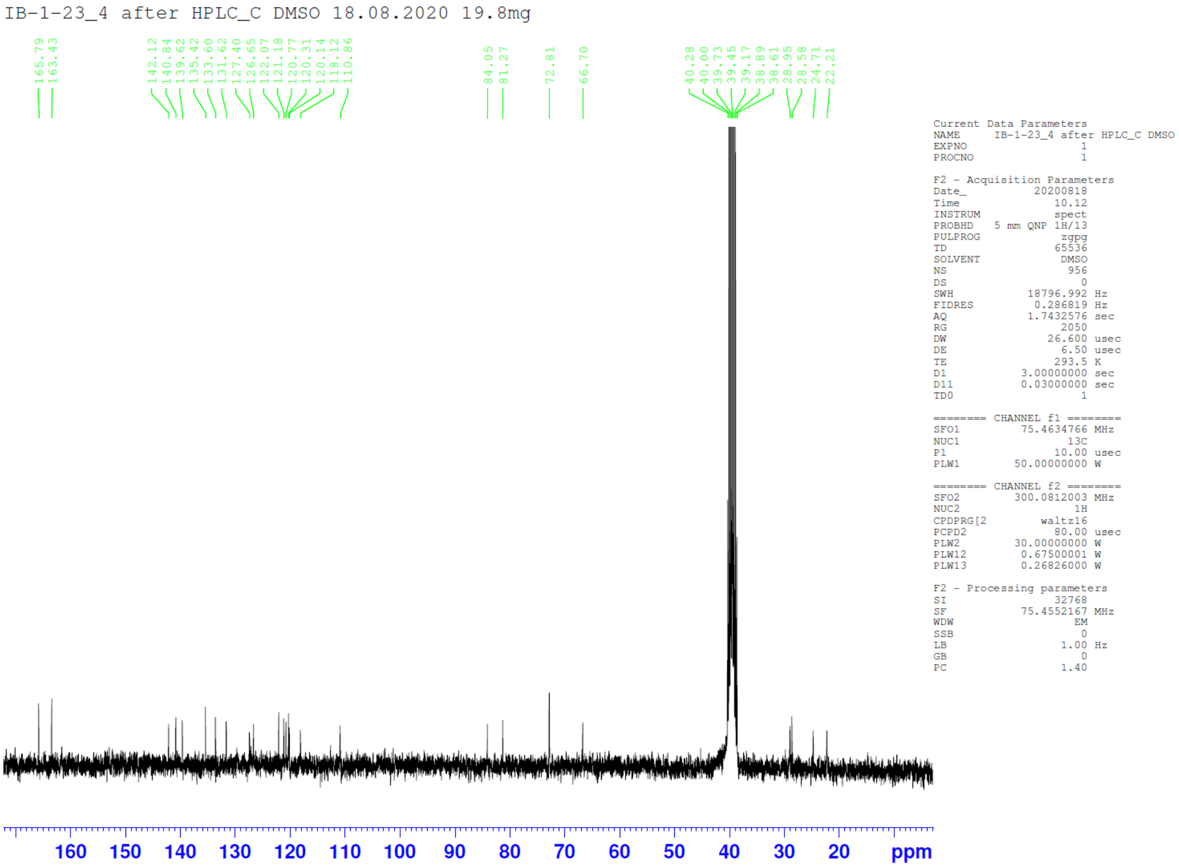

3-acrylamido-5-(1H-indazol-3-yl)-N-(prop-2-yn-1-yl)benzamide (IB-1-24).

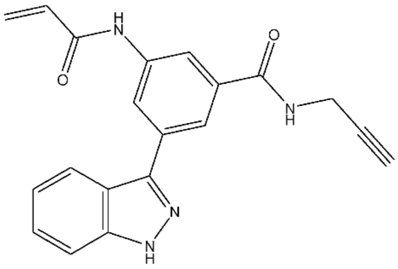

Sample 218 Vial 1:E,6 ID File IB-1-24_3-7 Date 17-Aug-2020 Time 17:04:14 Description after combi f11

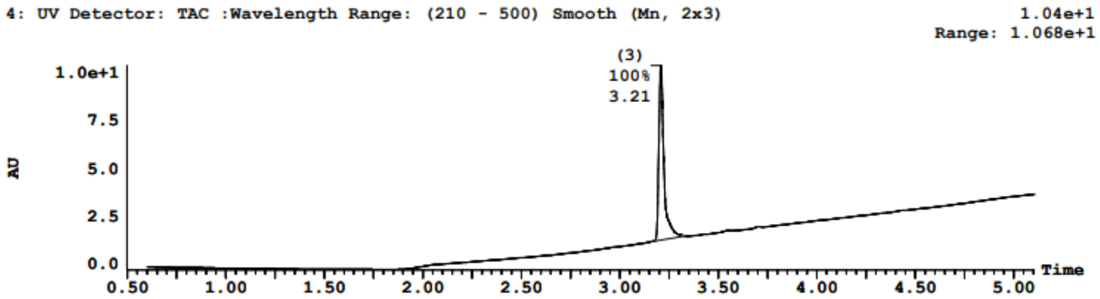

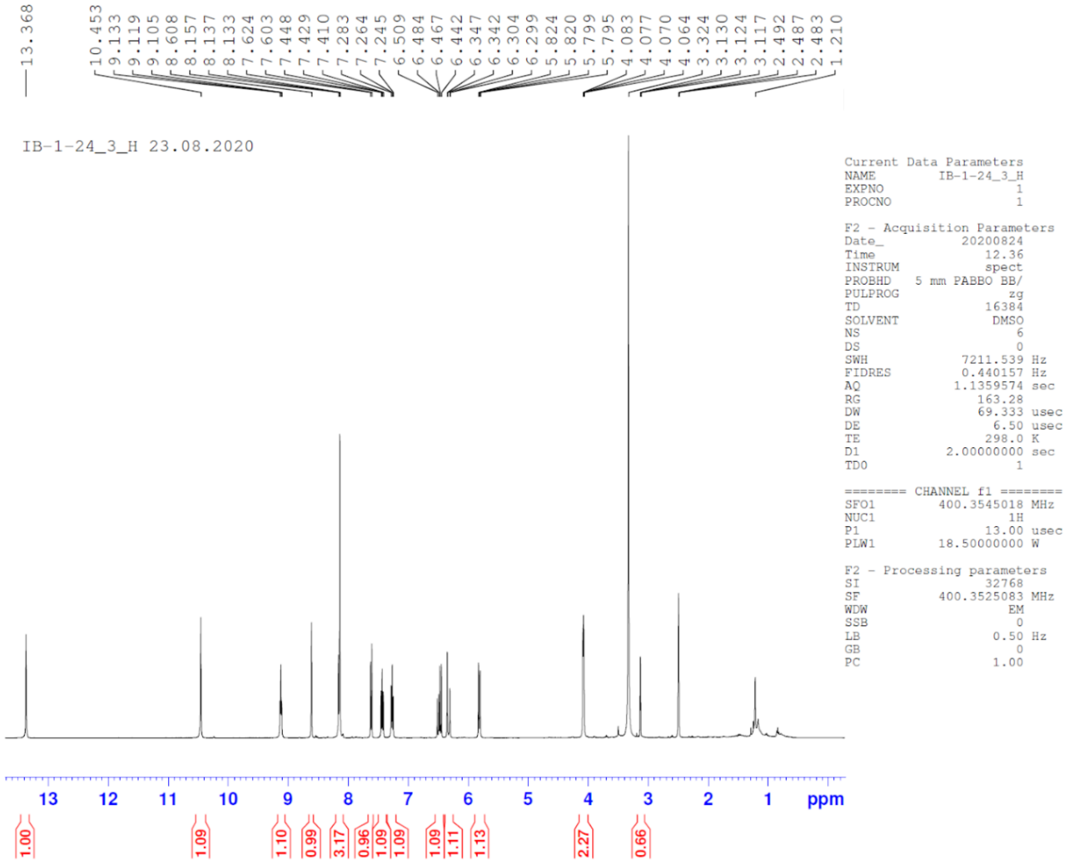

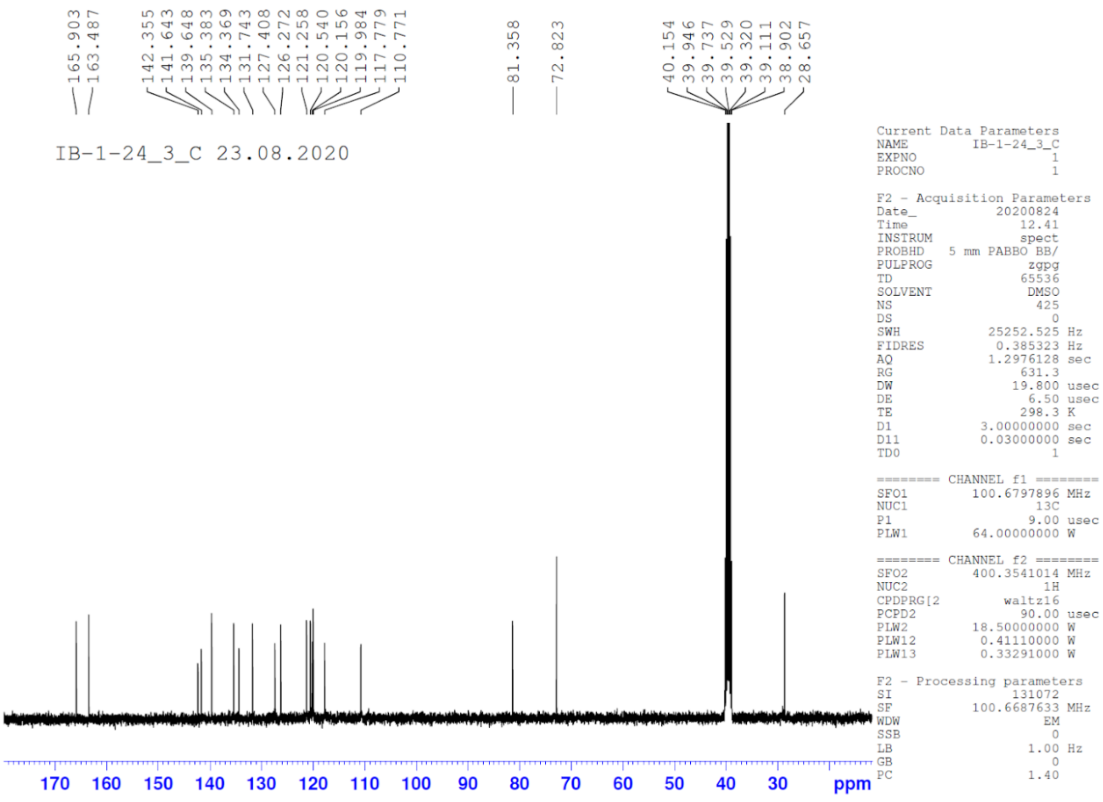

***Rac*-3-acrylamido-5-(1H-indazol-3-yl)-N-((1-((*trans*-)-2-methoxycyclohexyl)-1H-1,2,3-triazol-4-yl)methyl)benzamide** (**2a**)

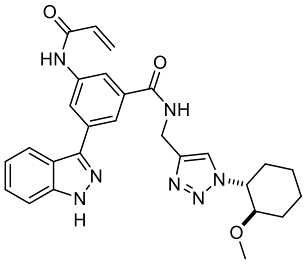

UPLC-MS chromatogram (averaged from 200 – 498 nm)

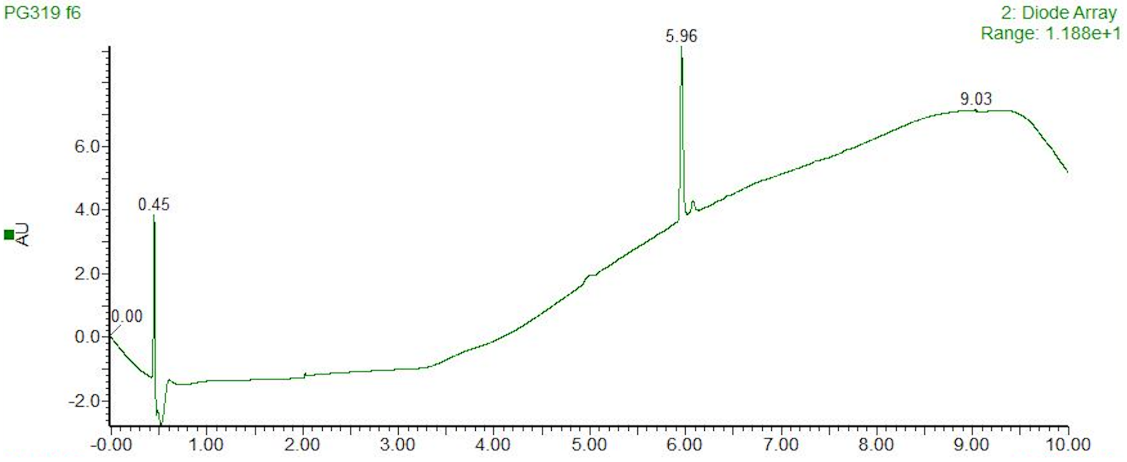

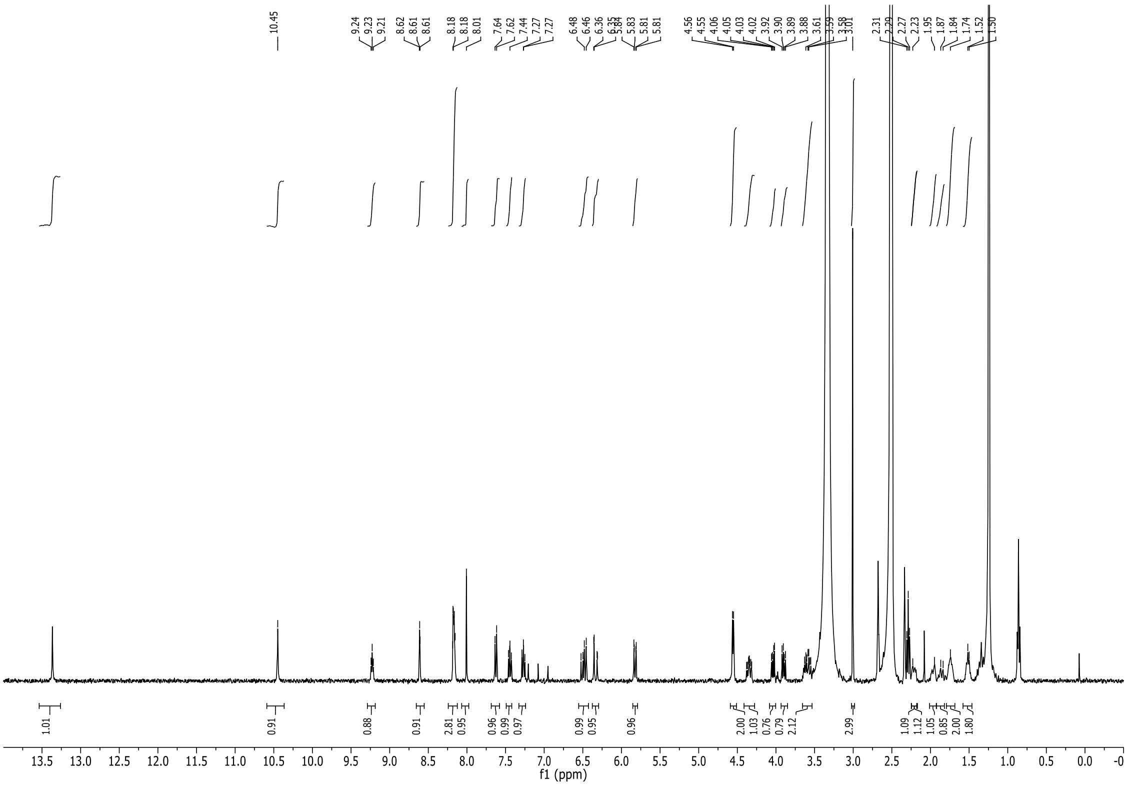

3-acrylamido-5-(1H-indazol-3-yl)-N-((1-(o-tolyl)-1H-1,2,3-triazol-4-yl)methyl)benzamide (2b)

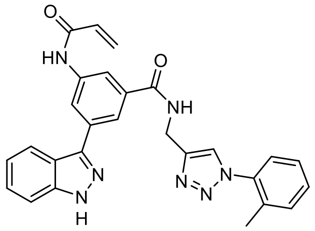

UPLC-MS chromatogram (averaged from 200 – 498 nm)

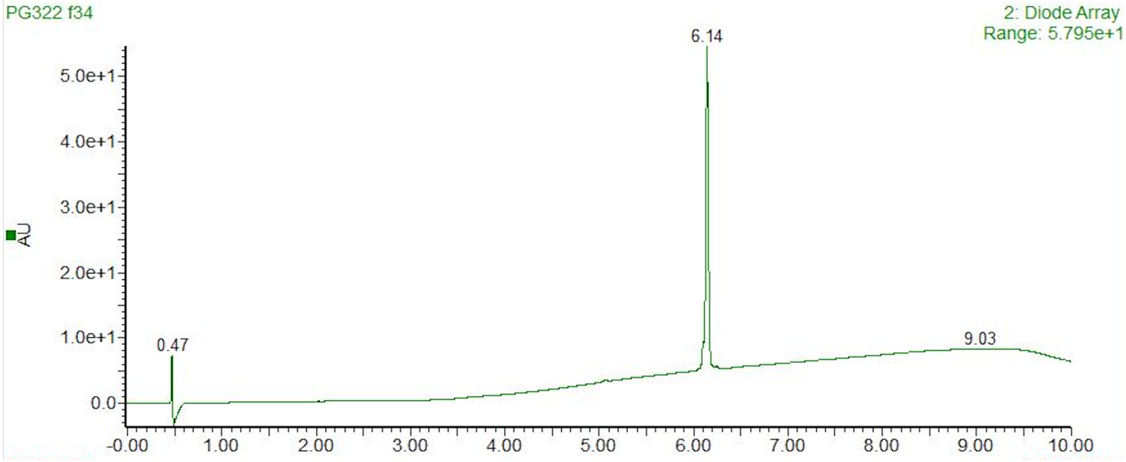

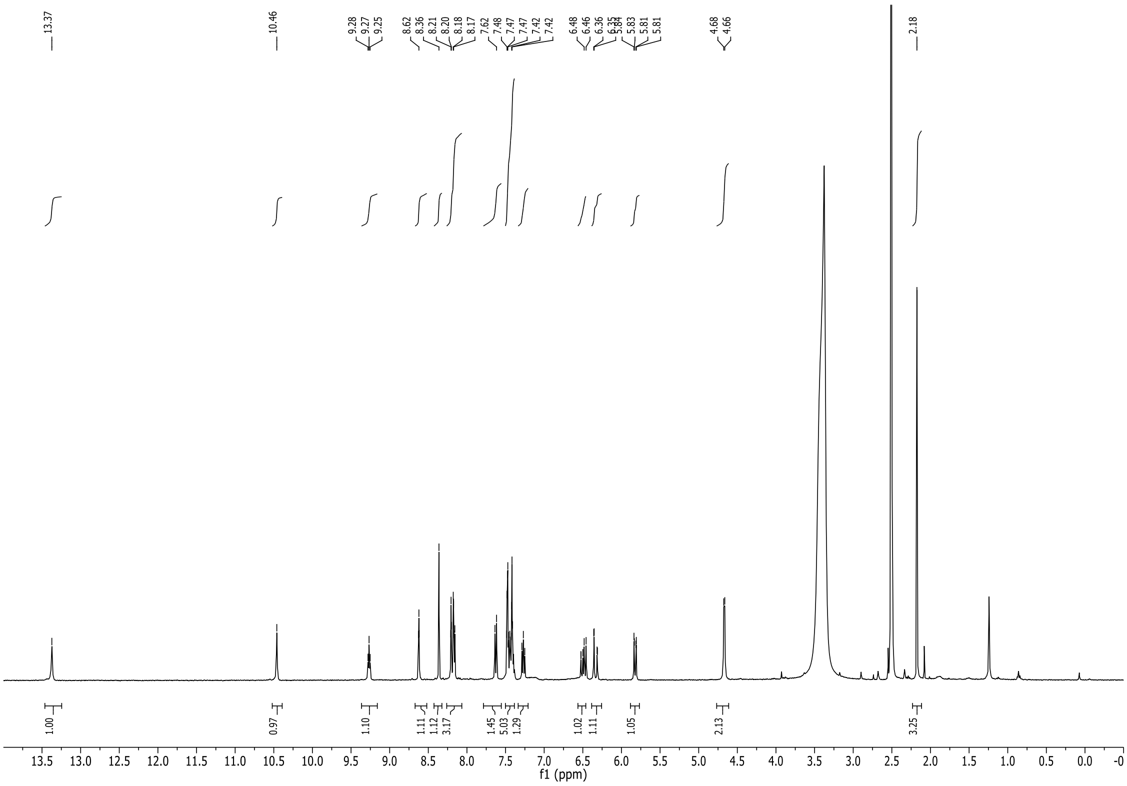

3-acrylamido-N-((1-cyclobutyl-1H-1,2,3-triazol-4-yl)methyl)-5-(1H-indazol-3-yl)benzamide (2c)

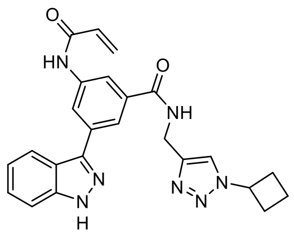

UPLC-MS chromatogram (averaged from 200 – 498 nm)

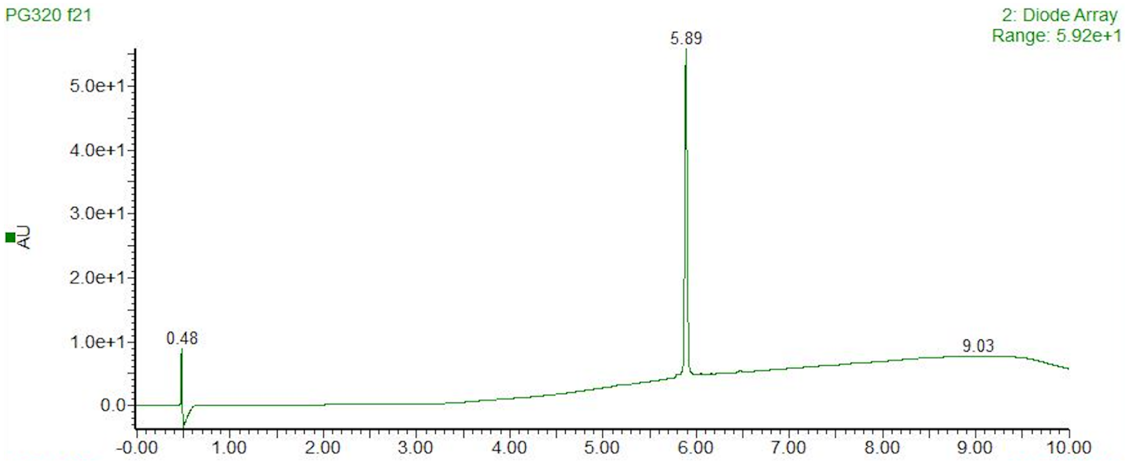

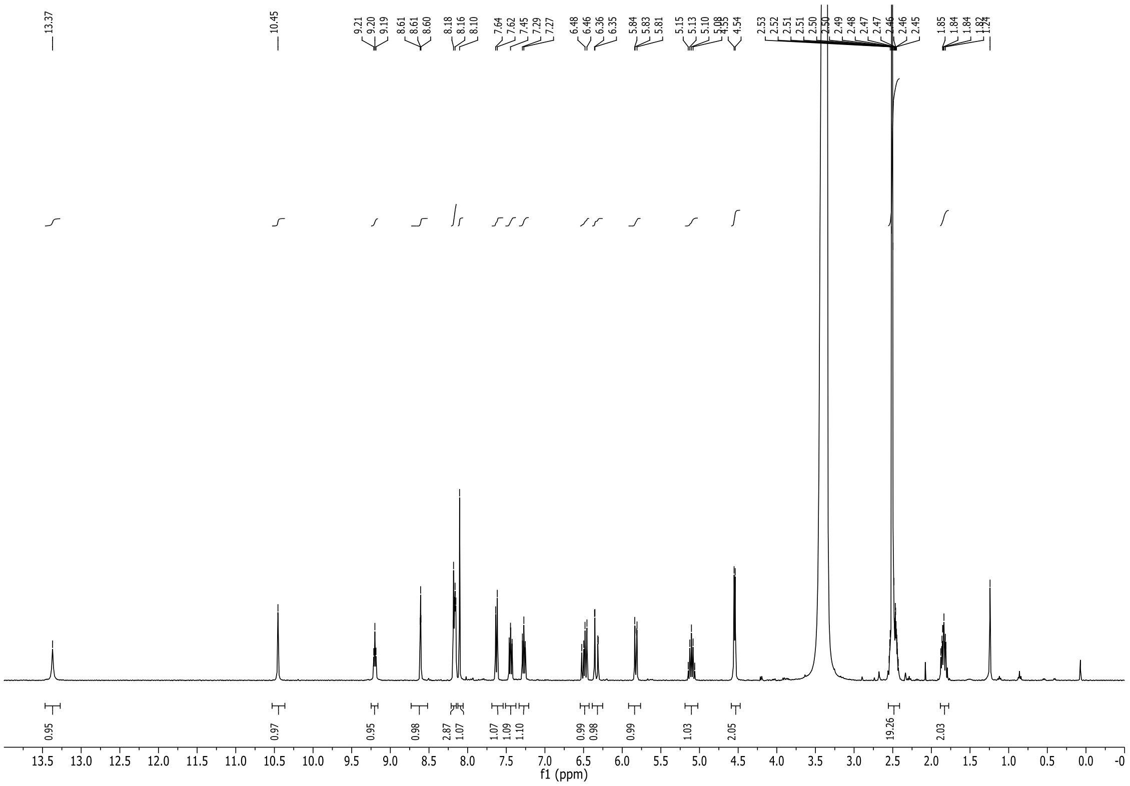

3-acrylamido-5-(1H-indazol-3-yl)-N-((1-(2-methoxyethyl)-1H-1,2,3-triazol-4-yl)methyl)benzamide (2d)

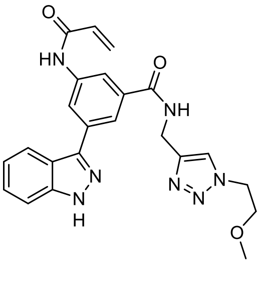

UPLC-MS chromatogram (averaged from 200 – 498 nm)

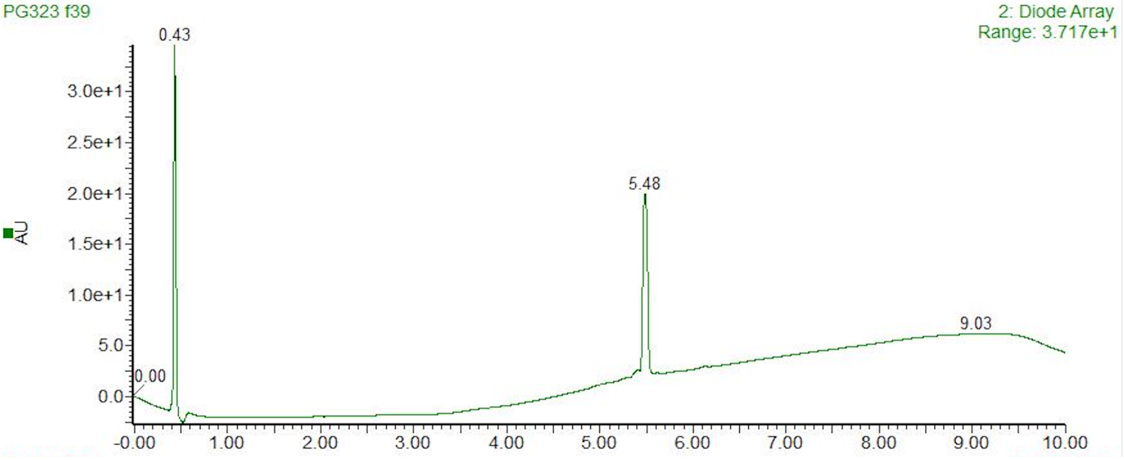

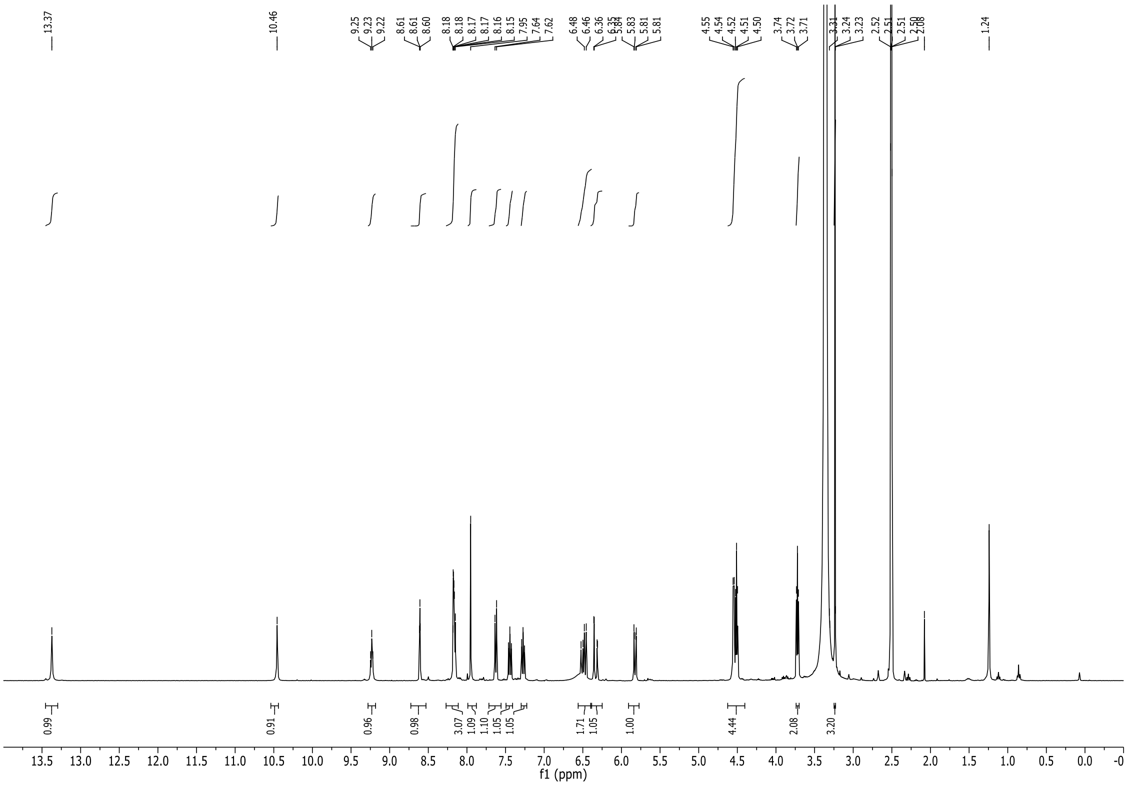

3-acrylamido-N-((1-(3-chloro-4-fluorophenyl)-1H-1,2,3-triazol-4-yl)methyl)-5-(1H-indazol-3-yl)benzamide (2e)

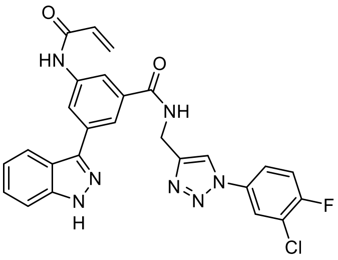

UPLC-MS chromatogram (averaged from 200 – 498 nm)

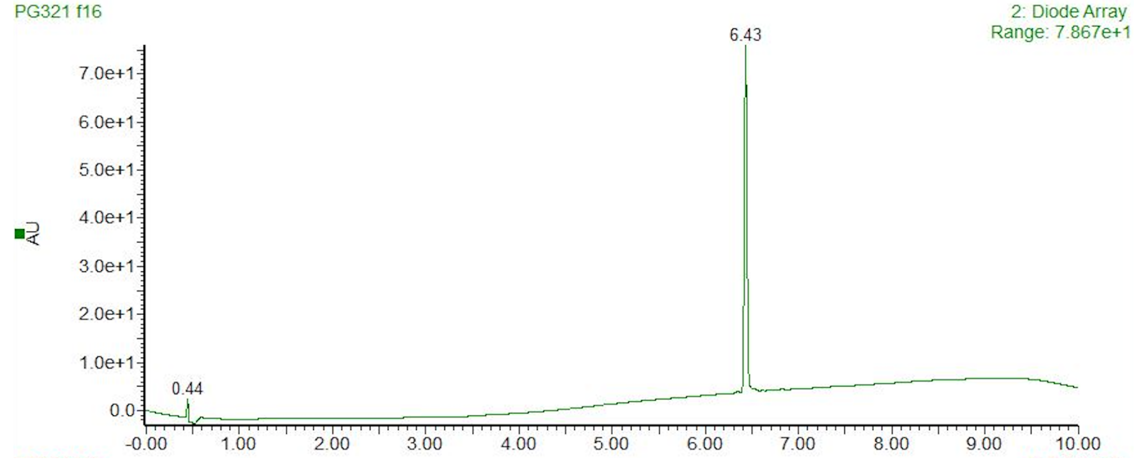

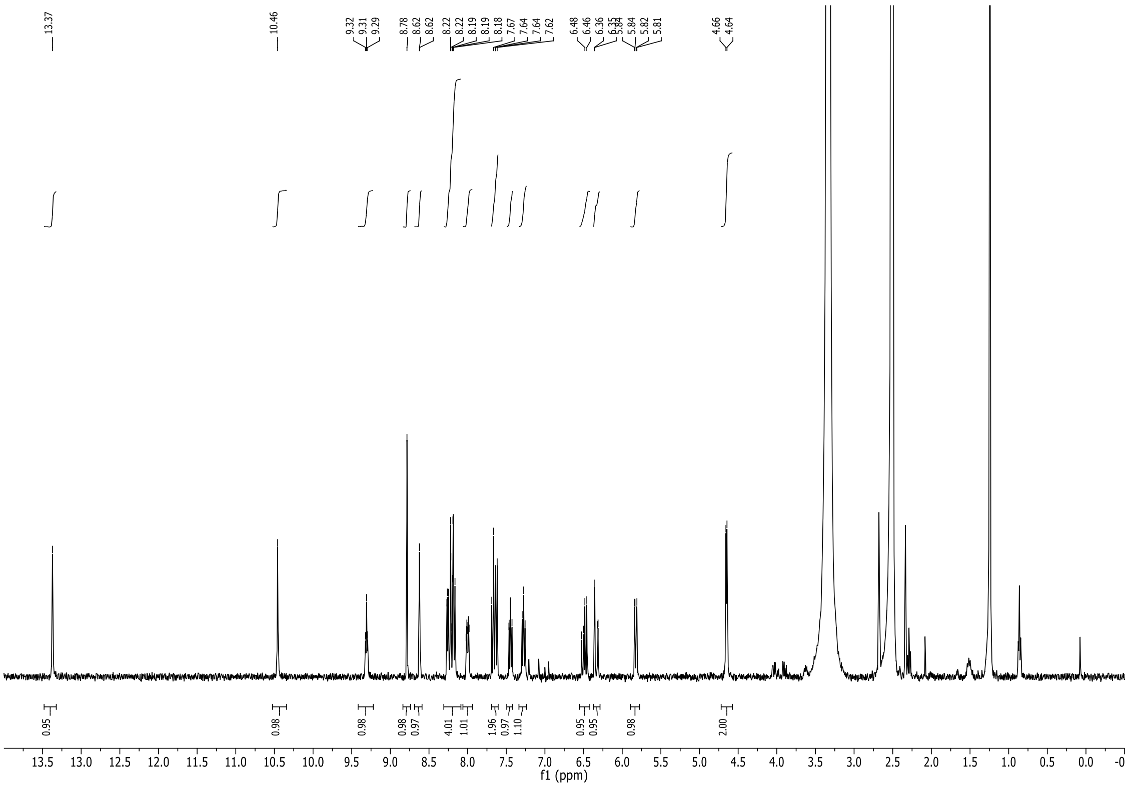

Supplement: Supplementary file 1 — jm1c02206_si_001.pdf [file jm1c02206_si_001.pdf]
